# Origin and dispersion pathways of guava in the Galapagos Islands inferred through genetics and historical records

**DOI:** 10.1002/ece3.8193

**Published:** 2021-10-04

**Authors:** Diego Urquía, Bernardo Gutierrez, Gabriela Pozo, Maria Jose Pozo, Maria de Lourdes Torres

**Affiliations:** ^1^ Laboratorio de Biotecnología Vegetal Universidad San Francisco de Quito (USFQ) Quito Ecuador; ^2^ Department of Zoology University of Oxford Oxford UK; ^3^ Galapagos Science Center Universidad San Francisco de Quito and University of North Carolina at Chapel Hill Galapagos Ecuador

**Keywords:** colonization pathway, Galapagos Islands, genetic diversity, guava, invasive species

## Abstract

Guava (*Psidium guajava*) is an aggressive invasive plant in the Galapagos Islands. Determining its provenance and genetic diversity could explain its adaptability and spread, and how this relates to past human activities. With this purpose, we analyzed 11 SSR markers in guava individuals from Isabela, Santa Cruz, San Cristobal, and Floreana islands in the Galapagos, as well as from mainland Ecuador. The mainland guava population appeared genetically differentiated from the Galapagos populations, with higher genetic diversity levels found in the former. We consistently found that the Central Highlands region of mainland Ecuador is one of the most likely origins of the Galapagos populations. Moreover, the guavas from Isabela and Floreana show a potential genetic input from southern mainland Ecuador, while the population from San Cristobal would be linked to the coastal mainland regions. Interestingly, the proposed origins for the Galapagos guava coincide with the first human settlings of the archipelago. Through approximate Bayesian computation, we propose a model where San Cristobal was the first island to be colonized by guava from the mainland, and then, it would have spread to Floreana and finally to Santa Cruz; Isabela would have been seeded from Floreana. An independent trajectory could also have contributed to the invasion of Floreana and Isabela. The pathway shown in our model agrees with the human colonization history of the different islands in the Galapagos. Our model, in conjunction with the clustering patterns of the individuals (based on genetic distances), suggests that guava introduction history in the Galapagos archipelago was driven by either a single event or a series of introduction events in rapid succession. We thus show that genetic analyses supported by historical sources can be used to track the arrival and spread of invasive species in novel habitats and the potential role of human activities in such processes.

## Graphical abstract

We aimed to reconstruct the invasion pathway of *Psidium guajava* in the Galapagos Islands, as well as to determine its origins in mainland Ecuador. Interestingly, our results were able to connect genetic data and historical data of human colonization of the islands with the dispersal of an invasive species in this iconic archipelago.

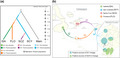

## 1 INTRODUCTION

Invasive species can have a severe impact on biodiversity and ecosystems (Sakai et al., 2001). Invasions are often rapid evolutionary events due to the fact that invasive species can be genetically dynamic; their tolerance and behavior can be modified by natural selection and genetic drift, allowing them to adapt to new environments (Lee & Gelembiuk, 2008; Mooney & Cleland, 2001). Once the new environment is successfully colonized, it can change dramatically as the invader becomes sufficiently abundant and displaces local species, sometimes to the point of extinction (Sakai et al., 2001; Vilà et al., 2011). This gives rise to a reduction in the diversity of local plant species (Vilà et al., 2011).

Invasive species are present in all environments, which are not perennially covered in ice; however, their impact is a particular threat to oceanic islands and archipelagos (Denslow et al., 2009; Mauchamp, 1997; Mooney & Cleland, 2001). These ecosystems are isolated from continental landmasses and cover small areas, and thus present generally low biodiversity. Organisms evolve under different selective pressures on islands compared with their mainland counterparts, which can lead to the emergence of endemic species (Mauchamp, 1997). High endemism and low biodiversity make islands vulnerable to invasive species that are able to compete for resources and adapt to the new ecosystem rapidly. Estimates suggest that islands have twice as many naturalized alien plant species as mainland territories of approximately the same size (Denslow et al., 2009).

Invasive species usually display one or several features that allow them to successfully colonize a new environment: dense local populations, rapid range expansion, high dispersal rates, and high gene flow (Sakai et al., 2001). These features allow the invading species to establish a viable, self‐sustaining population (Cronk & Fuller, 2001; Rejmanek et al., 2013; Sakai et al., 2001). Furthermore, external factors such as the interactions between the invasive species and its community can aid in the process. For instance, invasions are facilitated when the invader has few competitors in the new environment or if the new competitors are different from the ones found in its home range (Cronk & Fuller, 2001; Sakai et al., 2001). Once the species is established in its new territory, human presence can further help it disperse: As an example in plants, species that are useful or ornamental will be cultivated by humans and transported across long distances (Cronk & Fuller, 2001; Sakai et al., 2001). Dispersion can also be promoted by environmental conditions such as the wind or bodies of water, as well as by animals such as birds (Sakai et al., 2001).

An alien plant's invasion success depends greatly on genetic and evolutionary processes (Lee & Gelembiuk, 2008; Rejmanek et al., 2013; Sakai et al., 2001). Certain genetic characteristics within populations facilitate invasion, with one of the most important being genetic variation (Lee & Gelembiuk, 2008). Because changes in genetic frequencies are an important characteristic of colonization, genetic diversity studies are key in understanding how invasive species adapt to a new environment (Cronk & Fuller, 2001; Sakai et al., 2001). Additionally, data indicate that multiple introductions and certain reproduction mechanisms are also beneficial to an invasive species' success, as they allow the species to maintain a significant level of genetic diversity (Lawson Handley et al., 2011).

Discovering the origin of an invasive species is fundamental in understanding its evolutionary history. Due to the fact that an invasive species draws its genetic pool from the population from which it originated, it is important to define the genetic structure of the native population (Xu et al., 2015). Comparing the genetic structure of the invasive and native populations helps reconstruct the invasion history and provides useful evolution models (Keller & Taylor, 2008; Xu et al., 2015). While assessing invasion success, it is also important to understand which selection pressures the invasive species has been subjected to (Xu et al., 2015).

The Galapagos Islands are an oceanic ecosystem with high endemism and unique biodiversity, thus making them vulnerable to invasive species (Bensted‐Smith, 2002; Toral‐Granda et al., 2017). *Psidium guajava*, known as the common guava, originated in Central and South America and is cultivated for its fruit in North, Central and South America, South Asia, and Australia (Sitther et al., 2014). It was introduced in the Galapagos Islands in the late 19th century (Urquía et al., 2019; Velasco, 2002; Walsh et al., 2008), where it has become invasive after settling in both cultivated and uncultivated areas, including disturbed areas and natural forests (Mauchamp, 1997; Tye et al., 2007; Walsh et al., 2008). It is currently widely distributed in Galapagos and can be found in all the human‐inhabited islands: San Cristobal, Santa Cruz, Isabela, and Floreana (Tye et al., 2007; Urquía et al., 2019; Walsh et al., 2008).

In previous research, we used historical sources combined with a genetic analysis of the guava populations from San Cristobal, Santa Cruz, and Isabela to reconstruct the invasion history of this species on these three islands. The results suggested that the species was originally introduced in San Cristobal, from where it moved to Isabela and finally to Santa Cruz, with a probable second independent introduction into Isabela (Urquía et al., 2019). This previous study did not identify the origin of these introductions; thus, the main objective of this research was to identify the most likely sources of the Galapagos guava populations (including the previously unsampled Floreana Island) in mainland Ecuador and to compare the introduced populations in the archipelago with their mainland counterparts. Finally, we update the colonization history of the Galapagos guava by incorporating new information about the mainland and Floreana populations. Considering that seen in other invasion processes (e.g., Novak & Mack, 2001) and the results of our previous research, we expected the sources of the introduced populations and its spreading pathway in the archipelago to be compatible with the origin of the first settlers of the Galapagos and the colonization history of these islands. In this way, we also expect this research adds to the knowledge of how human activities have contributed to the spread of invasive plants.

## 2 MATERIALS AND METHODS

### 2.1 Sampling, DNA extraction, and SSR amplification

**FIGURE 1:**
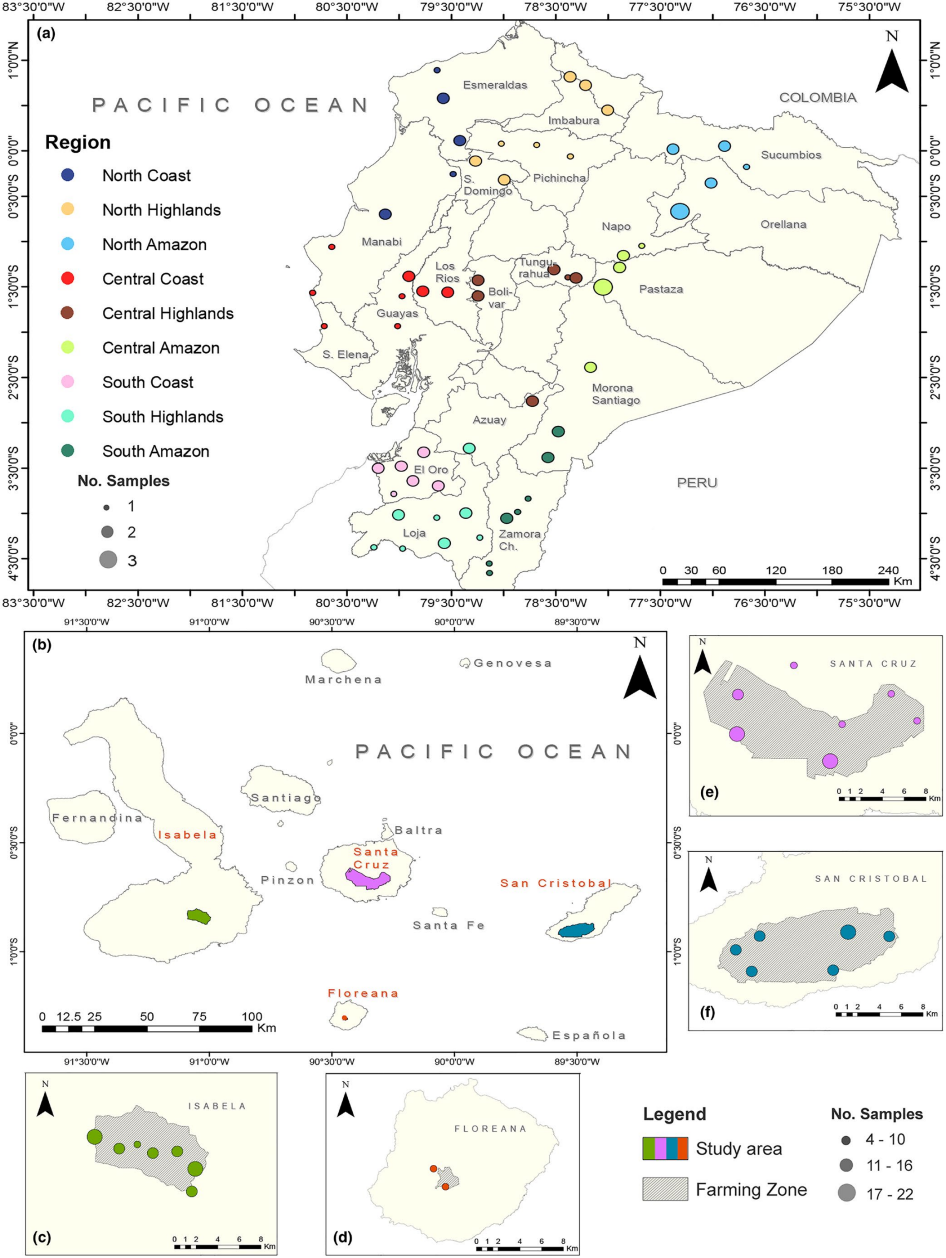
Map representing the guava sampling sites in (a) Mainland Ecuador (including the political limits and names of provinces where sampling was done), (b) the whole Galapagos Archipelago (showing in red labels the islands where guava is present), (c) Isabela Island, (d) Floreana Island, (e) Santa Cruz Island, and (f) San Cristobal Island. The diameter of each mark is proportional to the number of samples obtained from each site. Different colors denote different mainland regions or island population. Maps were drawn using ArcGIS Desktop 10.2 (ESRI, CA, USA)

376 individuals of *Psidium guajava* were sampled for this study: 96 from mainland Ecuador and 280 from the Galapagos Islands (Figure 1). Samples were grouped into five populations: Mainland (96 individuals), Santa Cruz (SCZ, 80 individuals), Isabela (ISA, 95 individuals), San Cristobal (SCY, 94 individuals), and Floreana (FLO, 11 individuals). Due to the significantly larger surface area of the mainland area of study, its samples were further grouped into nine regions based on the three geographic regions of continental Ecuador (Coast, Highlands and Amazon), as well as a latitudinal division where the extension of the country was measured from the northernmost tip to the southernmost one (720 km), and divided into three latitudinal regions: North, Center, and South, each one with a vertical extension of 240 km (Figure 1). Therefore, the nine mainland regions obtained were as follows: North Coast (NC, 8 individuals), North Highlands (NH, 13 individuals), North Amazon (NA, 12 individuals), Central Coast (CC, 11 individuals), Central Highlands (CH, 11 individuals), Central Amazon (CA, 8 individuals), South Coast (SC, 10 individuals), South Highlands (SH, 13 individuals), and South Amazon (SA, 10 individuals). Collection sites were georeferenced using a Garmin eTrex Legend HCx GPS System (Garmin International Inc., USA). Sampled individuals were separated by a minimum distance of 100 m from one another in order to minimize pseudosampling (Urquía et al., 2019). After confirming the taxonomic identity of samples, two to five young leaves were collected from each individual and either transported to the Molecular Biology and Microbiology Laboratory at the Galapagos Science Center in San Cristobal or to the Plant Biotechnology Laboratory at the Universidad San Francisco de Quito campus in Quito, Ecuador, where they were stored at −20℃.

The CTAB protocol (Saghai‐Maroof et al., 1984) was used to isolate total genomic DNA from each individual, after which the concentration and quality of the DNA was measured using a NanoDrop 1000 Spectrophotometer (Thermo Scientific, USA).

PCRs of 11 SSR regions were performed for all samples using species‐specific primers developed by Risterucci et al. (2005). For amplification, a third fluorescently marked universal primer was incorporated, as described by Blacket et al. (2012). Annealing temperatures for each primer pair were optimized, with 5°C being the ideal temperature for all loci, the only exception was 54°C for the mPgCIR25 locus. The program used was 15 min at 95°C; 30–40 cycles of 30 s at 94°C, 90 s at optimized annealing temperature, 60 s at 72°C, and a final step of 5 min at 72°C. PCR products were labeled with one of four fluorescent dyes: 6‐FAM, VIC, PIC, or NED and genotyped by Macrogen (Seoul, Korea) using an ABI 3130 Genetic Analyzer (Thermo Fisher Scientific, USA). Results were analyzed with the GeneMarker software v 2.4.0 (Softgenetics, State College, PA, USA).

### 2.2 Statistical analyses

#### 2.2.1 HWE and LD

The Hardy–Weinberg equilibrium (HWE) was calculated with the *pegas* package for R version 0.13 (Paradis, 2010). For linkage disequilibrium (LD) statistics, we used the *StrataG* package for R version 2.4.905 (Archer et al., 2016). The *p*‐values for both HWE and LD were corrected using the Benjamini and Yekutieli procedure (BY procedure), which controls false discovery rate (Benjamini & Yekutieli, 2001).

#### 2.2.2 Null alleles

FreeNA software was used to estimate frequency of null alleles using the EM algorithm (Chapuis & Estoup, 2007). Null allele frequency was estimated for each SSR locus and population. The same software was used to calculate *F*_ST_ values using the ENA algorithm for null allele correction. Lastly, FreeNA was used to calculate the Cavalli‐Sforza & Edwards genetic distances (Cavalli‐Sforza & Edwards, 1967) corrected for null allele frequency (*D*_CH_).

#### 2.2.3 Basic diversity statistics

We calculated the total number of alleles (*A*), observed heterozygosity (*H*_O_), expected heterozygosity (*H*_E_), and *F*‐statistics for each SSR locus using the *adegenet* (Jombart & Ahmed, 2011) and *hierfstat* (Goudet, 2005) packages for R. Private alleles (PA) were determined using the *poppr* package (Kamvar et al., 2014). Mean allelic richness (AR), standardized through rarefaction, was calculated with HP‐RARE v1.1 (Kalinowski, 2005); for the comparison among Galapagos and mainland populations, an *N* = 160 (genes) was considered for rarefaction standardization, and an *N* = 16 for the comparison among mainland regions. Due to the fact that null allele frequency was elevated, especially for the mainland and Isabela populations, we calculated *H*_E_ with corrected allelic frequencies for null alleles using the EM algorithm obtained from FreeNA (Dempster et al., 1977). In order to do so, we modified the *H*_S_ function of the *adegenet* package by supplying the allelic frequencies obtained by FreeNA as input. *F*_IS_ corrected for null alleles was also calculated using the corrected *H*_E_.

In order to determine whether the *H*_E_ is significantly different between populations, we performed a *H*_E_ test. We analyzed the *H*_E_ differences between populations based on both the raw and corrected *H*_E_ estimates. For the uncorrected *H*_E_ estimates, we used the Hs.test provided by the *adegenet* package, using 999 permutations. When multiple comparisons (i.e., when comparing the *H*_E_ of the four different islands) were performed, the *p*‐value was corrected using the BY procedure. For the corrected *H*_E_ estimates, we used the Wilcoxon signed‐rank test to compare the global Galapagos *H*_E_ and mainland *H*_E_ estimates. When comparing the corrected *H*_E_ estimates between the four different islands, we used Mood's Median test.

#### 2.2.4 Demographic history of guava in the Galapagos

*Migraine* software (Leblois et al., 2014) was used to detect bottlenecks, changes in population size, and founder events and make demographic estimates using a maximum‐likelihood analysis (maximum‐likelihood estimation, MLE). For each Galapagos population, we ran the software under the *OnePopVarSize* demographic model (a single population model with a single continuous past variation in population size) and the stepwise mutation model (SMM) in order to define the bounds of the different demographic parameters: Current effective population size (2*Nµ* current), scaled duration of population size change (*D*_g_/2*N*), and ancestral effective population size (2*Nµ* ancestral). These preliminary runs were performed in 4 iterations, with 500 point estimates and 2000 runs per point. Once the bounds were defined, we performed our inferences using the *OnePopFlounderFlush* demographic model (two variations in past population size) and the stepwise mutational model (SMM); we performed 14 iterations with 500 point estimates and 5000 runs per point.

The output of the *OnePopFlounderFlush* model includes MLEs for different ratios between demographic parameters: (a) the current population/ancestral population ratio, termed the “*N*‐ratio curr‐anc”; (b) the current population/founding population ratio, termed the “*N*‐ratio curr‐found”; and (c) the founding population/ancestral population ratio, “*N*‐ratio found‐anc.” These ratios measure the magnitude of change in population size and can therefore be used to detect population expansions and contractions, including founding events (which are population contractions between the ancestral and founder populations). To test the differences between the *N*‐ratios, we checked that the 95% confidence intervals (CIs) excluded a 1:1 ratio between populations (*N*‐ratio = 1.0) (Leblois et al., 2014; Wereszczuk et al., 2017). We also evaluated the MLEs of the 2*Nµ* for the current, founding, and ancestral populations. Significant changes in population size between the three were established when the 95% CIs of their respective 2*Nµ* estimates did not overlap; thus, this was also used as a criterion to determine the occurrence of population contractions and expansions (Cao et al., 2019). Finally, we evaluated the duration of the change in population size (*D*_g_ *μ*). Both *D*_g_ *μ* and 2*Nµ* are scaled to the mutation rate (*µ*) of SSRs (Rousset & Leblois, 2007).

#### 2.2.5 Population structure analysis

Population structure was inferred by a Bayesian individual‐based clustering approach using STRUCTURE 2.3.4 (Pritchard et al., 2000). Two analyses were performed, a global analysis that included all the samples and a mainland‐specific analysis that included only samples from the continent. An admixture model was applied using individual sampling islands/regions as a prior. The potential number of genetic clusters (*K*) was evaluated between 1 and 10, with 10 independent runs performed for each *K* value. 1,000,000 Markov chain Monte Carlo (MCMC) steps were used, with a 100,000‐step burn‐in period. The optimum value of *K* was determined using the method described by Evanno et al. (2005) as implemented in Structure Harvester (Earl & vonHoldt, 2012), which defines the optimal value for *K* that fits the data based on the rate of change of the log‐likelihood value for the model between successive *K* values. Individual membership coefficients were summarized from independent runs with the program CLUMPAK (Kopelman et al., 2015). The final STRUCTURE graphs were likewise plotted with the *distruct* component of CLUMPAK, using the Greedy search method under 2000 repeats (Rosenberg, 2004).

#### 2.2.6 Putative origins of the Galapagos guava in mainland Ecuador

Different methods were used to determine the continental origin of guava in the Galapagos Islands. Among the more traditional methods, we estimated pairwise *F*_ST_ to find the least genetically differentiated populations between those from individual islands in the Galapagos archipelago and those from different mainland regions, and constructed a PCoA to visualize genetic Euclidian distances among all individuals and NJ trees showing all Galapagos populations and mainland regions. For the latter, two NJ trees were constructed, one based on Nei distances and the other based on *D*_CH_ corrected for null alleles obtained from FreeNA. In both cases, node support was calculated through 1000 nonparametric bootstrap replicates using the boot.phylo function of the *ape* package.

#### 2.2.7 Distinguishing the origin of each guava lineage found in Galapagos (two lineages: Isabela/Floreana and San Cristobal)

To distinguish the origin of each of the two lineages found in Galapagos (the Isabela/Floreana lineage and the San Cristobal lineage; see *Results*), we first detected which regions in mainland Ecuador most frequently present the private alleles found in Isabela as reported by Urquía et al. in 2019. Then, the lowest *D*_CH_ values and Nei distances between regions of continental Ecuador and each Galapagos population were determined. Finally, we used approximate Bayesian computations (ABC) implemented in the DIYABC software (Cornuet et al., 2014) to explore this question. For this pipeline, individuals from mainland Ecuador were divided into 5 lineages as per the results obtained in STRUCTURE to ensure the results are based on actual genetic data in lieu of the initial arbitrarily assigned divisions. This also allowed for a more representative sample size for each continental subgroup (more individuals were assigned to each of the 5 lineages compared to the original 9 groups). Although the optimal *K* value was 4, *K* = 5 subgroups were used instead to separate the lineages from central and southern Ecuador and thus obtain a better resolution. Separate analyses were conducted to determine the origin of the San Cristobal lineage and the Isabela/Floreana lineage; for each, 15 scenarios were compared using an SMM mutation model through 1,500,000 simulations; 5 of these scenarios assumed that the corresponding Galapagos lineage comes from each of the 5 mainland lineages alone, and 10 assumed admixture between every possible pairing of the continental lineages. Default parameters were used, yet based on preliminary runs, adjustments were made on the *N* of the Galapagos population and divergence times priors. Priors employed in the final ABC runs for determining the origin of the Isabela/Floreana and San Cristobal lineages are specified in Appendix S1, Table S1.

Based on preliminary runs, a total of 56 summary statistics were selected for ABC simulations, including number of alleles for each population and each pair of populations, mean genetic diversity for each population, shared allele distance for each pair of populations, and (du)2 distance for each pair of populations. Posterior probabilities (PPs) of each scenario (point estimate + 95% confidence intervals or CIs) were calculated from the higher 1% of the simulations that approximate the observed data based on a linear discriminant analysis on the sum of squares in a logistic regression (the point estimate of the PPs is defined as the intercept of this regression). Posterior distributions (PDs) were calculated from the best scenario, and the model checking implemented in DIYABC was done to test how well this scenario conforms to the observed data considering all summary statistics available. Two model checking approaches were used: (a) direct quantification of the scenario‐simulated data sets that showed a lower value than the observed data set, and (b) PCA for visualizing graphically how observed data fitted the model‐simulated data.

#### 2.2.8 Models to infer the colonization history of guava in the Galapagos

We used the ABC implemented in DIYABC to find the scenario that best explains the colonization pathway of guava from mainland Ecuador to the different islands in the Galapagos archipelago. One problem that arises with ABC is that the number of candidate models to be tested increases drastically with each population considered. Thus, hundreds of different scenarios could be potentially tested from our starting 5 populations (4 islands + mainland). For this reason, we systematically performed our analyses in different stages, each one with a reduced and computationally manageable number of tested scenarios. In the first stage of our ABC analysis, we tested 16 different colonization scenarios (File S1), considering the whole mainland as the source population of the Galapagos guava. To further reduce the number of scenarios tested at this first stage, we considered the Isabela and Floreana populations as a single group (as supported by our results), and we excluded scenarios in which the San Cristobal and Isabela populations originated from the Santa Cruz population (supported in the results of Urquía et al., 2019). Moreover, we made sure to include scenarios compatible with the colonization history of the Galapagos, as well as alternative scenarios. All candidate scenarios were tested using an SMM mutation model through 1,600,000 simulations. Default parameters were used, and based on preliminary runs, adjustments were made on priors (Appendix S1, Table S2). A total of 26 summary statistics were selected for ABC based on preliminary runs; these include the number of alleles for each population, mean size variance for each population and each pair of populations, *F*_ST_ and (du)2 distance for each pair of populations, and admixture summary statistics assuming that the Santa Cruz population comes from the admixture of the Isabela/Floreana and San Cristobal populations. PPs and PDs were calculated, and model checking was run as mentioned above for best scenarios.

For the second stage of our ABC analysis, we chose the best scenario from the previous stage and used these to further dissect the invasion history in finer detail. Firstly, the populations from Floreana and Isabela were separated to determine which island was invaded first. Scenarios considering a second parallel introduction to Isabela and Floreana were also tested, as well as a scenario where guava first arrives in Floreana before the other islands. Santa Cruz was excluded from this stage of the analysis since its place was already consistently determined in our previous work (Urquía et al., 2019) and confirmed in the first stage of the ABC. Thus, a total of 9 scenarios were tested in this second stage using an SMM mutation model through 900,000 simulations (File S2). Priors were based on stage 1 priors (Appendix S1, Table S2). Summary statistics, PPs, PDs, and model checking were calculated and run as mentioned above.

The final colonization model was constructed based on the results from stages 1 and 2, considering all islands as separate the mainland population as the ancestral one. Priors were based on stage 1 and 2 priors (Appendix S1, Table S2). SMM mutation model was used through 100,000 simulations. A total of 26 summary statistics were selected based on preliminary runs and previous stages; these include the number of alleles for each population, mean size variance for each population and each pair of populations, *F*_ST_ and (du) 2 distance for each pair of populations, and admixture summary statistics assuming that the Santa Cruz population comes from the admixture of the Isabela, Floreana, and/or San Cristobal populations. PDs were calculated for the constructed scenario, and model checking was done as well to test how well this scenario conforms to the observed data considering all summary statistics available.

Based on the estimations from our ABC runs (see Section 3.6) and verifying that they are within published parameters for plant SSRs (Bhargava & Fuentes, 2009), a mutation rate of *μ* = 3.5e−04 (per locus per generation) was determined for guava SSRs from this study. This value was then used to scale *D*_g_ *μ* parameters to numbers of generations, and to estimate the time at which changes in population size (i.e., bottlenecks) occurred, a phenomenon expected to coincide or be close to the times of introduction events (Frankham, 1997; Piry et al., 1999).

## 3 RESULTS

### 3.1 SSR genotyping

All 11 SSR loci amplified successfully in both the Galapagos and mainland Ecuador guava samples. This resulted in a total of 152 unique alleles with an average allelic richness of 13.82 over all loci. The 11 loci were polymorphic for the full sample set, but monomorphism was observed for some loci in the Galapagos populations (Appendix S1, Table S3). Overall, all 11 SSR markers were found to be in HWE when assessing our full data set; nevertheless, significant deviations were found in some cases when assessing each population separately (Appendix S1, Table S3). Within each island and mainland region, no LD was detected from the multiple comparisons; the only exceptions were loci mPgCIR09 and mPgCIR22, found to be in LD in the CH mainland region (see *Methods* for populations' and mainland regions' definitions and abbreviations). We found a low to moderate frequency in null alleles along all groups, with frequencies ranging from ~0 to 0.327 and a global mean of 0.155; in average, null alleles had a higher frequency in the mainland and Isabela populations, and a lower frequency in San Cristobal and Floreana (Appendix S1, Table S4).

### 3.2 Genetic diversity: Mainland Ecuador vs. Galapagos

Genetic diversity was higher in the mainland Ecuador guava population than in the Galapagos, in terms of number of alleles, allelic richness (corrected through rarefaction), and *H*_E_ (permutation test, *p* = .001) (Table 1); the same trend was kept after null allele correction for *H*_E_ values (Wilcoxon test, *p *< .001). The mainland population showed a larger number of private alleles (PAs) compared with the Galapagos populations (Table 1); none of the Galapagos populations had more than 3 PAs in the context of the full data set (mainland Ecuador and Galapagos).

**TABLE 1:** Genetic diversity basic statistics of the analyzed guava populations from mainland Ecuador, and Isabela, Santa Cruz, San Cristobal and Floreana Islands (Galapagos): Number of individuals genotyped from each island (*N*), number of alleles found (*A*), mean allelic richness (AR), number of private alleles (PA), observed heterozygosity (*H*_O_), expected heterozygosity/gene diversity (*H*_E_), and inbreeding coefficient (*F*_IS_)

Population	*N*	A	AR^a^	PA^b^	*H*_O_	*H*_E_ ^c^	*F*_IS_ ^c^
Mainland Ecuador	96	146	12.84	99 (102)	0.291	0.794 (0.765)	0.634 (0.623)
Overall Galapagos	280	53	3.78	6 (3)	0.154	0.490 (0.353)	0.686 (0.547)
Isabela (ISA)	95	40	3.60	2 (2)	0.106	0.444 (0.284)	0.761 (0.617)
Santa Cruz (SCZ)	80	35	3.18	3 (3)	0.169	0.476 (0.365)	0.645 (0.488)
San Cristobal (SCY)	94	25	2.27	0 (0)	0.213	0.391 (0.326)	0.455 (0.331)
Floreana (FLO)	11	19	1.73	1 (1)	0.025	0.258 (0.139)	0.903 (0.705)

^a^ Standardized through rarefaction for *N *= 160 genes.

^b^ Values between brackets are the number of private alleles following rarefaction for *N *= 160 genes.

^c^ The values outside the brackets correspond to *H*_E_ or *F*_IS_ under null allele correction. The values between brackets are values without null allele correction.

All mainland groups had roughly a similar level of genetic diversity (Appendix S1, Table S5). In the Galapagos, the Isabela population had the highest number of alleles and rarefaction‐corrected allelic richness among the insular populations, followed by Santa Cruz, San Cristobal, and Floreana (Table 1). Considering values not corrected for null alleles, the Santa Cruz guava population had the highest *H*_E_ within the Galapagos Islands, which was significantly higher than the diversity in San Cristobal (*adegenet* Hs.test‐multiple comparison, *p* = .007), Isabela (*p* = 0.005), and Floreana (*p* = .005); the two lowest *H*_E_ values (Isabela and Floreana) were equivalent (*p* = .056) (Table 1). When correcting for null alleles, the *H*_E_ estimates increase in all insular populations and the differences between them become insignificant (Mood's Median test, *p* = .079). Regardless of the null allele correction, the inbreeding level assessed through the *F*_IS_ was high in the mainland Ecuador and Galapagos populations (Table 1). Overall, we observed the same trends among the uncorrected number of alleles and the rarefaction‐corrected allelic richness, suggesting a little impact of different sampling sizes in our genetic diversity measures.

According to MLEs (Table 2), a significant founder effect (i.e., a population size contraction between the ancestral and founder populations) was detected for the Isabela and Santa Cruz guava populations, while only an “almost significant” (see Wereszczuk et al., 2017) founder effect was obtained for San Cristobal and Floreana. Santa Cruz was the only case where a population expansion following the founder effect was detected. A significant population contraction among the ancestral and the current guava populations was detected in Isabela and San Cristobal, and an “almost significant” contraction was obtained for Santa Cruz (see Wereszczuk et al., 2017); the guava populations from Floreana appeared to maintain a relatively stable population size. According to our demographic estimations, the largest guava population in Galapagos (in terms of effective population size, 2*Nμ*) would be in Santa Cruz (Table 2). Diagnostic plots for the aforementioned analyses show robust short‐scale regressions and likelihood functions (Files S2–S6).

**TABLE 2:** Results of the maximum‐likelihood estimations (MLEs) of genetic data (*migraine* software, OnePopFounderFlush‐SMM model) for bottleneck and demographic inferences on the Galapagos populations of guava

	Isabela (ISA)	Santa Cruz (SCZ)	San Cristobal (SCY)	Floreana (FLO)
*N*‐ratio curr‐anc	**0.024 [0.0039–0.13]**	*0.295 [0.0104–2.832]*	**0.0060 [0.0002–0.238]**	0.0122 [8.37e−07–11.92]
*N*‐ratio curr‐found	9.985 [0.0249–196.70]	**1070.00 [1.783–10019]**	46.45 [0.0002–10333]	8.304 [0.0002–3459190]
*N*‐ratio found‐anc	**0.0024 [0.0004–0.629]**	**0.0003 [6.58e−05–0.0086]**	*0.0001 [3.09e−06–2.82]*	*0.0015 [NA–4.559]*
2*Nμ* current	0.821^1^ [0.246–3.605]	7.177^1^ [0.298–43.63]	0.161^1^ [0.0062–5.472]	0.189 [7.08e−06–NA]
2*Nμ* founder	0.0822 [0.0166–15.76]	0.0067^1,2^ [0.0026–0.197]	0.0035 [0.0001–57.53]	0.0228 [5.42e−07–22.3]
2*Nμ* ancestral	34.14^1^ [12.47–102.2]	24.33^2^ [8.813–78.96]	26.67^1^ [9.145–108.3]	15.55 [3.113–149.3]
*D* (*D*_g_ *μ*)	0.0832 [0.0265–1.111]	0.0179 [0.0079–0.121]	0.0067 [0.0004–0.546]	0.0431 [5.5e−06–1.294]

Note

The point estimates and 95% CIs for different demographic parameters are shown, including *N*‐ratios (current–ancestral, current–founder, founder–ancestral), mutation rate‐scaled effective population size (2*Nμ*: current, founder, ancestral), and mutation rate‐scaled duration of population size change in number of generations (*D*_g_ *μ*, which is also the time when population size change started).

*N*‐ratios in bold are those whose 95% CIs support a significant change in population size.

*N*‐ratios in italics are those whose 95% CIs suggest an almost significant change in population size.

2*Nμ* values with the same superscript (^1,2^) do not overlap on their 95% CIs, supporting a significant change in population size.

### 3.3 Isolation of the Galapagos populations

**FIGURE 2:**
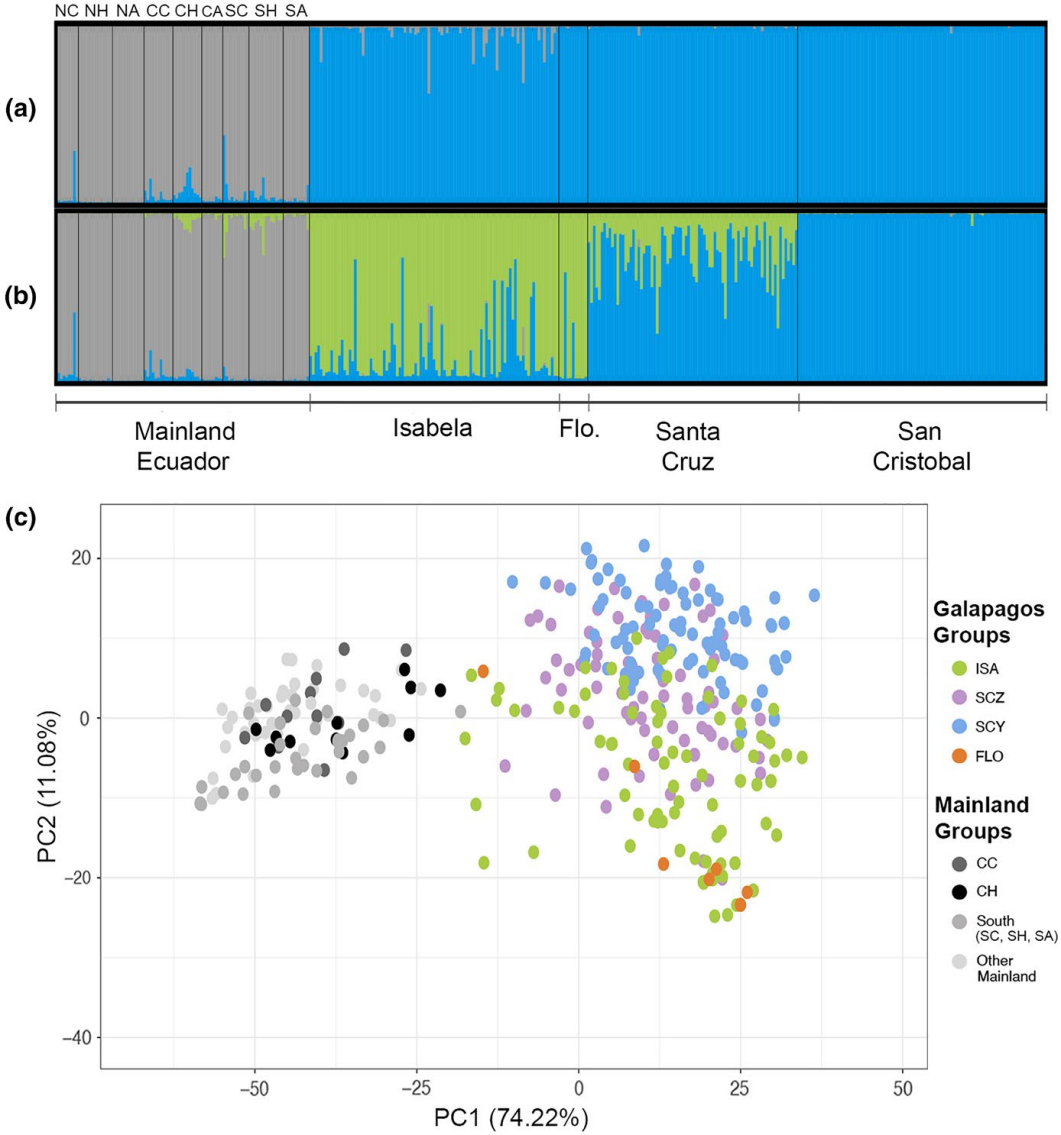
Population structure observed over the full data set including guava populations from mainland Ecuador and the Galapagos. (a) STRUCTURE plot (Bayesian analysis of population structure, Admixture model) for *K* = 2 (best *K* value; Δ*K* = 235.51), (b) STRUCTURE plot (admixture model) for *K* = 3 (second best *K* value, Δ*K* = 7.82), (c) PCoA based on the genetic distances (Euclidian) found between all the individuals sampled. In the structure plots, the values of *K* correspond to the number of clusters (represented by different colors) in which are grouped the sampled individuals. In the PCoA, each color represents a different geographic population. Mainland Ecuador regions are as follows: North Coast (NC), North Highlands (NH), North Amazon (NA), Central Coast (CC), Central Highlands (CH), Central Amazon (CA), South Coast (SC), South Highlands (SH), and South Amazon (SA). Galapagos populations are as follows: Isabela (ISA), Santa Cruz (SCZ), San Cristobal (SCY), and Floreana (FLO)

**FIGURE 3:**
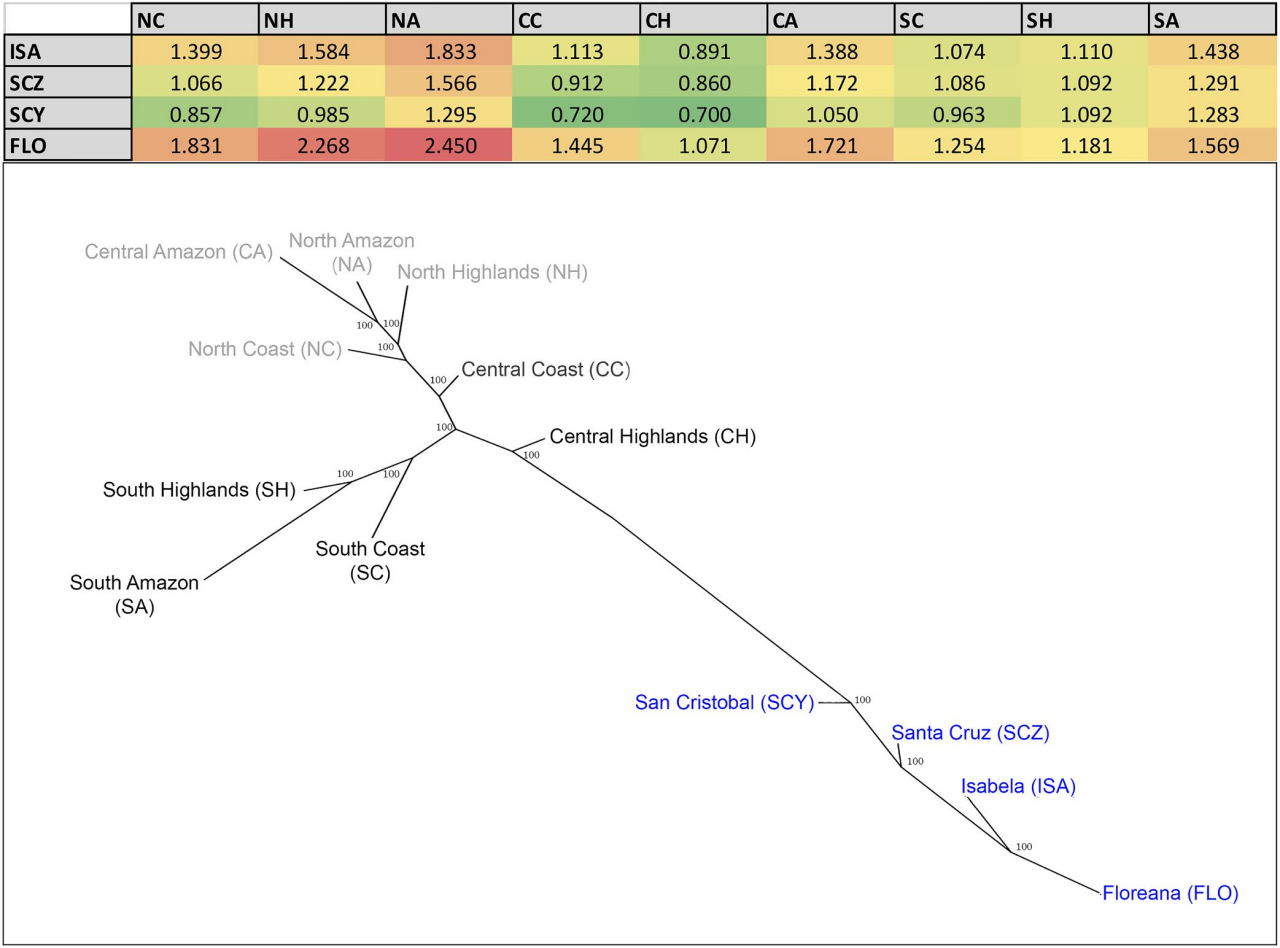
Neighbor‐joining (NJ) tree illustrating Nei's genetic distances among all the mainland Ecuador regions and Galapagos populations of guava; bootstrap values are shown for each node. Galapagos populations are labeled in blue, and mainland regions are labeled in a gray scale, with darker levels showing a greater proximity to the Galapagos populations. A table showing the genetic distances on which the NJ tree was built is also shown, indicating the pairwise distance values among the 4 Galapagos populations and the 9 mainland regions; the table is in a color scale where green cells correspond to lower genetic distances, and red cells correspond to higher distances

**FIGURE 4:**
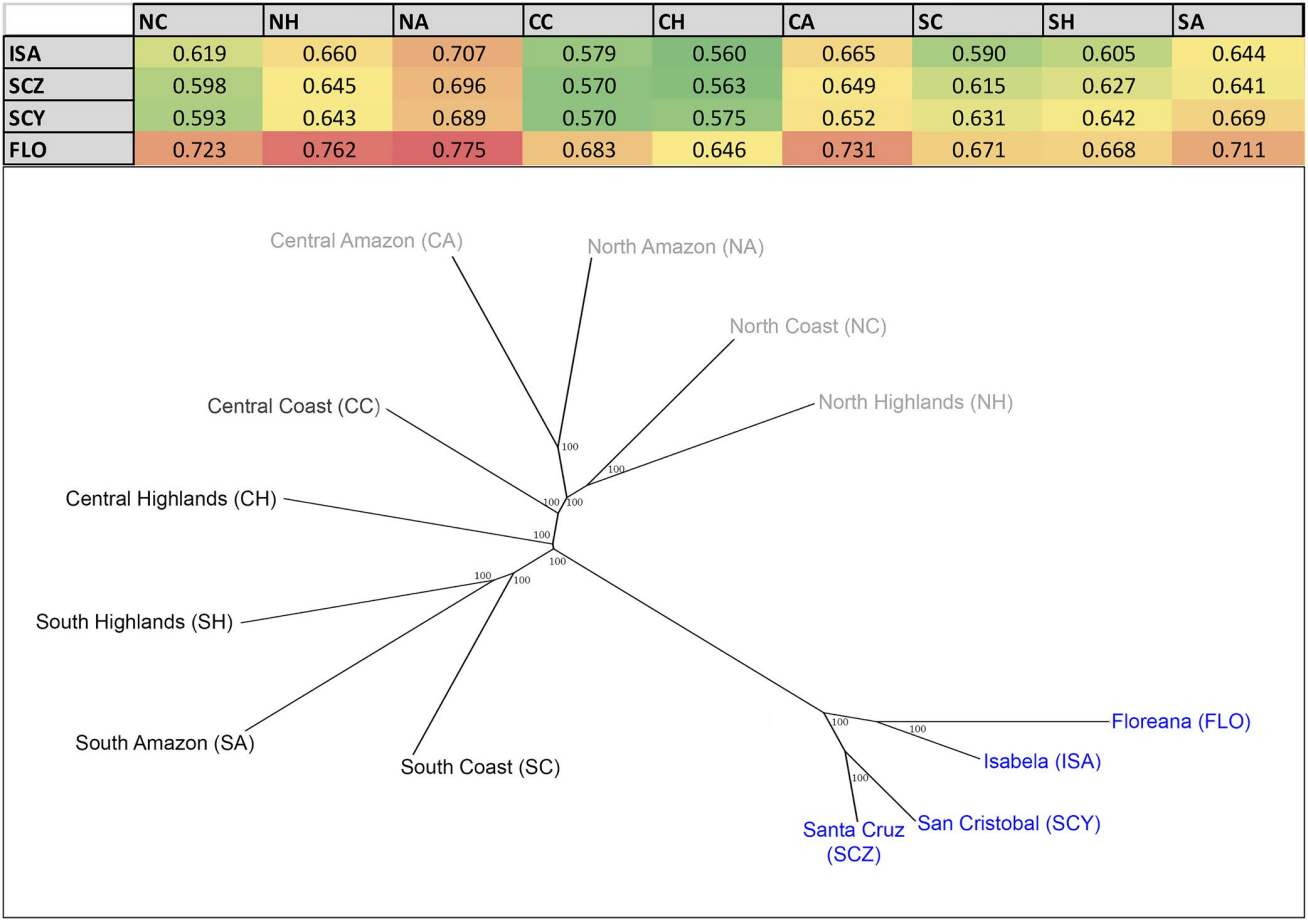
Neighbor‐joining (NJ) tree illustrating the Cavalli‐Sforza & Edwards genetic distances (*D*_CH_) among all the mainland Ecuador regions and Galapagos populations of guava; bootstrap values are shown for each node. Galapagos populations are labeled in blue, and mainland regions are labeled in a gray scale, with darker levels showing a greater proximity to the Galapagos populations. A table showing the genetic distances on which the NJ tree was built is also shown, indicating the pairwise distance values among the 4 Galapagos populations and the 9 mainland regions; the table is in a color scale where green cells correspond to lower genetic distances, and red cells correspond to higher distances

The mainland Ecuador and Galapagos guava individuals were clustered into two clearly separated groups as suggested by the STRUCTURE plots (Figure 2a,b; Appendix S1, Figure S1), the PCoA (Figure 2c), and the NJ trees (Figures 3 and 4). Similarly, when considering the Galapagos and mainland populations together, a significant amount of total variation occurs among populations (Table 3). Pairwise *F*_ST_ estimates among mainland regions and Galapagos populations were consistently high, a trend seen with and without correction for null alleles (Table 4; Appendix S1, Table S6). Finally, a significant correlation between geographic distances and genetic distances (both, Nei and *F*_ST_) was found in the full data set (Mantel test, Table 5). These results suggest a strong genetic differentiation among the mainland Ecuador and Galapagos guava individuals.

**TABLE 3:** Results of the analysis of molecular variance (AMOVA) performed for (1) the full data set including guava populations from mainland Ecuador and the Galapagos, (2) the Galapagos populations only (Isabela, Santa Cruz, San Cristobal, and Floreana Islands), and (3) the mainland population only. Missing data were ignored for these AMOVA calculations

Source of variation	Mainland and Galapagos		Galapagos only		Mainland only	
	% of variation	*p*‐value	% of variation	*p*‐value	% of variation	*p*‐value
Between populations (Islands/Regions)	31.21	.001	14.89	.001	8.29	.001
Among samples within island/region	37.56	.001	43.87	.001	54.36	.001
Within samples	31.23	.001	41.24	.001	37.35	.001

**TABLE 4:** Pairwise *F*_ST_ values between all pairs of mainland Ecuador regions and Galapagos populations of guava

	NC	NH	NA	CC	CH	CA	SC	SH	SA	ISA	SCZ	SCY
NH	0.053											
NA	0.072	0.030										
CC	0.033	0.063	0.058									
CH	0.151	0.092	0.105	0.011								
CA	0.100	0.078	0.025	0.048	0.074							
SC	0.111	0.081	0.079	0.085	0.077	0.069						
SH	0.156	0.114	0.100	0.079	0.043	0.090	0.032					
SA	0.208	0.145	0.124	0.130	0.092	0.124	0.067	0.044				
ISA	0.594	0.561	0.556	0.526	0.487	0.540	0.498	0.502	0.545			
SCZ	0.481	0.455	0.466	0.420	0.404	0.438	0.418	0.431	0.455	0.121		
SCY	0.493	0.470	0.491	0.427	0.418	0.469	0.450	0.468	0.499	0.205	0.075	
FLO	0.635	0.545	0.524	0.518	0.471	0.533	0.462	0.450	0.509	0.070	0.200	0.344

Note

Mainland Ecuador regions are as follows: North Coast (NC), North Highlands (NH), North Amazon (NA), Central Coast (CC), Central Highlands (CH), Central Amazon (CA), South Coast (SC), South Highlands (SH), and South Amazon (SA). Galapagos populations are as follows: Isabela (ISA), Santa Cruz (SCZ), San Cristobal (SCY), and Floreana (FLO). Northern mainland regions in orange, Central in yellow, Southern in blue. Darker colors represent greater mean geographic distances between mainland regions on the North‐South axis

**TABLE 5:** Results of the Mantel test using the Spearman regression (10,000 permutations) for detecting a correlation between geographic and genetic (Nei's and *F*_ST_) distances in (1) the full data set including guava populations from mainland Ecuador and the Galapagos, (2) the Galapagos populations only (Isabela, Santa Cruz, San Cristobal, and Floreana Islands), and (3) the mainland population only

	Nei's distance		*F*_ST_	
	Rho	*p*‐value	Rho	*p*‐value
Mainland and Galapagos	0.905	<.001*	0.844	<.001*
Galapagos only	0.143	.500	0.543	.083
Mainland only	0.543	.002*	0.470	.008*

*p‐values showing a significant correlation between geographic and genetic distances.

### 3.4 Genetic structure in the native and introduced range of guava

Genetic differentiation was less prevalent within mainland Ecuador than in the Galapagos archipelago. For instance, the variation among islands in the Galapagos explained a relatively high percentage of the total diversity in the archipelago, while a lower percentage of the total variation in mainland Ecuador occurred among the different geographic groups (Table 3). Likewise, pairwise *F*_ST_ values (corrected and not corrected for null alleles) tended to be higher among the Galapagos populations than between the mainland regions (Wilcoxon test, *p* = .050 for uncorrected values, and *p* = .007 for corrected values) (Table 4).

**FIGURE 5:**
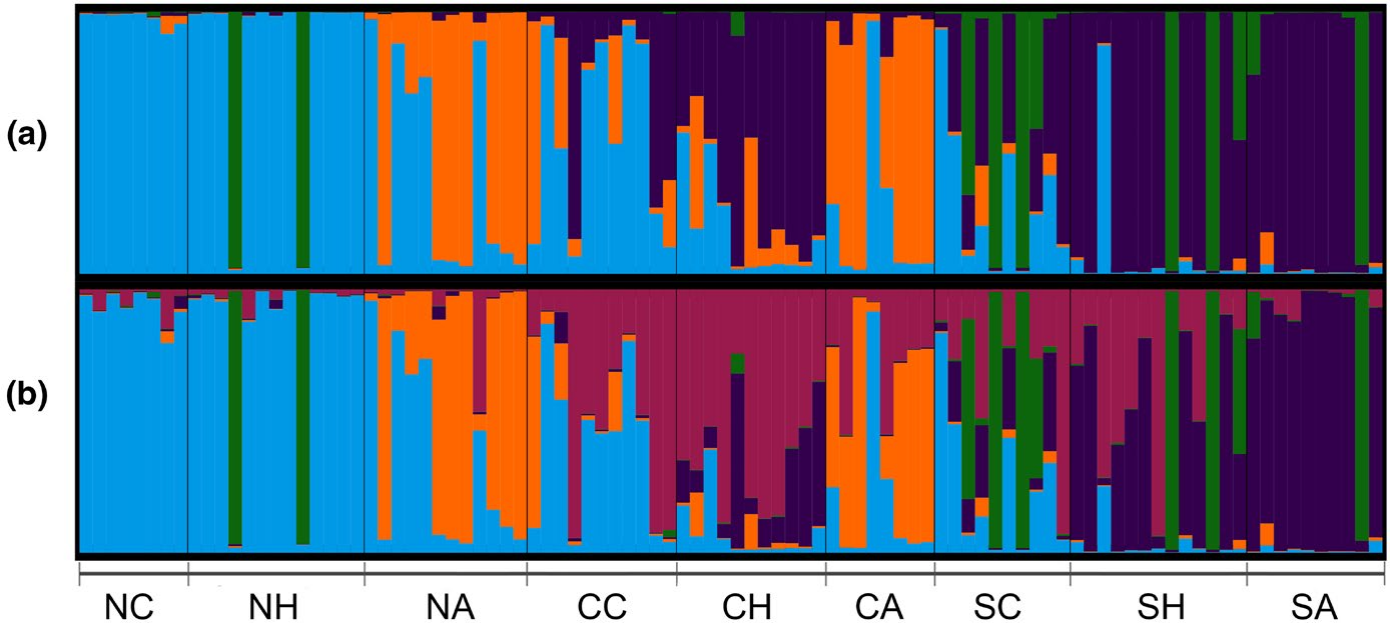
STRUCTURE plot (Bayesian analysis of population structure, admixture model) for the mainland Ecuador guava population. (a) *K* = 4 (best *K* value, Δ*K* = 39.92), (b) *K* = 5 (Δ*K* = 2.45). The values of *K* correspond to the number of genetic clusters (represented by different colors) in which are grouped the sampled individuals. The mainland population was divided *a priori* into 9 regions: North Coast (NC), North Highlands (NH), North Amazon (NA), Central Coast (CC), Central Highlands (CH), Central Amazon (CA), South Coast (SC), South Highlands (SH), and South Amazon (SA)

Isolation by distance was observed in mainland Ecuador, as both pairwise *F*_ST_ and Nei genetic distances were significantly correlated with pairwise geographic distances (Table 5). According to our STRUCTURE results, this isolation‐by‐distance process predominantly occurs along a north–south axis, with admixture in the central regions. Considering the optimum number of clusters (*K* = 4; Figure 5a; Appendix S1, Figure S2), we observe one lineage mainly present in northern Ecuador (predominantly in NC and NH), and in the CC region to a lesser extent (shown in cyan; hereinafter referred to as Northern/coastal lineage). The Southern lineage (shown in purple) was concentrated in the SH and SA regions, as well as in the CH region. A third lineage was found in the CA and NA Amazonian regions (shown in orange; hereinafter referred to as Amazonian lineage), and a fourth lineage (shown in green; hereinafter referred to as Dispersed lineage) was found to account for some individuals dispersed across mainland Ecuador. When assuming an additional cluster (*K* = 5; Figure 5b), a fifth lineage appears predominantly in the central regions (CC, CH, and CA), with some presence in the SC and SH regions as well (shown in dark red; hereinafter referred to as Central lineage). Considering the moderately high frequencies we found for null alleles in the mainland population (Appendix S1, Table S4) and its deviations from HWE, these clustering patterns from STRUCTURE should be interpreted carefully (Pritchard et al., 2000). Nevertheless, we do not think these results are in disagreement with other results that were corrected for null alleles, including the clustering shown in the *D*_CH_‐based NJ tree (Figure 4), and the corrected *F*_ST_ values (Appendix S1, Table S6) that tend to be lower among groups located at similar latitudes and nearby geographic locations.

The previously reported population structure for the Galapagos guava populations (Urquía et al., 2019) was confirmed in the present study, which also includes Floreana Island. Thus, the Galapagos guavas were clustered in two main groups (Figure 2b,c), one harboring Isabela and Floreana individuals (Isabela/Floreana lineage), and another containing San Cristobal individuals (San Cristobal lineage). The Santa Cruz population was represented as an admixture of the Isabela/Floreana and San Cristobal lineages, with more dominance from the latter (Figure 2b). Similarly, Santa Cruz individuals appear scattered across the San Cristobal and Isabela/Floreana clusters in the genetic distance‐based PCoA (Figure 2c). No correlation was found among genetic and geographic distances in the Galapagos guava populations (Table 5), ruling out isolation by distance as the explanation behind the observed population structure in the islands.

### 3.5 Putative origins of the Galapagos guava in mainland Ecuador

Different approaches were used to explore the origin of the Galapagos guava from mainland Ecuador. Pairwise *F*_ST_ values were not very useful for this purpose, since all estimates were considerably high and no distinct pattern was observed. Nevertheless, relatively lower values were observed between the Galapagos populations and the CH, CC, SC, and SH mainland groups (Table 4; Appendix S1, Table S6).

The NJ trees based on Nei distances (Figure 3) and *D*_CH_ corrected for null alleles (Figure 4) show that the Galapagos populations are clustered into a single group, reflecting its isolation from the mainland and suggesting perhaps a primary origin (or origins) of the insular populations. In accordance with the STRUCTURE results, the northern and southern mainland regions cluster according to a latitudinal range, with the NA and CA regions clustering together as well (Figures 3 and 4). The CH region, the southern regions (SC, SH, and SA), and to a lesser extent the CC region appear to be genetically the closest to the Galapagos populations in both trees (Figures 3 and 4). These results support the greater genetic proximity of individuals from these mainland regions to the guavas in the Galapagos Islands, and hence delimit this geographic area as a more likely origin.

Considering only the Galapagos data set, four high‐frequency (>0.05) private alleles were found in Isabela, the only insular population presenting unique alleles. However, we were unable to propose a putative origin for the Isabela guava population by locating these private alleles in the mainland, as these were distributed at high frequencies in several mainland regions indistinctly. The Nei and *D*_CH_ genetic distances show once again that the CH region presents the lowest genetic distances to all the four Galapagos populations, mirrored by the NJ tree topologies (Figures 3 and 4). Nonetheless, Nei distances between the San Cristobal population and the coastal mainland regions (NC, CC, and SC) tend to be lower compared with the other Galapagos populations (Figure 3). The null allele‐corrected *D*_CH_ distances reveal lower distances between Isabela and the SC and SH regions, something not observed for the San Cristobal population (Figure 4). Our STRUCTURE results (Figure 2b) showed that the San Cristobal lineage (shown in cyan) and the Isabela/Floreana lineage (shown in light green) both contribute to individuals in the CH region in approximately equal proportions (3.3% and 4.8%, respectively). However, each of these lineages does also contribute to different groups in the mainland regions. While the San Cristobal lineage contributes to the NC and CC regions (7.6% and 5.5% respectively), the Isabela/Floreana lineage contributes to the SC and SH regions (5.7% and 6.5%, respectively). This observation agrees with the patterns found for the Nei and *D*_CH_ genetic distances, suggesting that the southern regions could have contributed more of the genetic component in Isabela and Floreana, while the coastal regions had a greater input on the San Cristobal genetic diversity; all guavas in the Galapagos would have had an input from the CH region.

Our ABC analyses based on the mainland lineages defined by the STRUCTURE output (see Figure 5b and Section 3.4) consistently supported the scenario where the Isabela/Floreana lineage descends from the admixture of the Central and Southern mainland lineages (Table 6). This scenario had the highest posterior probability (PP) among those tested (none of the other scenarios were supported by a mean PP > 0.1). Despite the fact that only 68.2% of the tested summary statistics adequately fitted this scenario, the PCA checking (i.e., simulated data derived from the model matches our observed data within the PCA) validates this model (Appendix S1, Figure S3). According to this scenario, the Central and Southern lineages contributed equally to the Isabela/Floreana lineage, with 43.4% (95% CI = 2.4%–95.9%) coming from the former, and the rest from the latter (File S7). The extent of the contribution of each lineage remains uncertain given the wide CI. The contribution of the Central lineage at the upper bound would effectively nullify that of the Southern lineage and vice versa for the lower bound. Unfortunately, the origins of the San Cristobal lineage could not be reliably identified, since all the tested scenarios resulted in low PPs with overlapping confidence intervals. Nonetheless, a slightly higher PP was obtained for the scenario supporting an origin from the Northern/coastal and Central lineages (Table 6).

**TABLE 6:** Scenarios tested through ABC (DIYABC software) for locating the origin of the Galapagos guava lineages in mainland Ecuador

Galapagos lineage	Potential origins in tested ABC scenarios	Selected best scenario	PP + 95% CIs
Isabela/Floreana	Single origins: Northern/coastal lineage (cyan^a^), Amazonian lineage (orange), Central lineage (wine color), Southern lineage (purple), Dispersed lineage (green) Admixture between all possible pairs of lineages	Admixture: Central + Southern lineages	0.378 [0.307–0.449]
San Cristobal	Single origins: Northern/coastal lineage (cyan^a^), Amazonian lineage (orange), Central lineage (wine color), Southern lineage (purple), Dispersed lineage (green) Admixture between all possible pairs of lineages	Admixture: Northern/coastal + Central lineages	0.160 [0.120–0.200]

Note

Posterior probabilities (PPs) of the best‐supported scenario explaining our data, and of other scenarios overlapping the best one in its 95% CIs on each analysis, are shown as well.

Values in [ ] correspond to the 95% CI.

^a^ Refer to colors in Figure 5.

### 3.6 Colonization history of the Galapagos guava

The first stage of the ABC analyses to infer the colonization pathway of guava in the Galapagos Islands (see Section 2.2.8) supported the same scenarios reported previously (Urquía et al., 2019). The best‐supported scenario that was selected for the next stages of the ABC analysis suggests three phases: (a) an initial colonization of guava in San Cristobal from mainland Ecuador, (b) the introduction of the species in Isabela/Floreana from San Cristobal, and (c) the arrival of guava to Santa Cruz from San Cristobal (Scenario 4, PP = 0.460, 95% CI = 0.392–0.528; File S1). The second best scenario was similar but featured the Santa Cruz population as the admixture of the San Cristobal and the Isabela/Floreana populations (Scenario 7, PP = 0.252, 95% CI = 0.189–0.315; File S1). This scenario agrees with the STRUCTURE results (Figure 2b), emphasizing the greater contribution of the San Cristobal lineage to the Santa Cruz population compared with the Isabela/Floreana lineage; according to the posterior distributions (PDs) obtained, Isabela/Floreana only contributed 20.2% (95% CI = 1.4%–65.2%) to Santa Cruz population.

In the second stage of this ABC analysis, we found that Floreana Island was colonized by guava before Isabela, and in fact seeded the latter (Scenario 4, PP = 0.624, 95% CI = 0.564–0.683; File S2). This scenario was vastly supported in comparison with others, where the PP point estimates were always below 0.07; thus, all alternative scenarios (Scenarios 5–8) were discarded (File S2).

**FIGURE 6:**
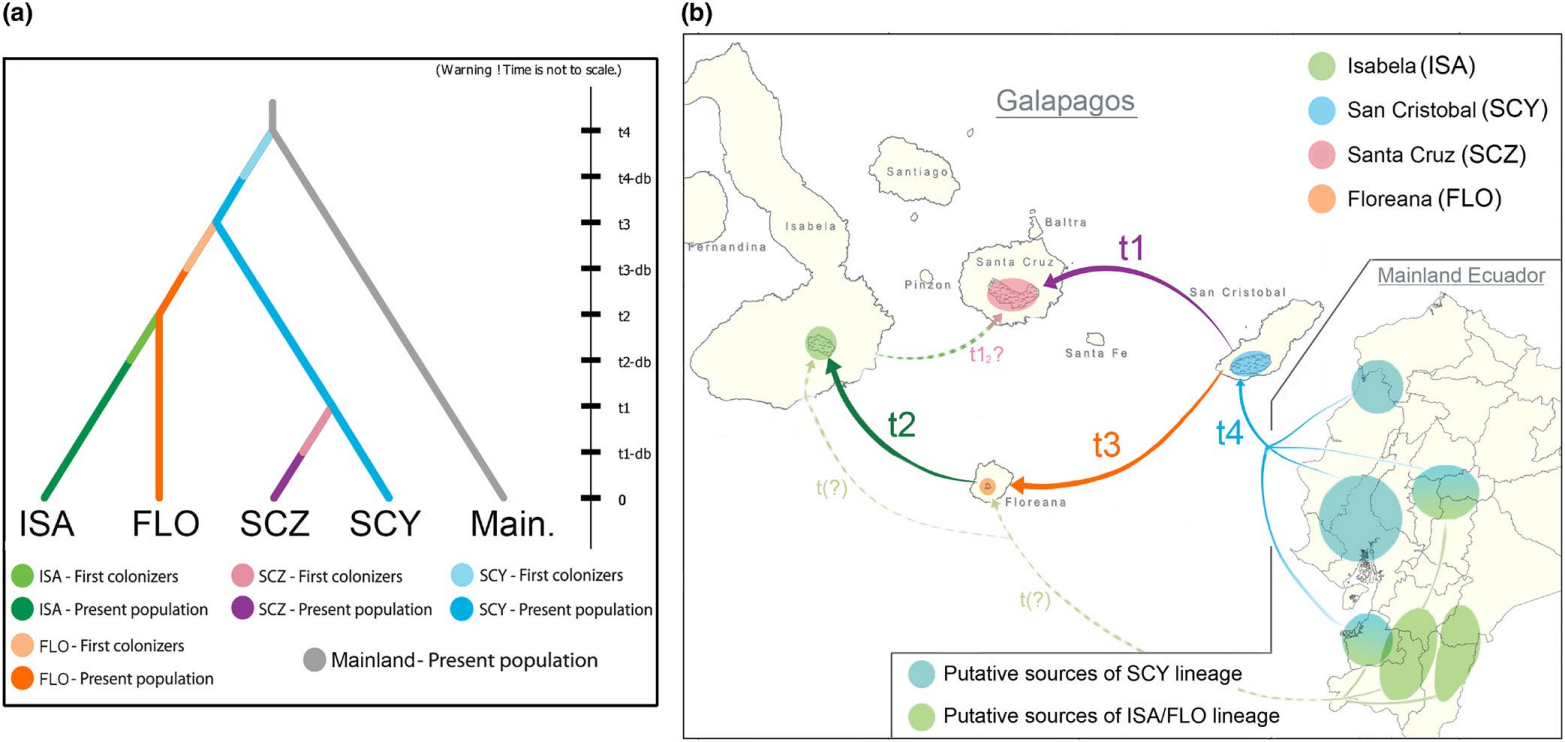
Final model proposed for the introduction and colonization history of guava in the Galapagos Islands, as inferred from the ABC analysis. (a) Diagram of the scenario generated by the DIYABC software. (b) Map illustrating the proposed colonization scenario: From the mainland (possibly from the CC, CH, NC, and/or SC regions), an initial introduction would have occurred in San Cristobal Island (t4), from where guava passed then to Floreana Island (t3), and from this one, later to Isabela (t2); finally, from San Cristobal, guava would have passed to Santa Cruz, the last island colonized by this invasive species (t1). Although not supported by the ABC final model, other analyses suggest an admixture of the Isabela and San Cristobal guavas in Santa Cruz (t1 & t1_2_), and a second independent introduction from the mainland (likely from the southern regions and/or CH) to Floreana and Isabela islands, explaining the origin of the Isabela/Floreana (ISA/FLO) lineage (t(?)). Note: Mainland Ecuador and Galapagos maps are not at scale in this figure

Based on the best scenarios selected from the first and second stages of the ABC analysis, we proposed a final scenario that addresses the colonization history of guava in the Galapagos Islands (Figure 6). First, individuals from a source population in mainland Ecuador would have been introduced into San Cristobal Island. From here, guava would have dispersed and grab a hold of Floreana, from where it moved to Isabela afterward. Finally, Santa Cruz was seeded from San Cristobal, becoming the last island to be colonized by the invasive species. According to the PDs of the parameters of this scenario, the introduction into Santa Cruz (t1; Figure 6) would have been recent, dating only 34 (95% CI = 5–164) guava generations ago. The introduction of guava to Isabela from Floreana (t2) preceded the Santa Cruz introduction and occurred 119 (95% CI = 27–446) generations ago. The introduction to Floreana from San Cristobal (t3) would have occurred 211 (95% CI = 59–750) generations ago. Note the CIs of all these estimations overlap considerably. However, the separation of the San Cristobal population from the mainland source (t4) was surprisingly dated at a much earlier time point, 1710 (95% CI = 932–1990) generations ago (File S8). Our final scenario had good statistical support according to model checks implemented in DIYABC, as it showed a good fit to the observed data (87.6% of the tested summary statistics) and was validated in the PCA model checking as well (Appendix S1, Figure S4).

The aforementioned estimates do not coincide with those obtained from the *migraine* MLEs (*D*_g_ *μ* values; Table 2). According to these, the founding event in San Cristobal was recent, overlapping with the Santa Cruz introduction (this last, estimated to have occurred recently through both methods). Likewise, the Isabela founding event appears to have occurred earlier than in Floreana, although these estimates show an important overlap. Based on a mutation rate *μ* = 3.5e−04, the founder effect in Isabela would have occurred 238 (95% CI = 76–3174) guava generations ago, 123 (95% CI = 0–3697) generations ago for Floreana, 51 (95% CI = 22–346) generations ago for Santa Cruz, and 19 (95% CI = 1–1560) generations ago for San Cristobal.

## 4 DISCUSSION

### 4.1 Genetic diversity loss in the invasive guava populations in Galapagos

The invasive populations of guava in the Galapagos Islands display a lower genetic diversity when compared not only to the Ecuadorian population studied here (Table 1) but also to other native guava populations (investigated using SSR markers) in Venezuela (*H*_E_ = 0.740; Aranguren et al., 2010) and Brazil (*H*_E_ = 0.678; da Costa & Santos, 2013). These observations in guava follow the general rule of a lower genetic diversity in the introduced range than in the native range, as consistently supported by multiple studies (e.g., Amsellem et al., 2000; Hagenblad et al., 2015; Pettenkofer et al., 2020; Xu et al., 2015). A genetic bottleneck caused by a typical founder effect or by population contractions in general would have given way to the lower genetic diversity found in the Galapagos guava population, as suggested by our analyses (Table 2). The idea of a bottleneck in the Galapagos guava is also supported by an observed allelic loss of >50% in comparison with the mainland population (Table 1), which is actually more prominent than the average allele loss observed in introduced plant species around the world (Hagenblad et al., 2015). We also found evidence of small founder populations of guava in all the studied islands in the Galapagos, consisting of no more than a hundred individuals in Isabela and even less than that for Santa Cruz and San Cristobal (considering a mutation rate of *μ* = 3.5e−04; Table 2). These small and likely unstable founder populations could have also been exposed to additional genetic diversity loss via genetic drift and inbreeding (Ellstrand & Elam, 1993; Hagenblad et al., 2015).

### 4.2 Differences among native and introduced populations of guava

According to our results, the guava populations in the Galapagos are genetically distinct from the populations in mainland Ecuador (see Figures 2, 3, 4). This genetic differentiation is not surprising, since the Galapagos guava gene pool would have been shaped by founder effects and genetic drift (Hagenblad et al., 2015; Husband & Barrett, 1991), the potential admixture of distinct mainland sources (Chun et al., 2009; Hagenblad et al., 2015; Lombaert et al., 2011; Shirk et al., 2014), and potentially the effects of natural selection on the newly colonized environment (Stuessy et al., 2014). Furthermore, the geographic distance that separates the Galapagos from the closest mainland (approximately 1000 km) also constitutes a formidable barrier to gene flow and migration between the insular and mainland populations. Given the role of humans in the history of the species in the archipelago, this restriction to gene flow might have been exacerbated within the last decades, following the ban on the transportation of guava from mainland Ecuador to the Galapagos (Bigue et al., 2012; Urquía et al., 2019).

We found an important level of gene flow across the mainland populations, especially among regions in the same latitudinal range. In contrast, gene flow was more restricted in the Galapagos archipelago (Tables 3 and 4; Appendix S1, Table S6). This is expected considering that the species possesses no natural adaptations to disperse naturally across the ocean separating the individual islands in the Galapagos archipelago (Blake et al., 2012; Heleno et al., 2013). This, however, does not mean that gene flow is completely absent among guava populations from different islands; some gene flow, likely mediated by humans, could still occur (Urquía et al., 2019) between islands that are geographically close as suggested by pairwise *F*_ST_ values (Table 4; Appendix S1, Table S6). Another usual difference among native and introduced plant populations is that inbreeding and selfing are more prevalent in the latter and is a consequence not only of small founding populations (Ellstrand & Elam, 1993; Frankham, 1997), but also of the effects of positive selection on selfing and clonal reproduction in plants that arrive to a novel habitat and need to increase their population size quickly to become sustainable in the newfound environment (Amsellem et al., 2000; Carlquist, 1974; Webb & Kelly, 1993). However, and contrary to these expectations, we found that the native mainland guava population presented similarly high levels of inbreeding and/or selfing (expressed by the *F*_IS_; Allard et al., 1968; Twyford et al., 2014) compared with the introduced Galapagos populations (Table 1). We had previously proposed that high levels of inbreeding and selfing were important for the success of guava as an invasive species in the Galapagos (Urquía et al., 2019). It is possible that higher selfing frequencies could be advantageous for the guava populations in the mainland as well, and in fact could be linked with their abundancy all over tropical America (Caraballo, 2001).

### 4.3 Putative origins of the Galapagos guava in mainland Ecuador

Our results consistently suggest that the CH are the most likely source of the guava populations found in Isabela, Floreana, Santa Cruz and San Cristobal. The Ecuadorian coast, particularly the CC region, also had a close link to the Galapagos guavas, especially with the populations from San Cristobal (Figures 2, 3, 4). Additionally, the guava populations from Isabela and Floreana were also related to individuals in southern Ecuador, particularly from the SH region (Table 6).

As we hypothesized, the prominent contribution of the CC and CH regions to the Galapagos guava populations fits with the historical record of the colonization of the archipelago and the precedence of its first settlers. The CH region in our study includes the provinces of Tungurahua and the northern portion of Azuay (Figure 1); the Galapagos Islands received numerous immigrants from Tungurahua during their early human settling, including indigenous people of the Salasaca culture, which have established communities in the archipelago (Granda & Chóez, 2013; Grenier, 2007; Wogan, 2009). Meanwhile, northern Azuay includes the areas around the city of Cuenca, which is the birthplace of Manuel J. Cobos; Cobos is one of the most famous settlers in the Galapagos Islands, who established a massive sugar cane plantation in San Cristobal Island (Hacienda El Progreso) and a dye processing company in Floreana (Astudillo, 2018; Lundh, 2004; Pérez, 2005). The contribution of the Ecuadorian coast to the Galapagos guava populations, particularly the CC which includes the city of Guayaquil, is also reasonable. Guayaquil hosts the most important port of Ecuador and serves as the main hub between the mainland and the Galapagos Islands. In the 19th and 20th centuries, almost everything and everyone intending to reach the Galapagos, including migrants, livestock, and agricultural products (which surely included guava fruits and seeds), had to make a stop in the port of Guayaquil. Likewise, several settlers and landowners in the Galapagos were originally from Guayaquil and its surrounding areas; in fact, Cobos himself brought a significant number of workers and crops from this region to San Cristobal (Latorre, 2002; Lundh, 2004). Moreover, Guayaquil and the CC in general were and still are an important trading post, receiving products from all over the country, especially from the central Ecuadorian Andes (i.e., the CH region); consequently, the scenarios of an admixture of the guavas from the CC and CH regions and a further arrival in the Galapagos as our results suggested are well supported. Furthermore, the strong link between the CC guavas with the Galapagos populations could have also been reinforced by the human migration coming from the province of Manabi (Granda & Chóez, 2013; Wogan, 2009). Finally, a contribution from the SH to the Isabela and Floreana populations is also in agreement with the Galapagos colonization history: The archipelago received several immigrants from this region (which includes the provinces of Loja and Azuay; Figure 1), historically affected by severe droughts that forced their people to migrate (Granda & Chóez, 2013). Note that the first Galapagos settlers mostly came from rural settings and moved to the archipelago looking to work in farm and agricultural activities. It is therefore very likely that guavas were brought by these immigrants along other domestic plants, especially considering that guava is a valued plant due to its fruit, wood, medicinal properties, and positive effects on the soil (Astudillo, 2018; Caraballo, 2001; Hollingsworth, 2013; Sitther et al., 2014). This would not be the first case of an invasive species following the path of human colonizers and settlers, genetic analyses suggest that the weed *Bromus tectorum* expanded its range from Europe to a global scale in a similar pattern as European explorers and settlers did (Novak & Mack, 2001).

Assigning potential mainland sources to the Galapagos guava populations through genetic markers presents some limitations. Firstly, it is difficult to define discrete groups in continuous space, particularly given that possible sampling gaps in the mainland could have hidden or confounded the actual source populations and their structure. This was especially evident given the difficulties we had positing a possible origin for the San Cristobal lineage. To this point, a second limitation is that we only sampled populations within mainland Ecuador, which necessarily places a strong assumption and rules out any origin(s) from other countries. Nevertheless, our sampling in mainland Ecuador covered a representative portion of the known guava populations in the country, significantly increasing the probability that the source populations of the Galapagos guava were included if they were originally in mainland Ecuador (Figure 1a). Likewise, we found no private alleles in the Galapagos populations with respect to the mainland populations (with the exception of 6 alleles at frequencies <0.005; Table 1), suggesting that the Galapagos guava gene pool is well represented in our mainland sample. Finally, even when the islands have been visited by people from different countries in Europe and America (Lundh, 2004), our results are in agreement with historical sources (Lundh, 2004, 2006; Strauch, 1935), and palynological (Restrepo et al., 2012) and archaeobotanical evidence (Charred seeds; Astudillo, 2018), pointing out that guava was not introduced in the Galapagos earlier than the late 19th to early 20th centuries. During this time, most of the settlers and visitors in the Galapagos came from Ecuador (Lundh, 2004; Pérez, 2005). Thus, Galapagos has been tightly linked to mainland Ecuador from the time when guava are believed to have arrived until the current times. In fact, many other introduced plants in the Galapagos, including the invasive raspberry *Rubus niveus*, are believed to have come from Ecuador (Lundh, 2006; Quinton et al., 2011). Nevertheless, we should not completely exclude a potential input from other countries in tropical America (e.g., Colombia, Mexico, Peru) also historically linked to the Galapagos Islands due to geographic proximity and/or human immigration (see Lundh, 2004).

### 4.4 Multiple mainland source populations contribute to the Galapagos populations

There has been extensive debate regarding the importance of neutral genetic diversity on the invasion success of introduced species (see Dlugosch & Parker, 2008; Fisher, 1930; Holderegger et al., 2006; Reed & Frankham, 2001). A widely accepted paradox states that some alien species in novel habitats maintain a high spreading capacity despite experimenting bottlenecks and losing genetic diversity (Frankham, 2005; Roman & Darling, 2007; Sakai et al., 2001). Guava itself is considered one of the worst plagues in the Galapagos Islands (Tye et al., 2007), covering up to 20% of the agricultural areas of San Cristobal, Santa Cruz, Isabela, and Floreana; this is a total of 4955 ha. of monospecific guava forest across the four islands (Laso et al., 2020). Guava also has spread into the protected areas of the Galapagos National Park, covering more than 10,300 ha (Trueman et al., 2014).

One potentially important feature that makes guava an effective invader in the Galapagos Islands is its gene pool. Our results suggest that the Galapagos guava populations harbor genetic diversity from different mainland regions and were able to keep a relatively varied gene pool, despite bottlenecks. Several studies (e.g., Hagenblad et al., 2015; Novak & Mack, 2005; Pettenkofer et al., 2020; Rosenthal et al., 2008) have pointed out the importance of multiple introductions and/or sources to overcome the constraints associated with bottlenecks and diversity loss in a novel habitat, not only by rising genetic variability but also by providing genotypes appropriate for a wide range of new niches and environmental conditions (Dlugosch & Parker, 2008; Lombaert et al., 2011; Maron et al., 2004; Miura, 2007; Shaik et al., 2016; Xu et al., 2015). In the case of guava in the Galapagos, we can infer an input from populations from diverse habitats in the mainland, including Andean valleys and foothills (CH and SH regions), low coastal regions and floodplains (NC, CC, and SC regions), and dry scrublands (CC and SH regions) (Ron, 2020). Therefore, the Galapagos populations would have been well equipped to face the fluctuating and diverse climatic conditions in the archipelago (Binggeli & Healey, 1998; Carvajal, 2016; Miura, 2007; Thompson et al., 2011).

### 4.5 The dispersal pathway of guava in the Galapagos Islands

As our final invasion model shows (Figure 6), San Cristobal seems to be the starting point of the guava invasion in the Galapagos Islands. This idea is supported by the contribution of the inferred San Cristobal genetic lineage in all the four islands to different degrees (Figure 2b). Moreover, this suggestion makes sense from a historical point of view as we predicted. San Cristobal was not the first settled island (by humans) in the archipelago, but the first large‐scale and permanent settlements in the Galapagos were founded here. One of these settlements was Hacienda El Progreso, established by Manuel J. Cobos, as early as 1868. Notably, the earliest physical evidence of guava found in San Cristobal (charred seeds underground) dates from the same time when Cobos started rising sugar cane in El Progreso (Astudillo, 2018). Furthermore, according to Lundh (2004), the earliest report of guava in the Galapagos dates around 1889–1890, with three individuals planted in Cobos' property in San Cristobal. As our *migraine* analysis suggests (Table 2), a small number of individuals (with a broad CI range though) triggered the invasion in San Cristobal. Therefore, it is likely that these first three trees reported by Lundh, together with some few more, were the initiators of this invasion event not only in San Cristobal but in all the Galapagos ulteriorly.

According to our proposed model (Figure 6), Floreana followed San Cristobal in the invasion trajectory. Floreana was the first island in the archipelago settled by humans during the early 19th century (Lundh, 2004, 2006). Consequently, as we hypothesized, an early invasion of guava in this island also agrees with the history of the Galapagos. Likewise, during most of the 19th century, San Cristobal and Floreana were the only islands in the Galapagos harboring permanent human settlements and sustained activities as the trading and exchange of products and workers among them; it is therefore very likely that guava reached Floreana during this period (Lundh, 2004; Urquía et al., 2019). From here, guava was introduced to Isabela, the third island in being formally colonized by humans. A settler called Antonio Gil could have played a role in this event, since he took cattle (and perhaps guava) from Floreana (where he lived for four years) to Isabela, where he raised a big farm in 1897 (Latorre, 1997; Lundh, 2004). Note that the emergence of the Isabela guava population from Floreana not only agrees with our ABC model but also with the close genetic similarity we found between the populations of these two islands, especially if we considered that they belong to a single genetic lineage as suggested by the STRUCTURE analysis (Figure 2b).

Santa Cruz was the last island to be invaded by guava according to our proposed model (Figure 6), concurrent with the fact that this island was the last to be settled by humans around 100 years ago, and consequent with our hypothesis of an importation route of guava in the Galapagos compatible with the colonization history of the archipelago. Although the results from our STRUCTURE analysis (Figure 2b) and one of the supported scenarios in the first stage of our ABC analysis (Scenario 7, File S1) suggest that the guava population from Santa Cruz is the result from an admixture of the San Cristobal and Isabela/Floreana lineages, this is no longer supported in our final ABC model. This last rather depicts a scenario where the Santa Cruz population direct descents from San Cristobal (Figure 6); such scenario is also supported by the same STRUCTURE plot, the NJ trees (Figures 3 and 4), and the PDs of Scenario 7 (see Section 3.6), as they show a disproportionally greater contribution of the San Cristobal genetic pool in the Santa Cruz population. Workers from Cobos that originally lived in San Cristobal went to Santa Cruz during the late 19th‐early 20th centuries, and Cobos himself established several small farms there. Likewise, there was a constant trading between these two islands shortly after the settlement of Santa Cruz between the 1920s and 1930s (Lundh, 2004). Thus, guava could have arrived from San Cristobal to Santa Cruz around this time. A significant founding effect with a small founding population was inferred for the Santa Cruz guava population suggesting the demographic expansion started with a few individuals (Table 2). However, according to our estimates, a significant population growth was also detected after the founder event that led the Santa Cruz guava population to become the largest in the Galapagos (Table 2). This demographic growth in Santa Cruz could correspond to the time when the invasion of guava indeed became a serious problem in the island around the 1950s (Lundh, 2006). Interestingly, this lag period lasting approximately two decades between the supposed arrival of guava in Santa Cruz and its actual spread as an invasive species agrees with the proposal of several authors that the occurrence of such a lag period accounts for the time when successful invaders gather more genetic diversity before spreading (Ellstrand & Schierenbeck, 2006; Mack et al., 2000).

Our final colonization model, together with the tight clustering of the Galapagos populations based on genetic distances (Figures 3 and 4), is compatible with a common origin and a single main introduction of guava from mainland Ecuador to the Galapagos Islands, starting in San Cristobal. Thus, even if our results support multiple mainland sources for the Galapagos guava, it seems that all these colonizing plants with distinct provenances were taken to the islands in a single bout, or at least in several events but in rapid succession within a small time frame. We recognize that the presence of high‐frequency private alleles in the Isabela population and the existence of a second lineage dominating in Isabela and Floreana are hard to explain under a single‐introduction scenario. Therefore, we propose that a second minor introduction from the mainland to Isabela and Floreana (very likely from south Ecuador), occurred in rapid succession, parallel to the main introduction and went undetected by our ABC analyses (Figure 6b, t(?)). This would explain the contribution of the Southern mainland lineage to the Isabela/Floreana lineage as seen from the ABC (Table 6) and in other results (see Section 3.5), and could be linked with the larger inferred founding populations (2*Nμ* founder) and less severe genetic bottlenecks (*N*‐ratio) of the Isabela and Floreana populations compared with that observed in San Cristobal (Table 2).

The final colonization model we present had a good statistical support, and agrees with our other results and with historical sources as well. However, this model has important limitations. Firstly, as suggested above, our model failed in explaining the distinction of the Isabela/Floreana lineage, especially considering it does not support a second introduction of guava into these two islands. Nevertheless, there could be alternative explanations for the presence of a different gene pool in Isabela and Floreana, which include isolation, founder effects, and genetic drift (Hagenblad et al., 2015; Lombaert et al., 2011; Shirk et al., 2014). A second gap our final model may have is related to population divergence time estimations, since it is not clear when guava arrived to each island, and does not always agree with historical sources (Lundh, 2004, 2006). The same is also true for the *migraine* founding event date estimates (employing the *D*_g_ *μ* values as a proxy for this) that indeed contradict in some cases the ABC estimations. This is especially evident for the San Cristobal case, where according to the ABC, it diverged from the mainland population thousands of generations ago (t4, Figure 6; File S8), while contradictorily according to *migraine*, the founding event in this island occurred more recently than in any other Galapagos population. The lower limits of the CIs of the *migraine* estimates for the founder event in Isabela (Table 2) and of the ABC divergence time of Floreana (t3, Figure 6) and Isabela (t2, Figure 6) sound more compatible with our historical sources. Nevertheless, dating more than a hundred generations ago, the point estimates of these parameters still overdate what we expected (especially considering that 2 years would be the minimum bound for the duration of a generation in guava; Orwa et al., 2009; Urquía et al., 2019). The estimations for Santa Cruz were the only ones that agreed with those expected, as according to the ABC (t1, Figure 6; File S2) and *migraine* MLEs (Table 2), this island's population could have appeared recently, 34 (a minimum of ~68 years), or 51 generations ago (a minimum of ~102 years). In conclusion, all these time estimates should be taken carefully. We need to consider that SSR mutation models (i.e., SMM) are not optimized molecular clocks compared with nucleotide mutation models (such as the ones used for SNP analyses). This could be addressed by experimentally characterizing and modeling the markers for their use under a coalescent framework, but this is not always feasible (Sun et al., 2009). Additionally, the stepwise mutation model (SMM) used over more highly parametrized alternatives such as the generalized stepwise model (GSM) represents a lower computational burden, appropriate for managing complex scenarios such as the ones explored here, but makes several simplifications and assumptions that can affect the accuracy of estimations (Calabrese et al., 2001; Leblois et al., 2014; Sun et al., 2009). Another issue we had is that the CIs we got for time estimates are large; hence, it was hard to say accurately and confidently when did each guava population appeared or diverged from another based on our results. Finally, it should be noted that we did not calculate directly the arrival date of guava in a particular island; we just used the ABC and *migraine* estimates as a proxy for this. Thus, in the mentioned case of San Cristobal for instance, the t4 event in our ABC final model (see Figure 6) may not be directly showing the introduction of guava in this island, but the divergence of a novel gene pool (still in the mainland), that hundreds of generations later was taken to San Cristobal (see Urquía et al., 2019, or a similar case in Pettenkofer et al., 2020). Perhaps the inaccuracy of the time estimates that conflict between the migraine and ABC results (both presenting wide CIs) could be overcome by broadening our sampling in the potential source population, the mainland. Despite our considerable sampling effort, which covered all the territory of mainland Ecuador, gaps in the sampling are unavoidable. It is likely we could have missed genetically informative populations, which would have better fit a more comprehensive model for the invasion process. Nevertheless, the task of sampling source populations precisely is also become difficult by the notable gene flow within the mainland population. Under these circumstances, defining discrete regions and populations within a continuous space lacking a strong population structure is quite a challenge. In addition, stochastic demographic events and changes occurred in this population are also relevant to consider (Lombaert et al., 2011; Ward et al., 2008).

## 5 CONCLUSIONS

Our results highlight the severity of the invasive process of guava in the Galapagos islands given the observed genetic diversity and history of is the populations in several islands. Despite the relatively lower genetic diversity of the insular guava populations compared with their mainland counterparts, the contribution of multiple sources in the mainland to the invading population's gene pool could have contributed to a high adaptability and success when invading the archipelago (Hughes et al., 2008; Xu et al., 2015). Thus, we reinforce the importance of genetic research complemented by independent data sources, such as historical records (Astudillo, 2018), to aid in gathering key information that explains the features of an invasion process. Such features include the invasive species' adaptability and its spreading success, and the aggressiveness of the invasion and the factors and events driving it. In particular, as our results supported, the immigration, colonization, and activities of humans in the past could have been important factors that help to explain the arrival of guava in the Galapagos Islands and its spread to at least four islands of the archipelago.

Information as the one presented in this study can be a first crucial milestone in developing a route map to face the guava problem in the Galapagos Islands.

## CONFLICT OF INTEREST

The authors declare that they have no known competing financial interests or personal relationships that could have appeared to influence the work reported in this paper.

## AUTHOR CONTRIBUTIONS

**Diego Urquía**: Data curation; formal analysis; investigation; methodology; software; validation; writing—original draft preparation; writing—review and editing. **Bernardo Gutierrez**: Formal analysis; software; validation; writing—original draft preparation; writing—review and editing. **Gabriela Pozo**: Formal analysis; writing—original draft preparation; writing—review and editing. **Maria Jose Pozo**: Formal analysis, writing—original draft preparation; writing—review and editing. **Maria de Lourdes Torres**: Conceptualization; funding acquisition; project administration; supervision; resources; investigation; visualization; writing—review and editing.

### OPEN RESEARCH BADGES

This article has earned an Open Data, for making publicly available the digitally‐shareable data necessary to reproduce the reported results. The data is available at https://doi.org/10.5061/dryad.f4qrfj6wv.

## Supporting information

Supplementary Material

Supplementary Material

Supplementary Material

Supplementary Material

Supplementary Material

Supplementary Material

Supplementary Material

Supplementary Material

Supplementary Material

## Data Availability

Sampling locations and microsatellite genotypes are deposited on Dryad https://doi.org/10.5061/dryad.f4qrfj6wv.
