# Supplementary material for: Origin and dispersion pathways of guava in the Galapagos Islands inferred through genetics and historical records
Source: Ecol Evol. 2021 Oct 4;11(21):15111–31. doi: 10.1002/ece3.8193 (PMC8571588; doi:10.1002/ece3.8193)
Supplement: Supplementary file 1 — Supplementary Material [file ECE3-11-15111-s001.pdf]

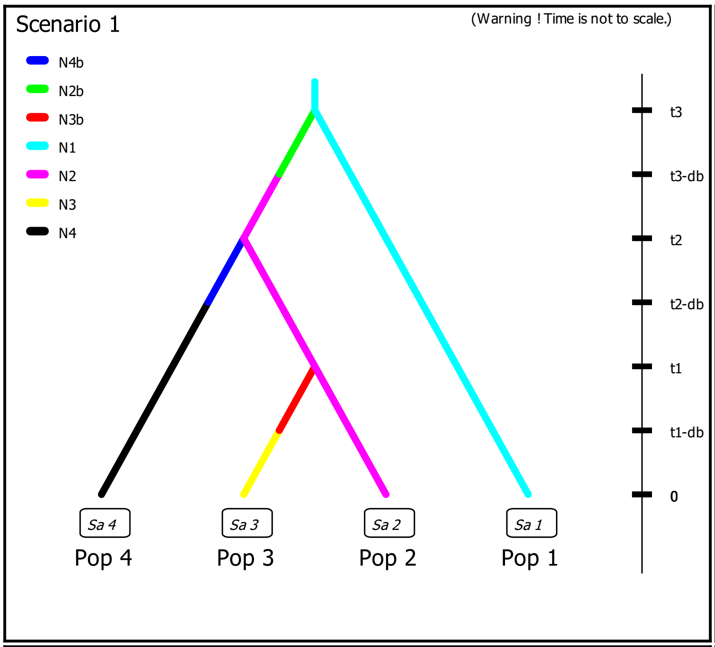
Scenario 1
(Warning ! Time is not to scale.)
N4b
N2b
N3b
N1
N2
N3
N4
t3
t3-db
t2
t2-db
t1
t1-db
0
Sa 4
Sa 3
Sa 2
Sa 1
Pop 4
Pop 3
Pop 2
Pop 1

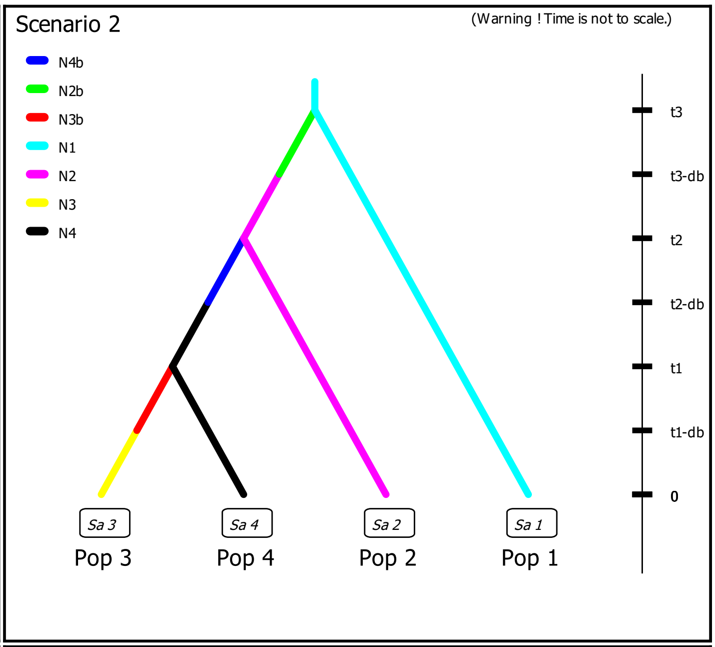
Scenario 2
(Warning ! Time is not to scale.)
N4b
N2b
N3b
N1
N2
N3
N4
t3
t3-db
t2
t2-db
t1
t1-db
0
Sa 3
Sa 4
Sa 2
Sa 1
Pop 3
Pop 4
Pop 2
Pop 1

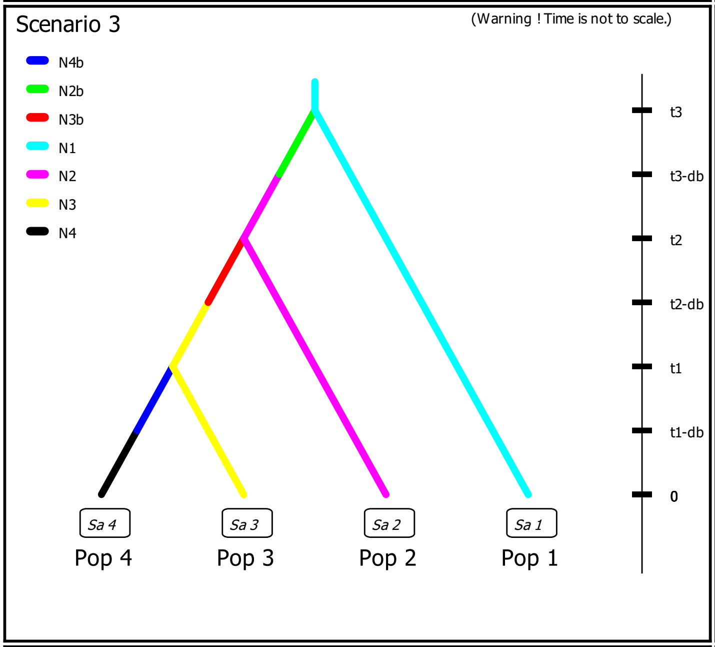
Scenario 3
(Warning ! Time is not to scale.)
N4b
N2b
N3b
N1
N2
N3
N4
t3
t3-db
t2
t2-db
t1
t1-db
0
Sa 4
Sa 3
Sa 2
Sa 1
Pop 4
Pop 3
Pop 2
Pop 1

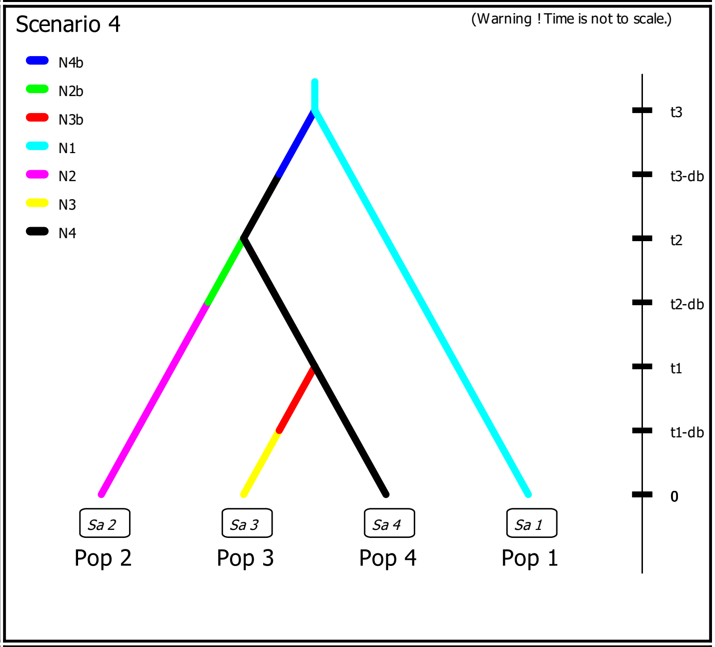
Scenario 4
(Warning ! Time is not to scale.)
N4b
N2b
N3b
N1
N2
N3
N4
t3
t3-db
t2
t2-db
t1
t1-db
0
Sa 2
Sa 3
Sa 4
Sa 1
Pop 2
Pop 3
Pop 4
Pop 1

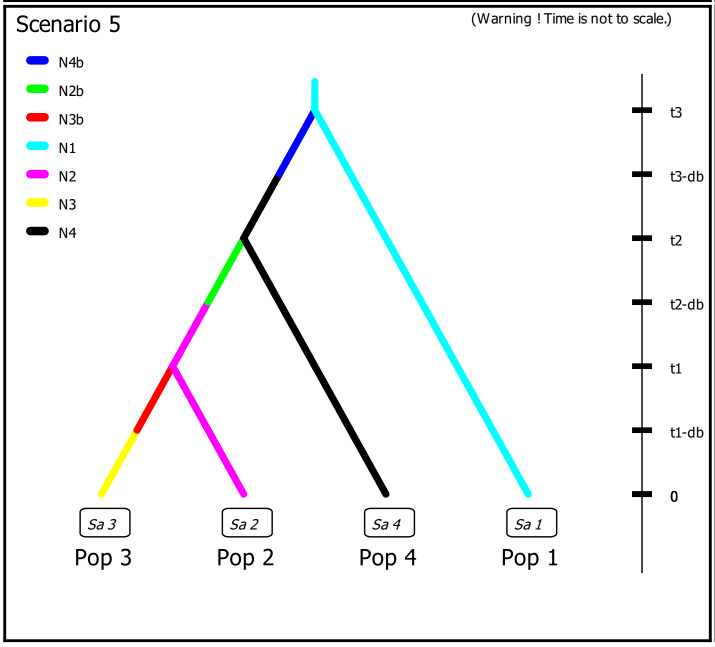
Scenario 5
(Warning ! Time is not to scale.)
N4b
N2b
N3b
N1
N2
N3
N4
t3
t3-db
t2
t2-db
t1
t1-db
0
Sa 3
Sa 2
Sa 4
Sa 1
Pop 3
Pop 2
Pop 4
Pop 1

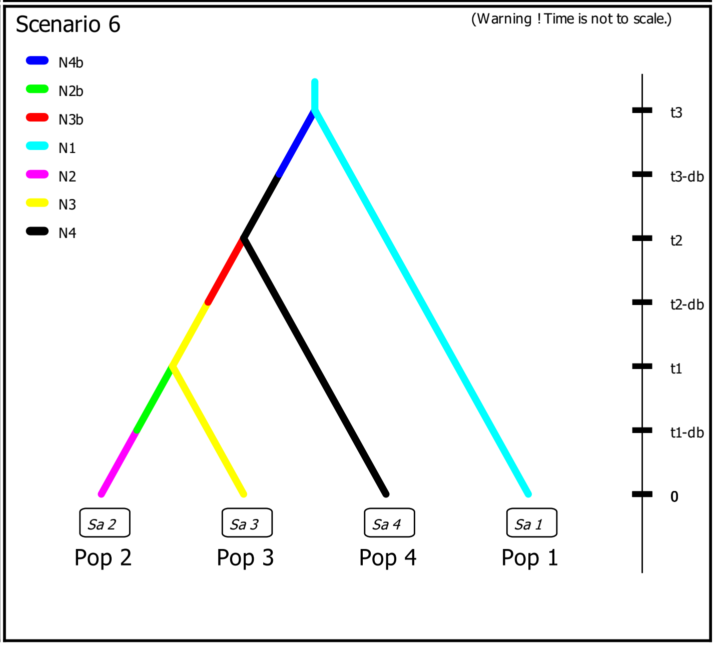
Scenario 6
(Warning ! Time is not to scale.)
N4b
N2b
N3b
N1
N2
N3
N4
t3
t3-db
t2
t2-db
t1
t1-db
0
Sa 2
Sa 3
Sa 4
Sa 1
Pop 2
Pop 3
Pop 4
Pop 1

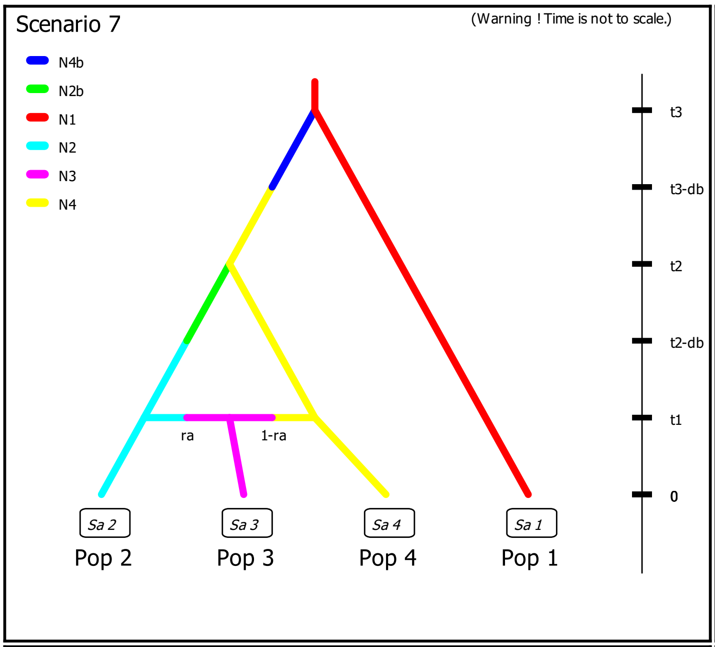
Scenario 7
(Warning ! Time is not to scale.)
N4b
N2b
N1
N2
N3
N4
ra
1-ra
t3
t3-db
t2
t2-db
t1
0
Sa 2
Sa 3
Sa 4
Sa 1
Pop 2
Pop 3
Pop 4
Pop 1

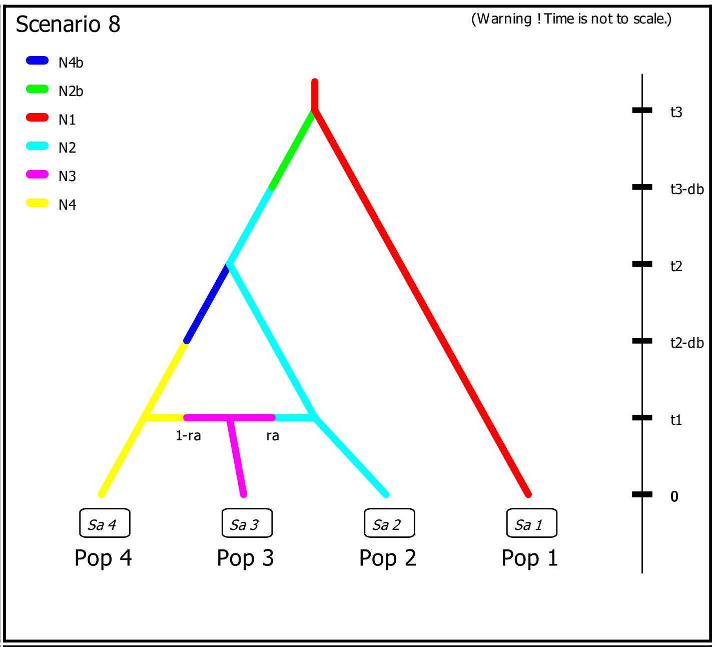
Scenario 8
(Warning ! Time is not to scale.)
N4b
N2b
N1
N2
N3
N4
1-ra
ra
t3
t3-db
t2
t2-db
t1
0
Sa 4
Sa 3
Sa 2
Sa 1
Pop 4
Pop 3
Pop 2
Pop 1

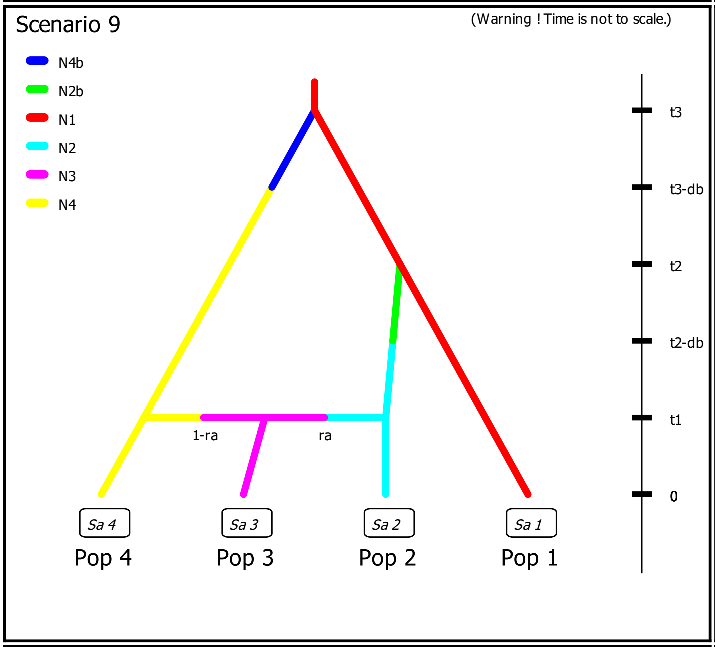
Scenario 9
(Warning ! Time is not to scale.)
N4b
N2b
N1
N2
N3
N4
1-ra
ra
t3
t3-db
t2
t2-db
t1
0
Sa 4
Sa 3
Sa 2
Sa 1
Pop 4
Pop 3
Pop 2
Pop 1

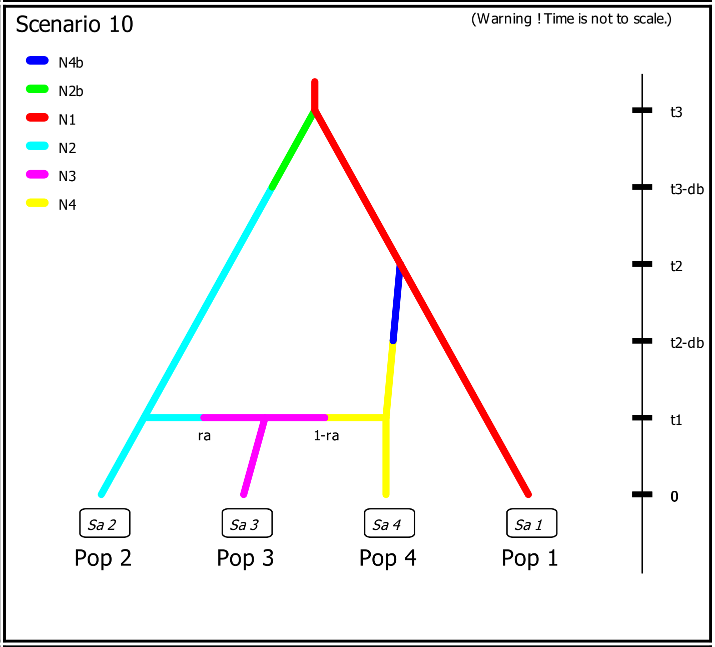
Scenario 10
(Warning ! Time is not to scale.)
N4b
N2b
N1
N2
N3
N4
ra
1-ra
t3
t3-db
t2
t2-db
t1
0
Sa 2
Sa 3
Sa 4
Sa 1
Pop 2
Pop 3
Pop 4
Pop 1

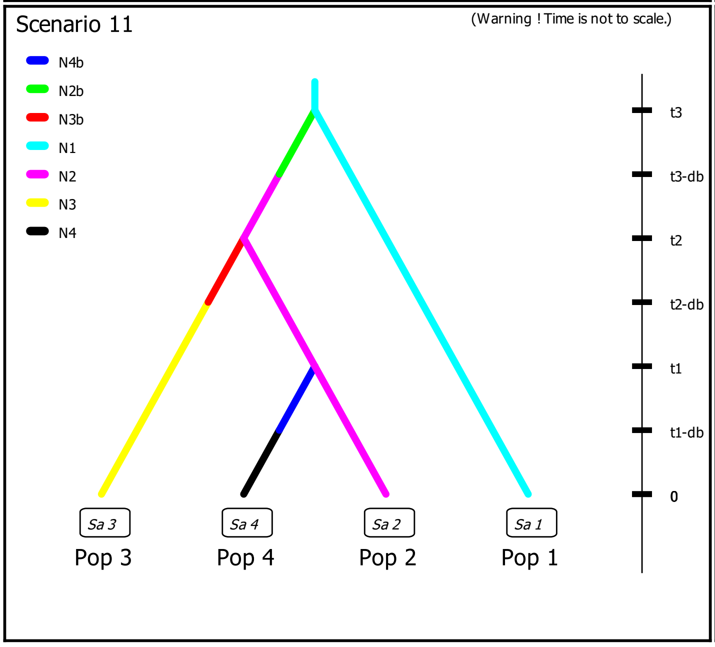
Scenario 11
(Warning ! Time is not to scale.)
N4b
N2b
N3b
N1
N2
N3
N4
t3
t3-db
t2
t2-db
t1
t1-db
0
Sa 3
Sa 4
Sa 2
Sa 1
Pop 3
Pop 4
Pop 2
Pop 1

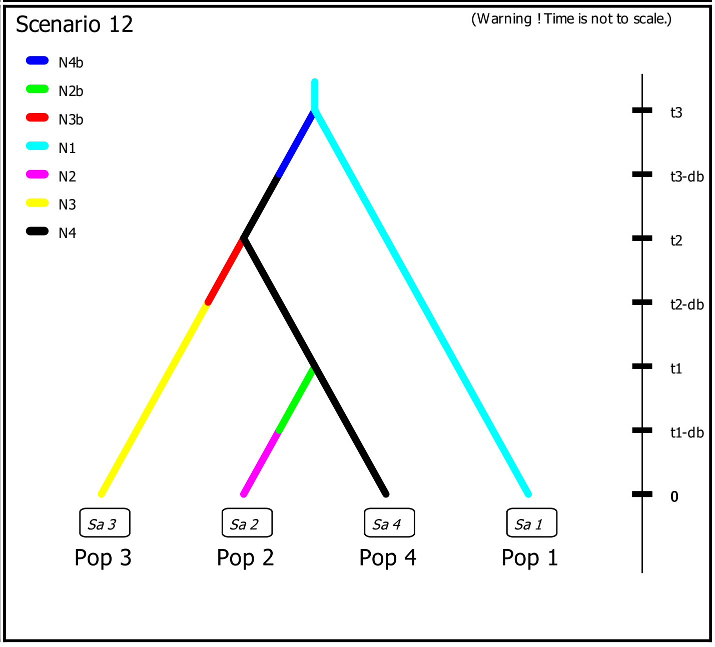
Scenario 12
(Warning ! Time is not to scale.)
N4b
N2b
N3b
N1
N2
N3
N4
t3
t3-db
t2
t2-db
t1
t1-db
0
Sa 3
Sa 2
Sa 4
Sa 1
Pop 3
Pop 2
Pop 4
Pop 1

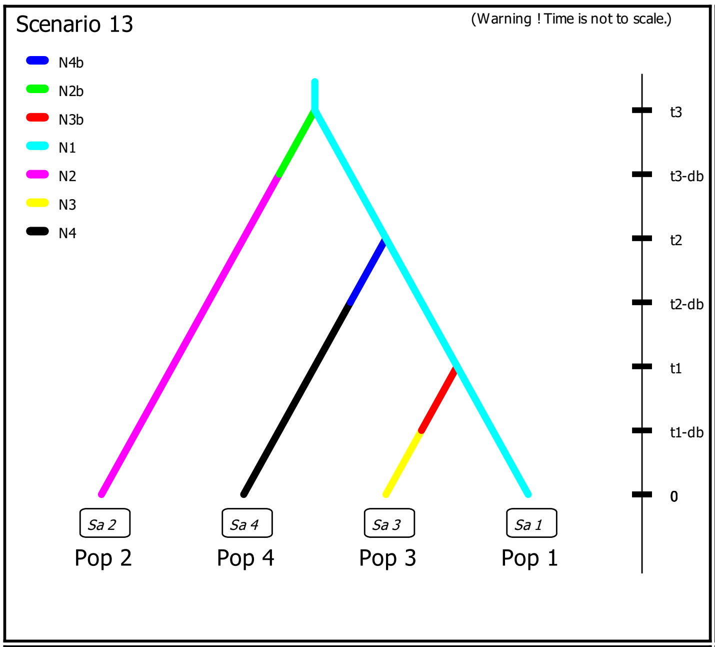
Scenario 13
(Warning ! Time is not to scale.)
N4b
N2b
N3b
N1
N2
N3
N4
t3
t3-db
t2
t2-db
t1
t1-db
0
Sa 2
Sa 4
Sa 3
Sa 1
Pop 2
Pop 4
Pop 3
Pop 1

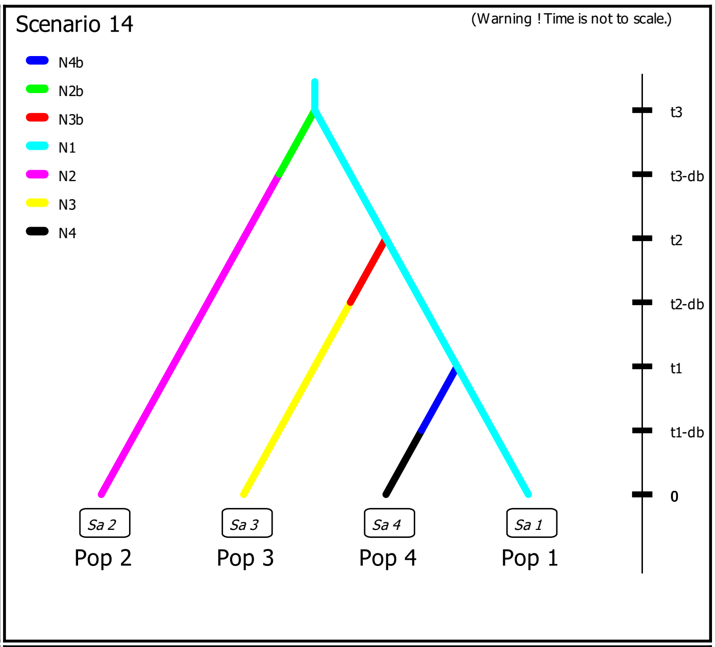
Scenario 14
(Warning ! Time is not to scale.)
N4b
N2b
N3b
N1
N2
N3
N4
t3
t3-db
t2
t2-db
t1
t1-db
0
Sa 2
Sa 3
Sa 4
Sa 1
Pop 2
Pop 3
Pop 4
Pop 1

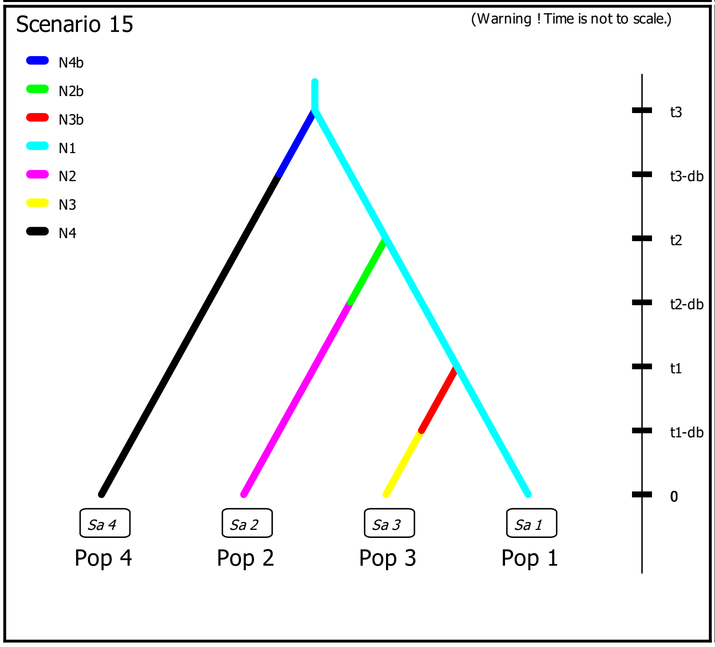
Scenario 15
(Warning ! Time is not to scale.)
N4b
N2b
N3b
N1
N2
N3
N4
t3
t3-db
t2
t2-db
t1
t1-db
0
Sa 4
Sa 2
Sa 3
Sa 1
Pop 4
Pop 2
Pop 3
Pop 1

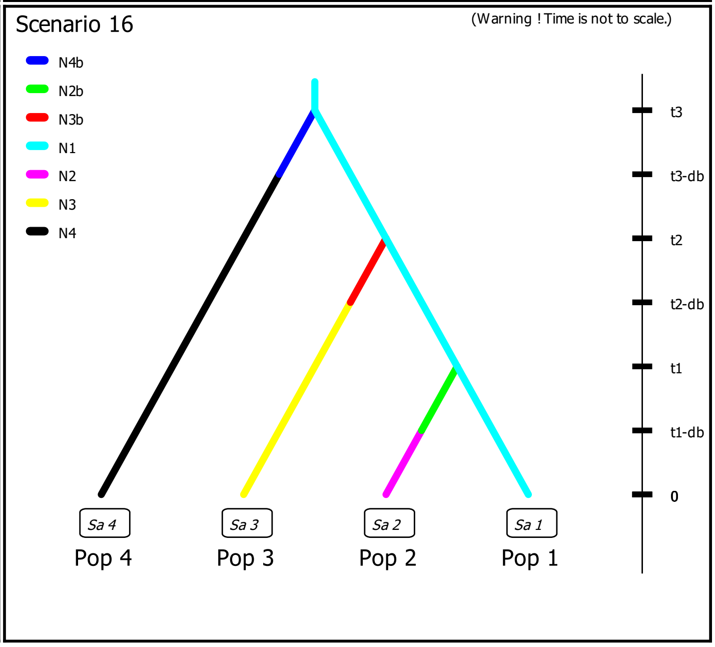
Scenario 16
(Warning ! Time is not to scale.)
N4b
N2b
N3b
N1
N2
N3
N4
t3
t3-db
t2
t2-db
t1
t1-db
0
Sa 4
Sa 3
Sa 2
Sa 1
Pop 4
Pop 3
Pop 2
Pop 1
