# Supplementary material for: Origin and dispersion pathways of guava in the Galapagos Islands inferred through genetics and historical records
Source: Ecol Evol. 2021 Oct 4;11(21):15111–31. doi: 10.1002/ece3.8193 (PMC8571588; doi:10.1002/ece3.8193)
Supplement: Supplementary file 2 — Supplementary Material [file ECE3-11-15111-s004.pdf]

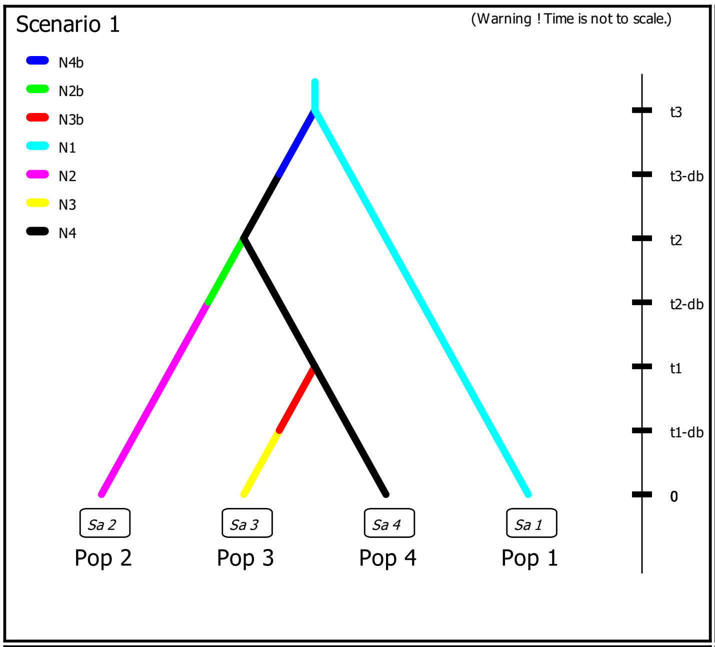

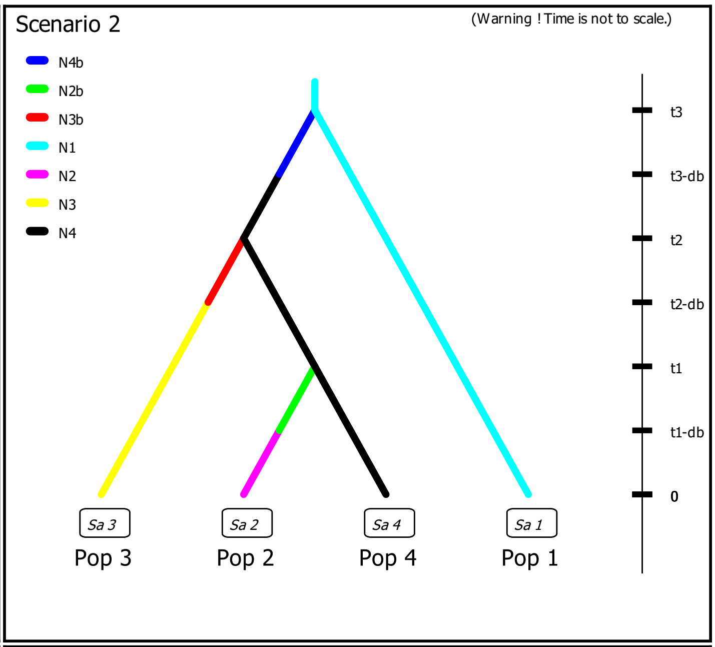

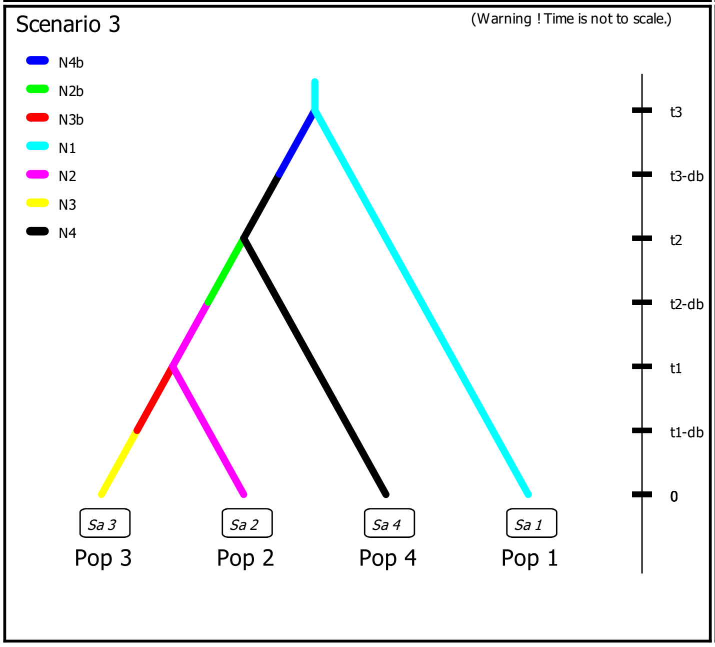

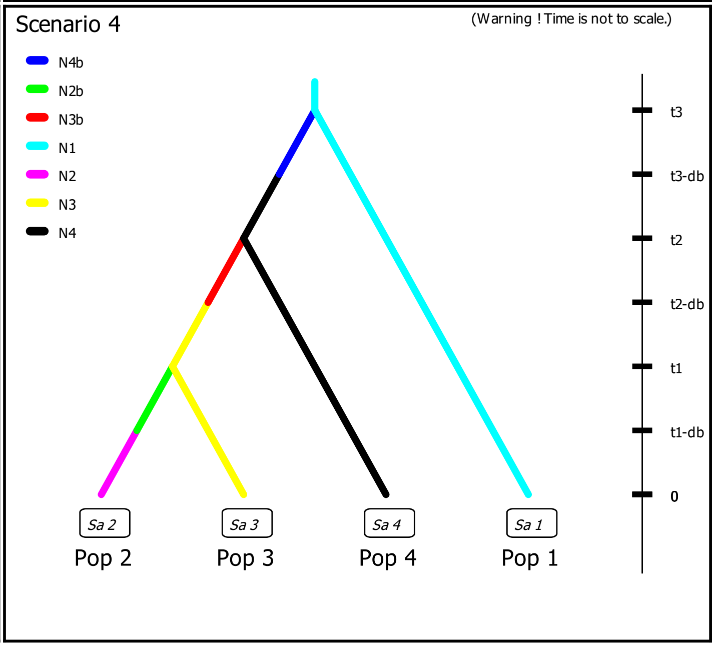

Scenario 5

N1 N2 N3 N4
0 sample 1
0 sample 2
0 sample 3
0 sample 4
t1 split 3 4 1 ra
t2 split 2 4 1 ra
t3-db VarNe 4 N4b
t3 merge 1 4

Scenario 6

N1 N2 N3 N4
0 sample 1
0 sample 2
0 sample 3
0 sample 4
t1 split 2 4 1 ra
t2 split 3 4 1 ra
t3-db VarNe 4 N4b
t3 merge 1 4

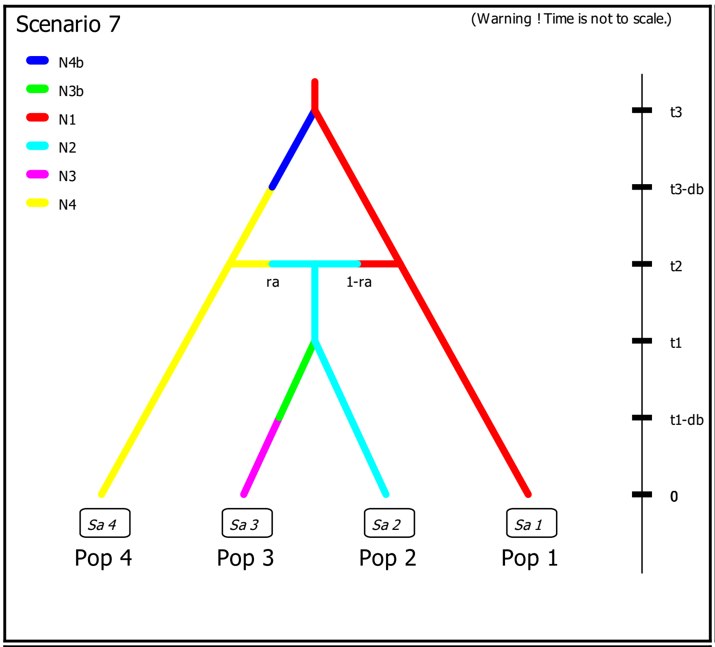
Scenario 7
(Warning ! Time is not to scale.)
N4b
N3b
N1
N2
N3
N4
t3
t3-db
t2
t1
t1-db
0
ra
1-ra
Sa 4
Sa 3
Sa 2
Sa 1
Pop 4
Pop 3
Pop 2
Pop 1

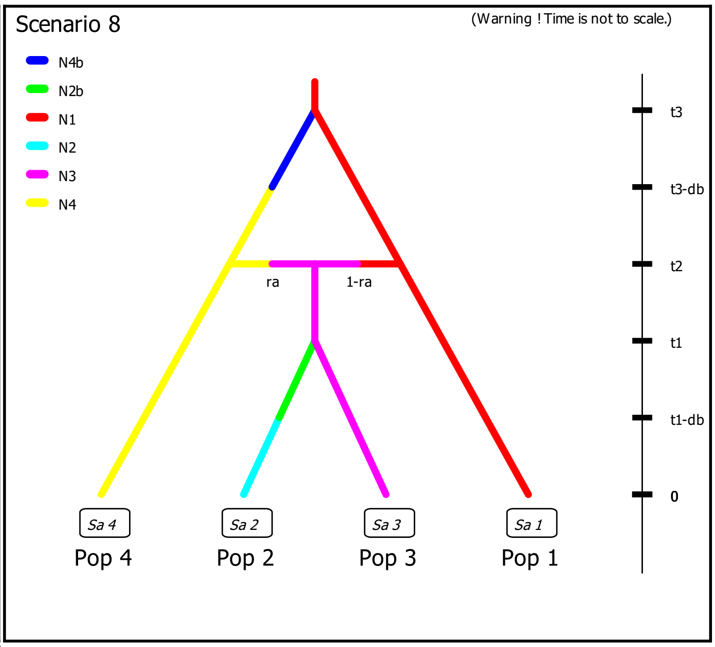
Scenario 8
(Warning ! Time is not to scale.)
N4b
N2b
N1
N2
N3
N4
t3
t3-db
t2
t1
t1-db
0
ra
1-ra
Sa 4
Sa 2
Sa 3
Sa 1
Pop 4
Pop 2
Pop 3
Pop 1

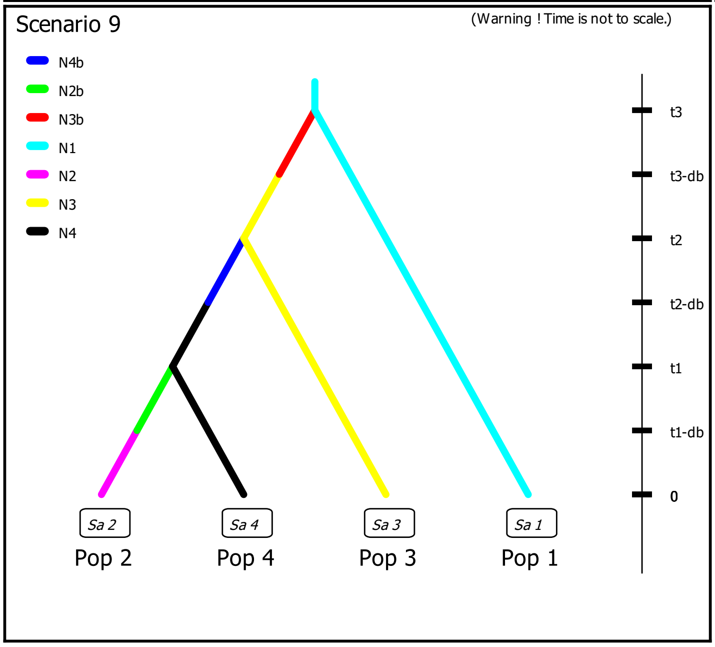
Scenario 9
(Warning ! Time is not to scale.)
N4b
N2b
N3b
N1
N2
N3
N4
t3
t3-db
t2
t2-db
t1
t1-db
0
Sa 2
Sa 4
Sa 3
Sa 1
Pop 2
Pop 4
Pop 3
Pop 1
