# Supplementary material for: Origin and dispersion pathways of guava in the Galapagos Islands inferred through genetics and historical records
Source: Ecol Evol. 2021 Oct 4;11(21):15111–31. doi: 10.1002/ece3.8193 (PMC8571588; doi:10.1002/ece3.8193)
Supplement: Supplementary file 3 — Supplementary Material [file ECE3-11-15111-s003.pdf]

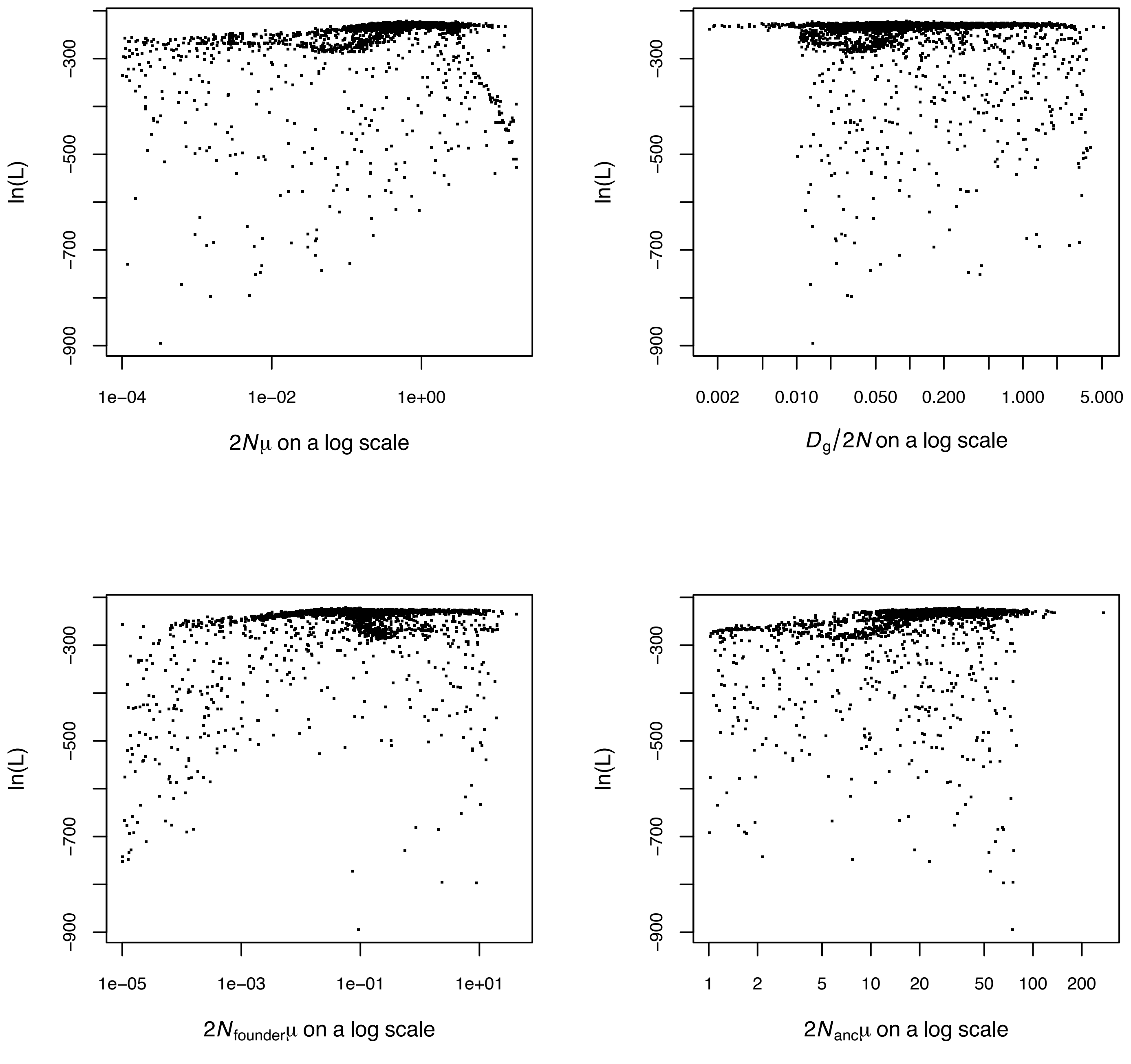
ln(L)
−300
−500
−700
−900
1e−04
1e−02
1e+00
$2N\mu$ on a log scale
ln(L)
−300
−500
−700
−900
0.002
0.010
0.050
0.200
1.000
5.000
$D_g/2N$ on a log scale
ln(L)
−300
−500
−700
−900
1e−05
1e−03
1e−01
1e+01
$2N_{founder}\mu$ on a log scale
ln(L)
−300
−500
−700
−900
1
2
5
10
20
50
100
200
$2N_{anc}\mu$ on a log scale

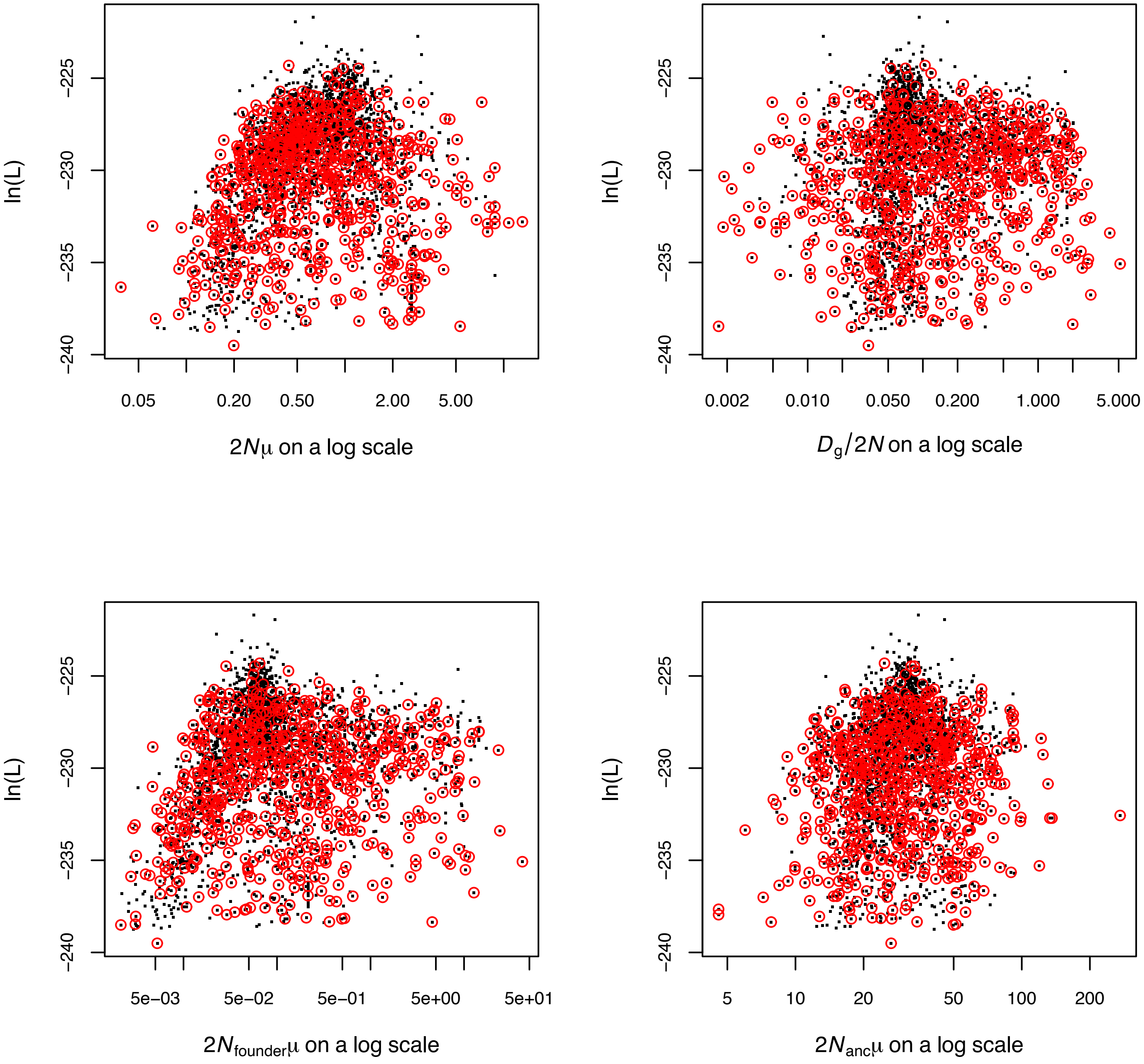
ln(L)
−225
−230
−235
−240
0.05
0.20
0.50
2.00
5.00
$2N\mu$ on a log scale
0.002
0.010
0.050
0.200
1.000
5.000
$D_g/2N$ on a log scale
5e−03
5e−02
5e−01
5e+00
5e+01
$2N_{founder}\mu$ on a log scale
5
10
20
50
100
200
$2N_{anc}\mu$ on a log scale

One−parameter likelihood ratio profiles

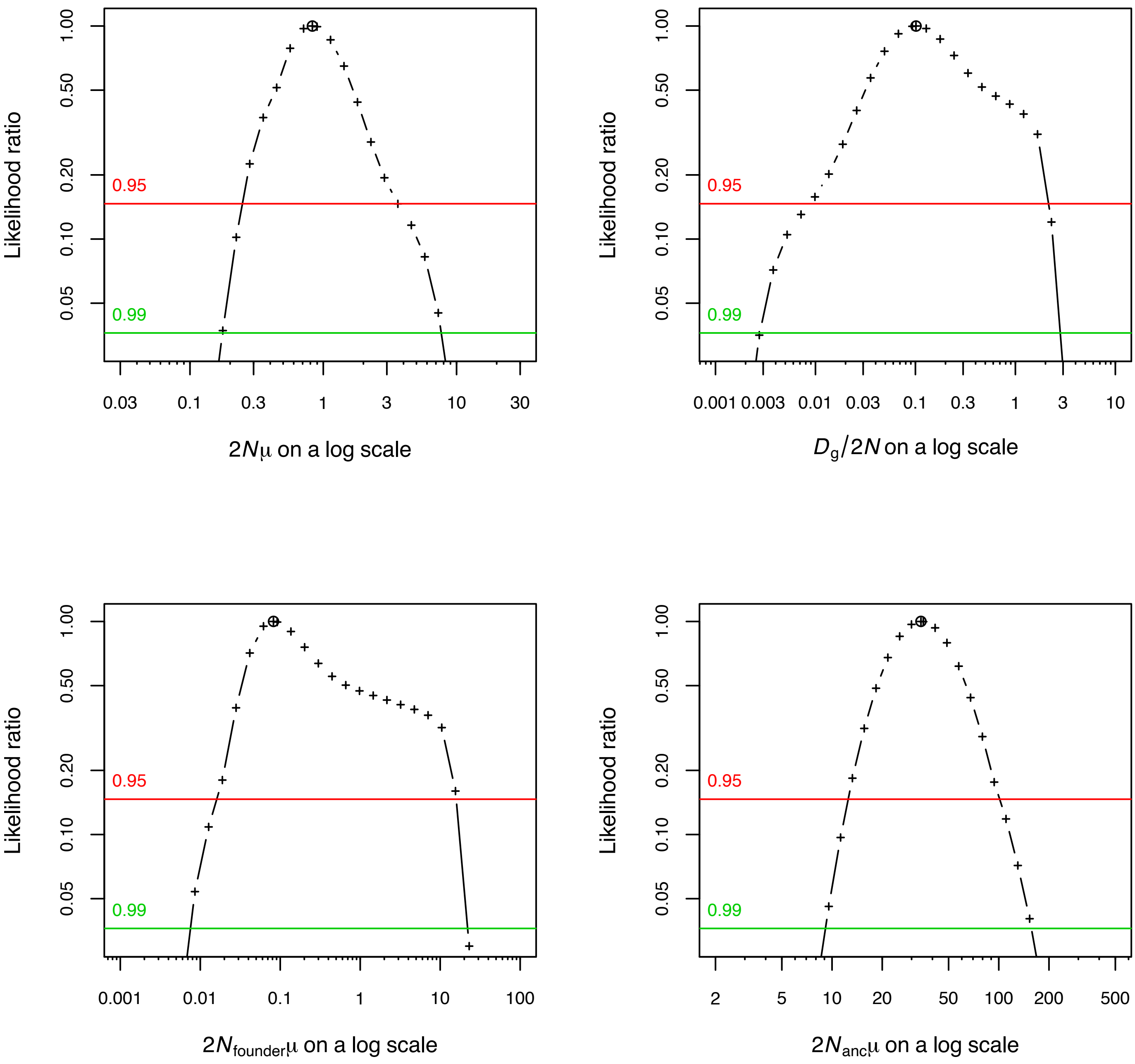

One–parameter likelihood ratio profiles

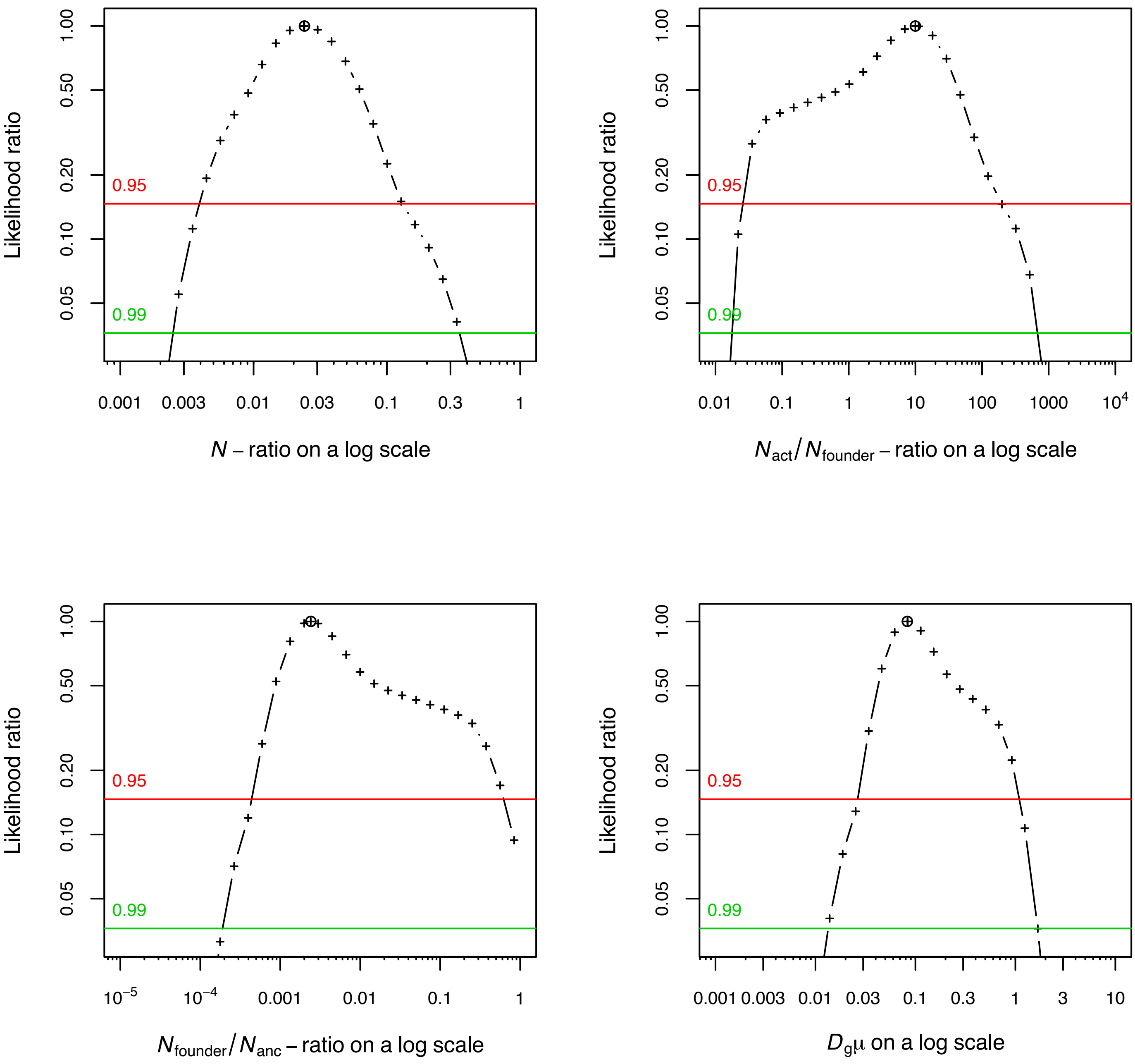

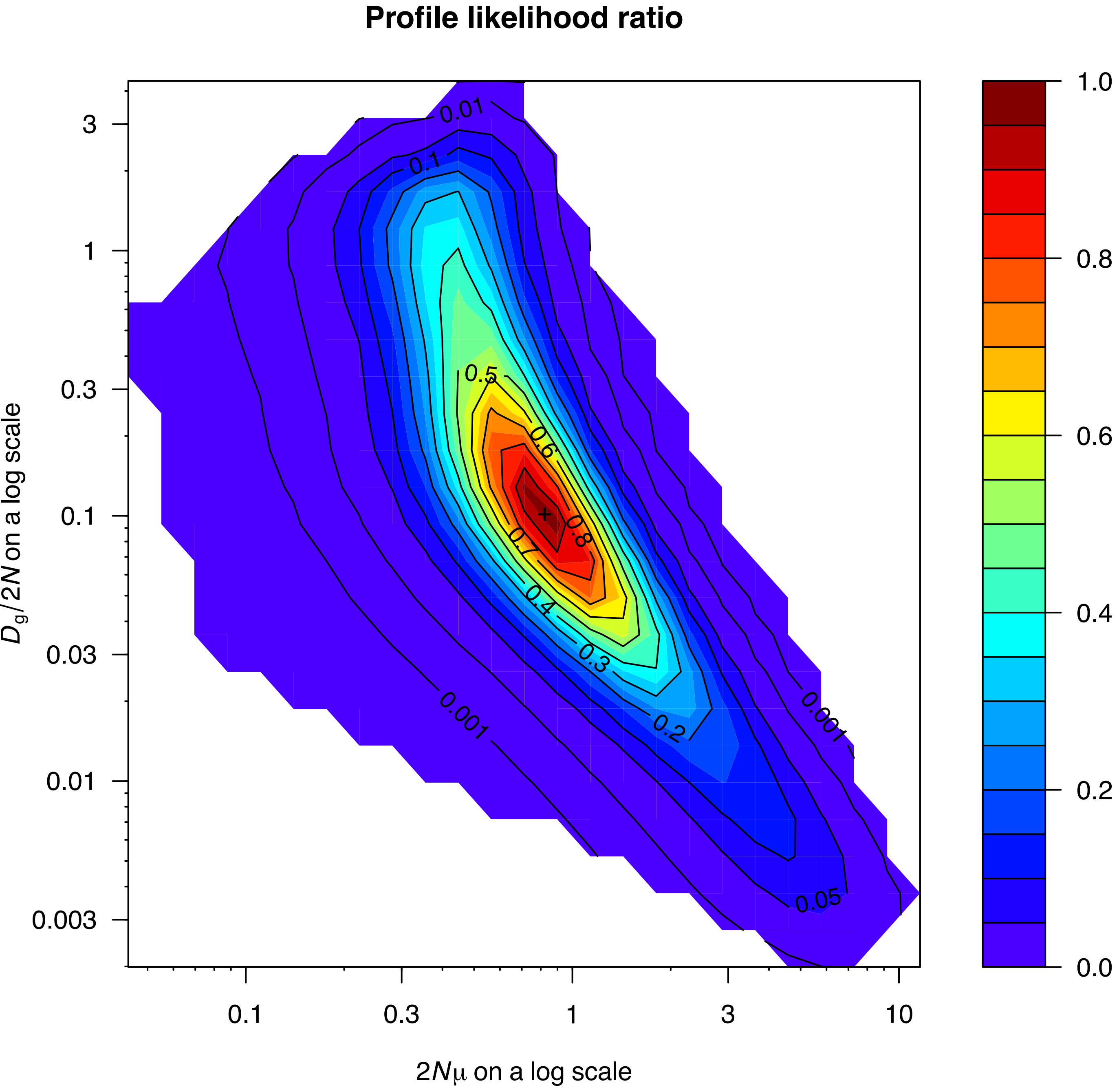
Profile likelihood ratio
0.01
0.1
0.5
0.6
0.8
0.7
0.4
0.3
0.2
0.001
0.001
0.05
3
1
0.3
0.1
0.03
0.01
0.003
0.1
0.3
1
3
10
1.0
0.8
0.6
0.4
0.2
0.0
$D_g/2N$ on a log scale
$2N\mu$ on a log scale

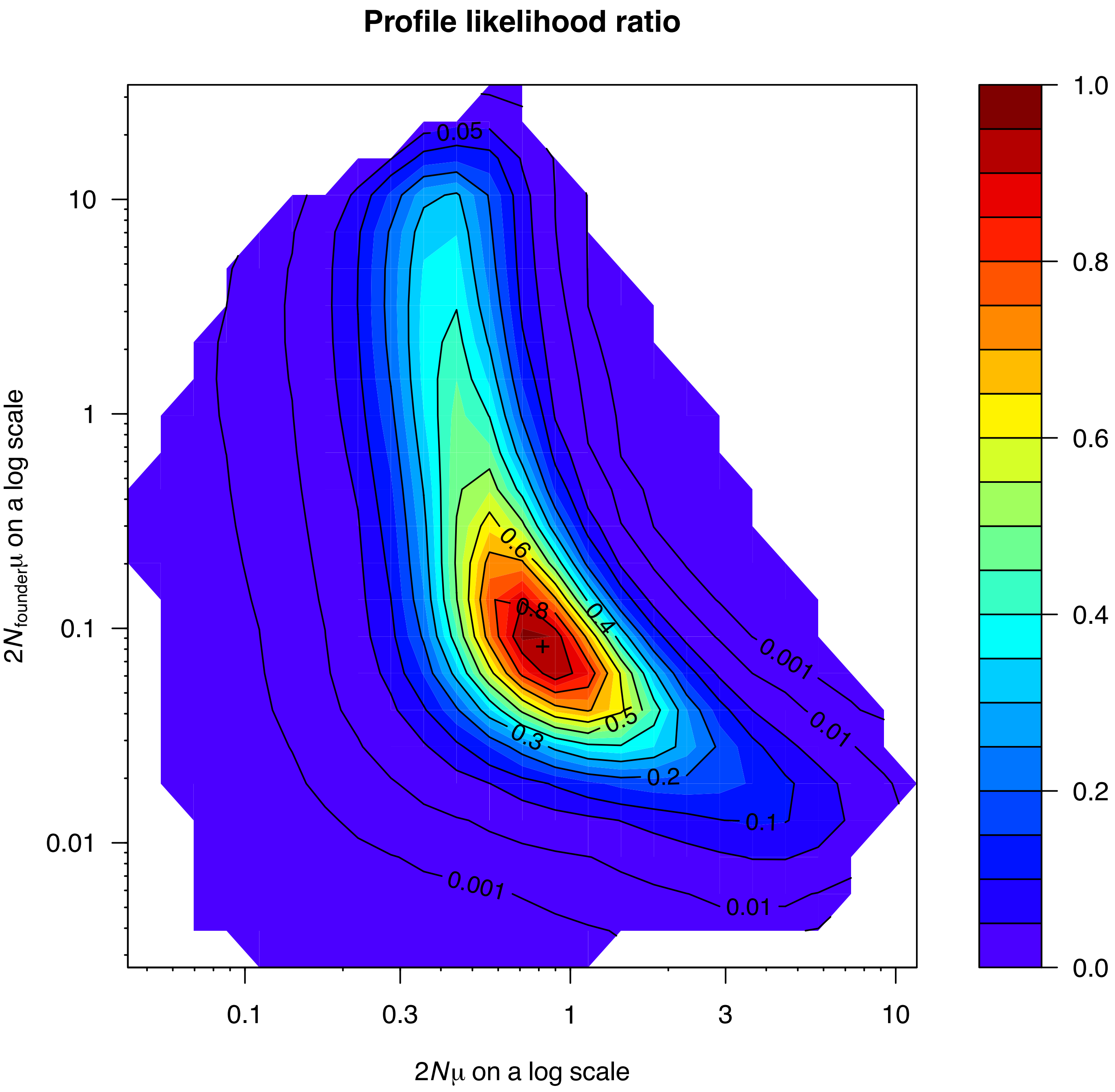

Profile likelihood ratio
2$N_{founder}$μ on a log scale
2Nμ on a log scale
10
1
0.1
0.01
0.1
0.3
1
3
10
0.05
0.6
0.8
0.4
0.5
0.3
0.2
0.1
0.001
0.01
0.001
0.01
1.0
0.8
0.6
0.4
0.2
0.0

Profile likelihood ratio

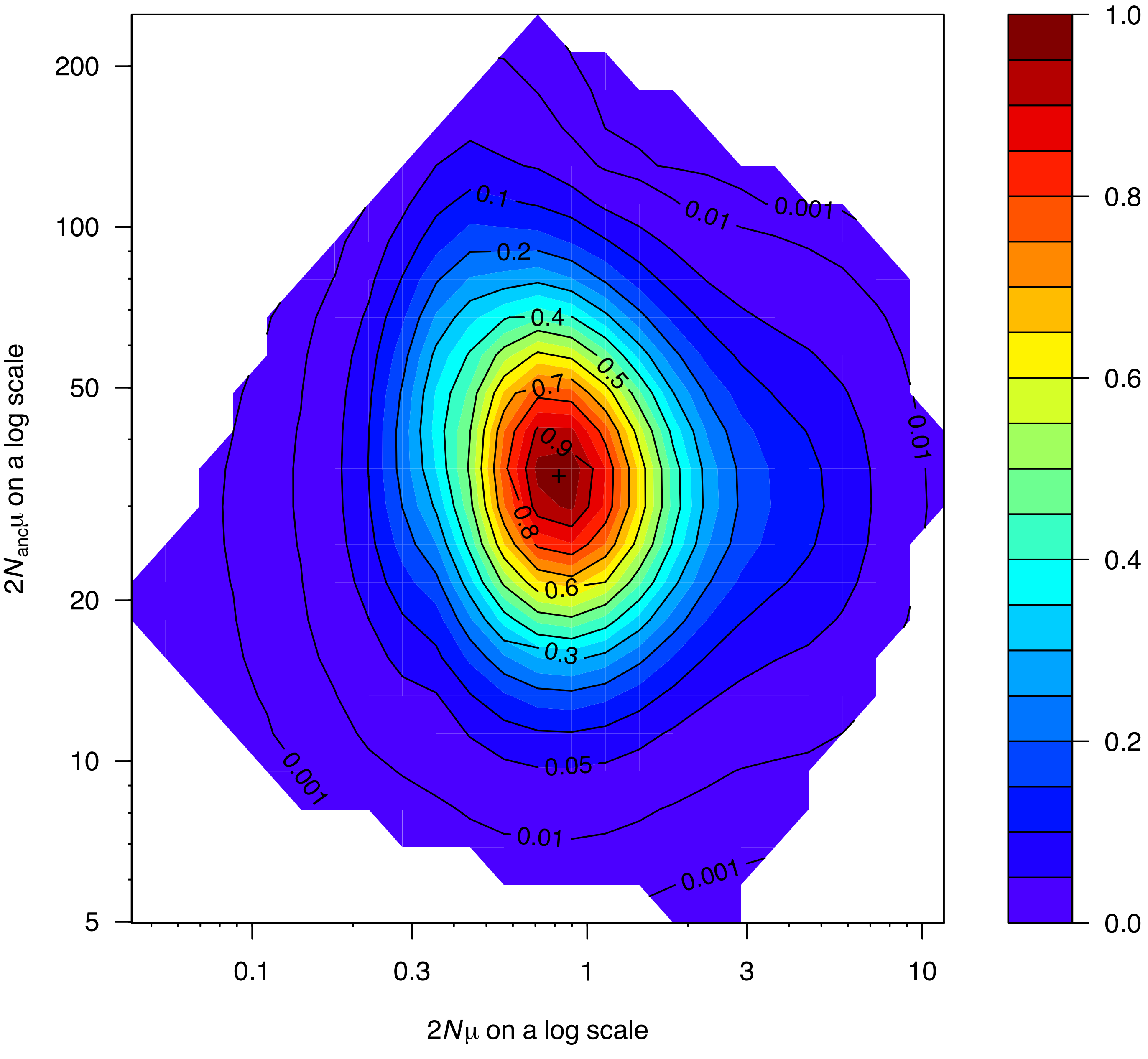

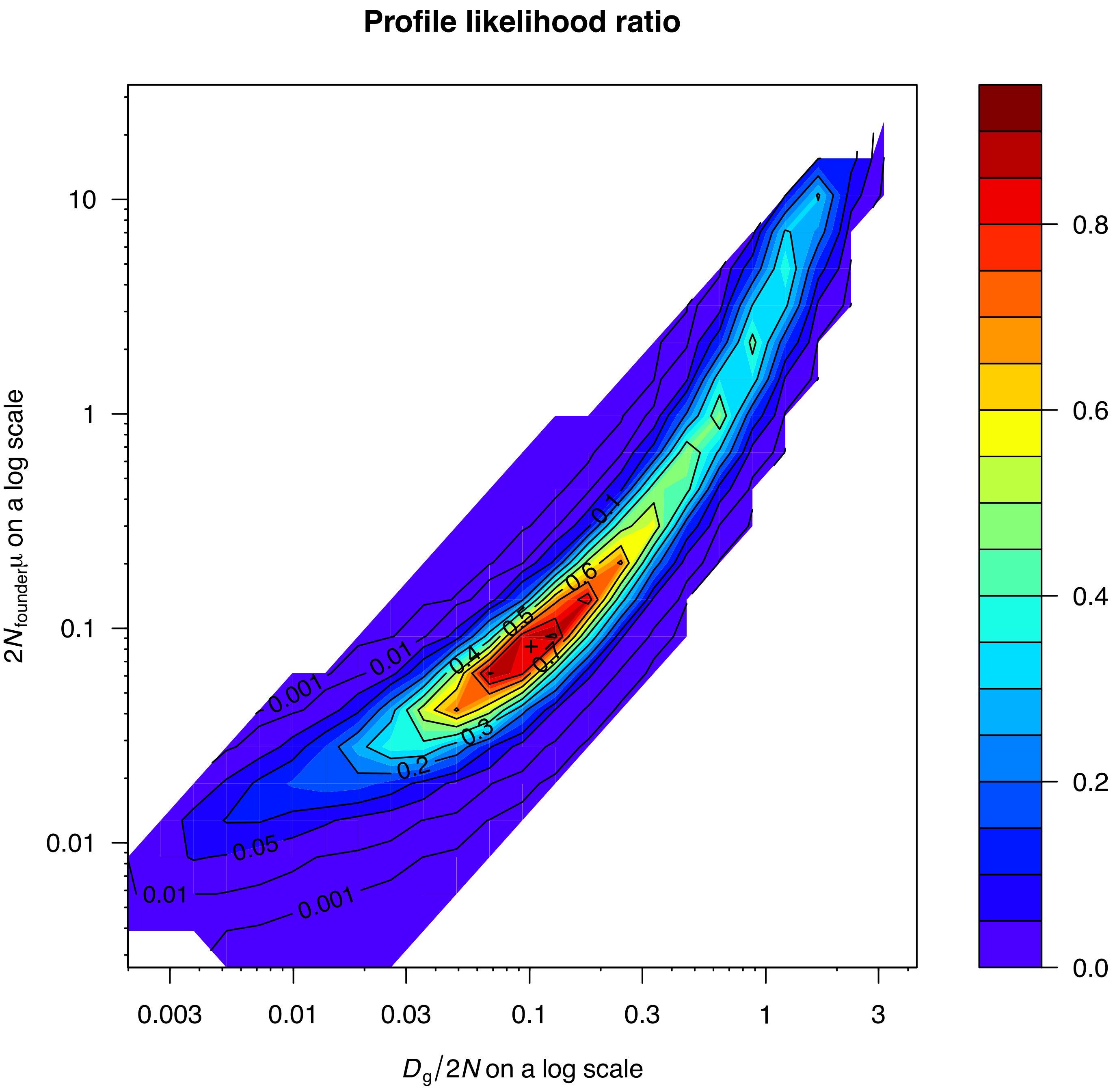

Profile likelihood ratio
$2N_{\text{founder}}\mu$ on a log scale
$D_g/2N$ on a log scale
0.003
0.01
0.03
0.1
0.3
1
3
10
0.01
0.1
0.0
0.2
0.4
0.6
0.8
0.001
0.01
0.05
0.1
0.2
0.3
0.4
0.5
0.6
0.7

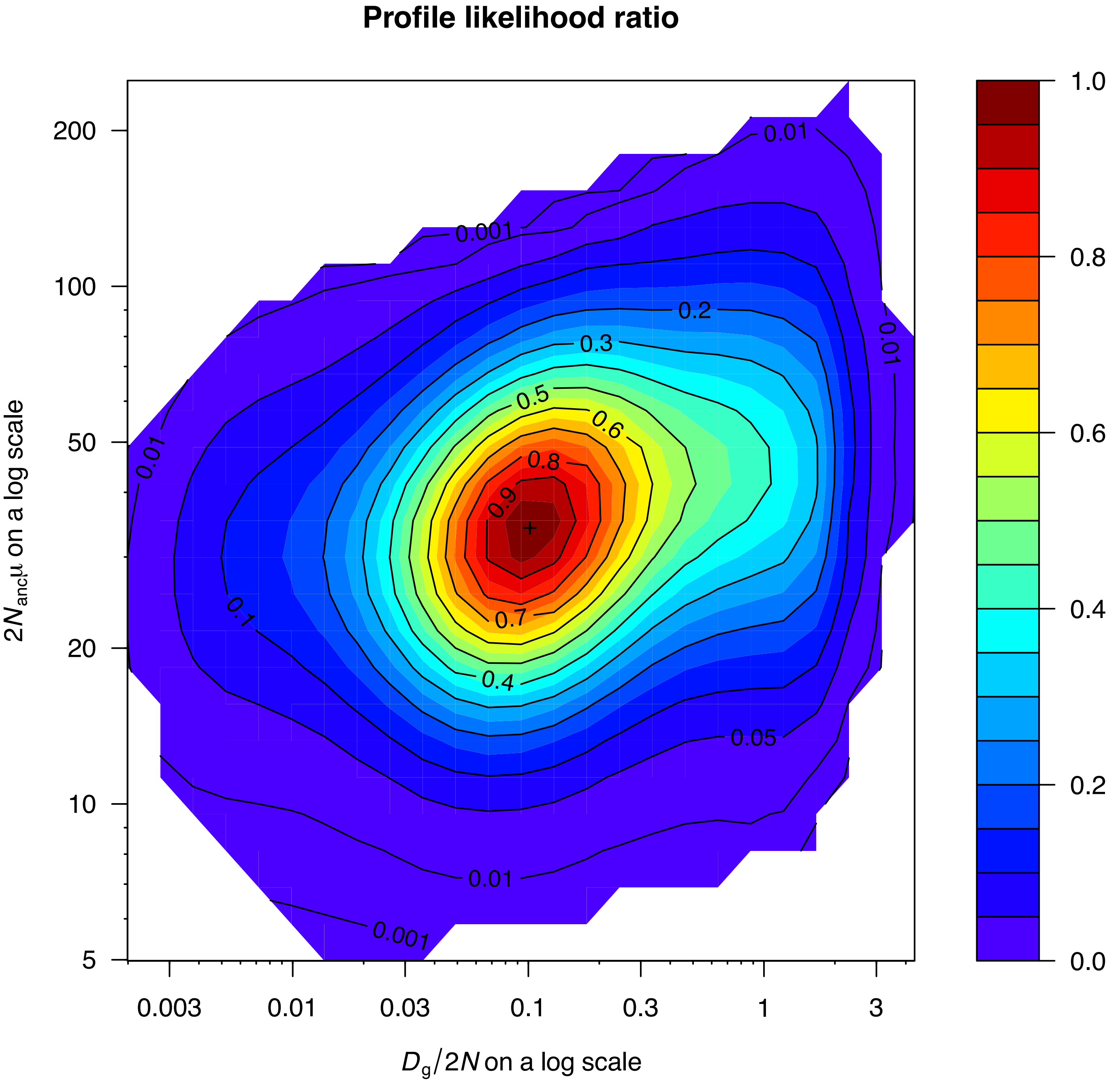

Profile likelihood ratio
200
100
50
20
10
5
0.003
0.01
0.03
0.1
0.3
1
3
$2N_{anc}\mu$ on a log scale
$D_g/2N$ on a log scale
0.001
0.01
0.2
0.3
0.5
0.6
0.8
0.9
0.7
0.4
0.1
0.05
1.0
0.8
0.6
0.4
0.2
0.0

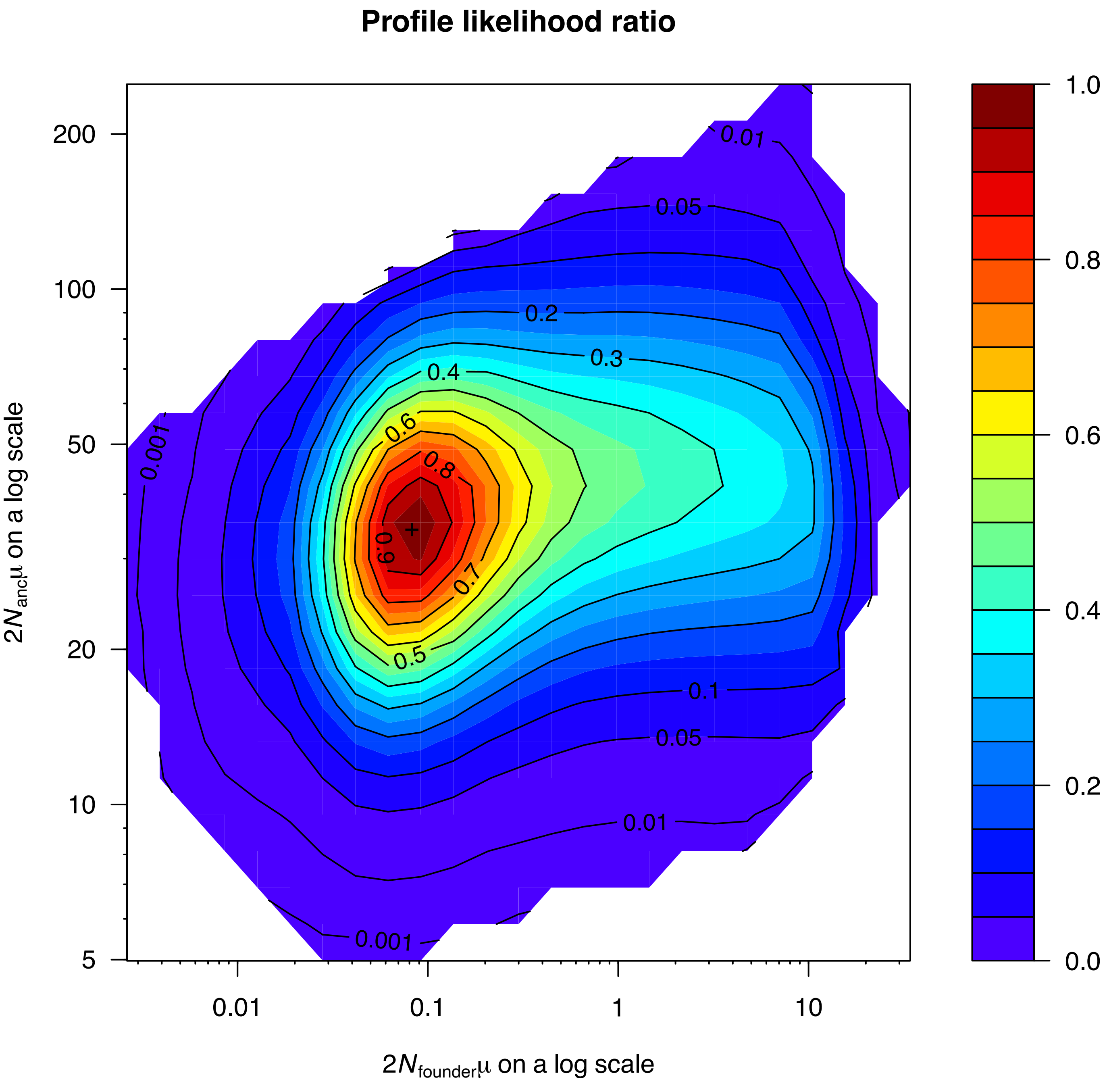
Profile likelihood ratio
200
100
50
20
10
5
0.01
0.1
1
10
$2N_{anc}\mu$ on a log scale
$2N_{founder}\mu$ on a log scale
0.001
0.01
0.05
0.1
0.2
0.3
0.4
0.5
0.6
0.7
0.8
0.9
1.0
0.8
0.6
0.4
0.2
0.0

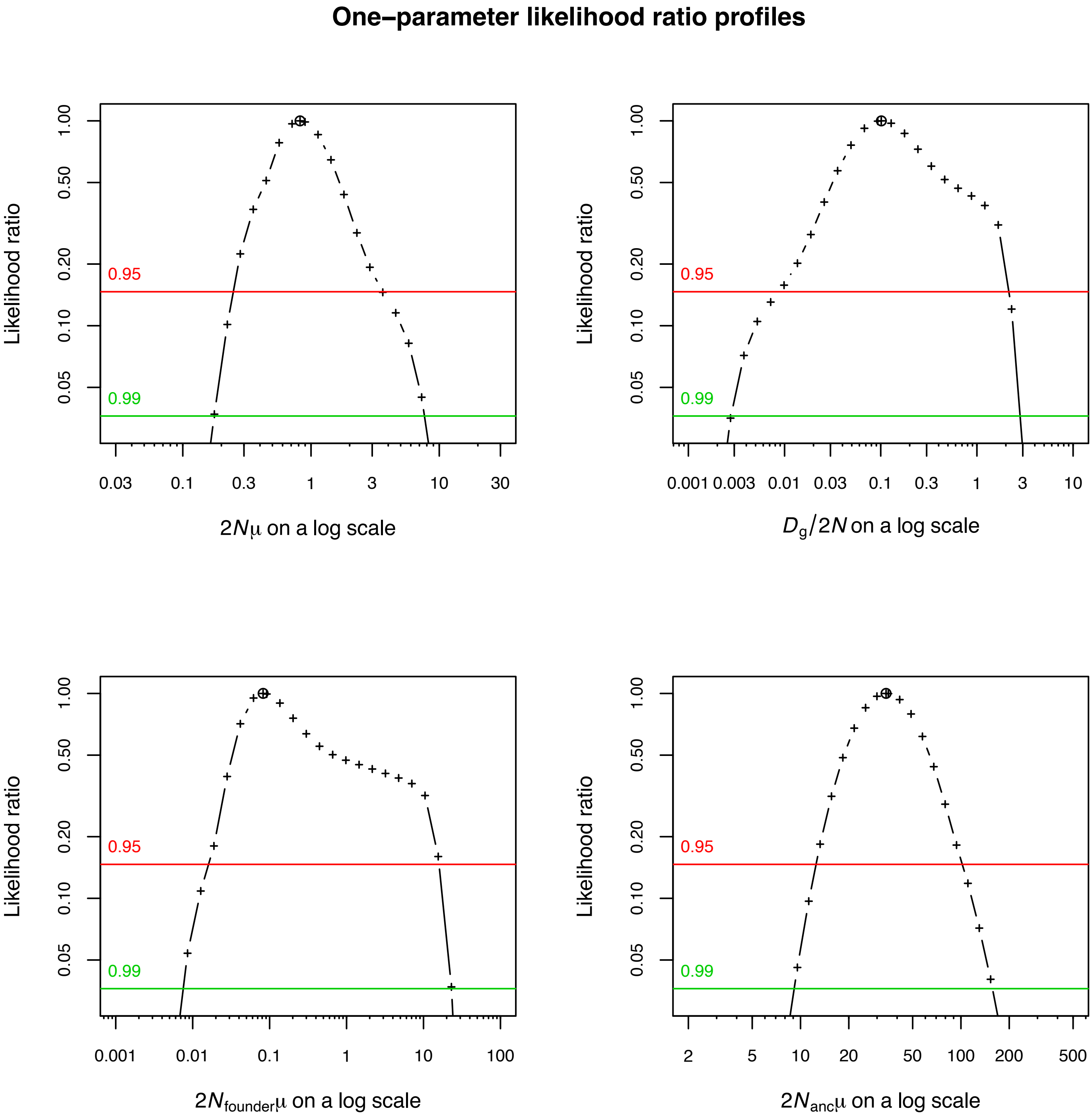

One−parameter likelihood ratio profiles
Likelihood ratio
1.00
0.50
0.20
0.10
0.05
0.95
0.99
0.03
0.1
0.3
1
3
10
30
2Nμ on a log scale
0.001
0.003
0.01
0.03
0.1
0.3
1
3
10
Dg/2N on a log scale
0.001
0.01
0.1
1
10
100
2Nfounderμ on a log scale
2
5
10
20
50
100
200
500
2Nancμ on a log scale

One–parameter likelihood ratio profiles

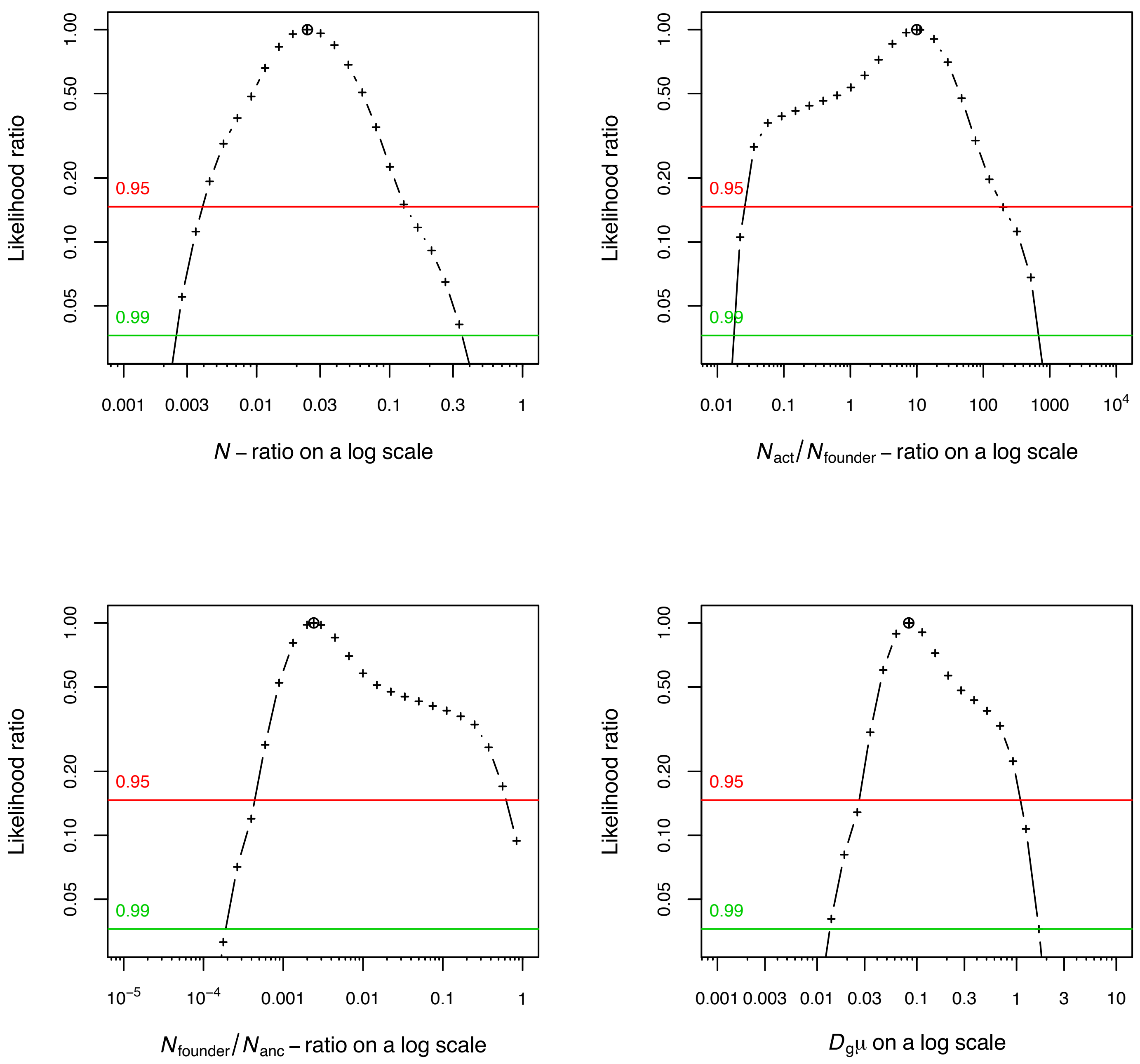

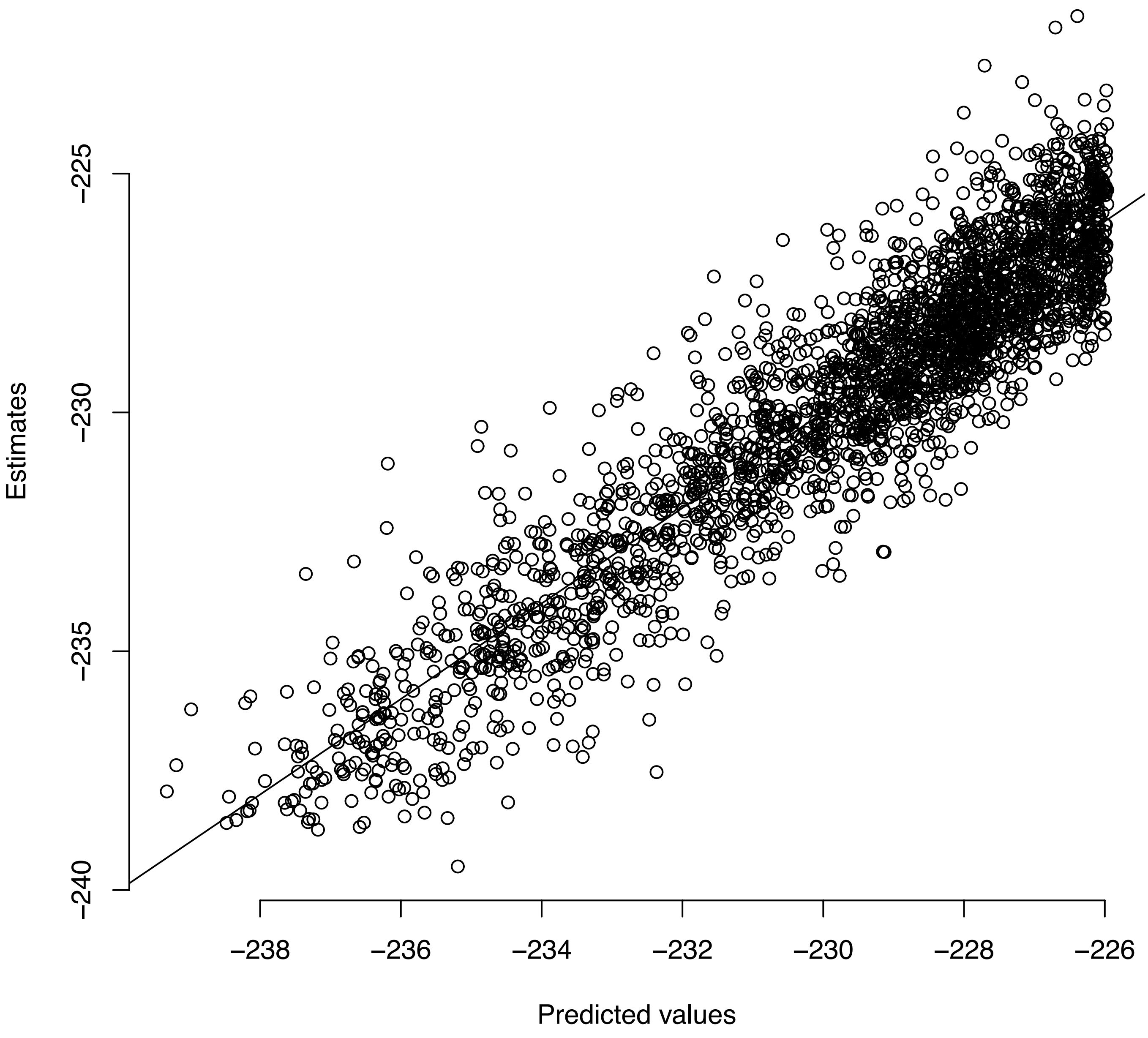

Estimates
−225
−230
−235
−240
−238
−236
−234
−232
−230
−228
−226
Predicted values

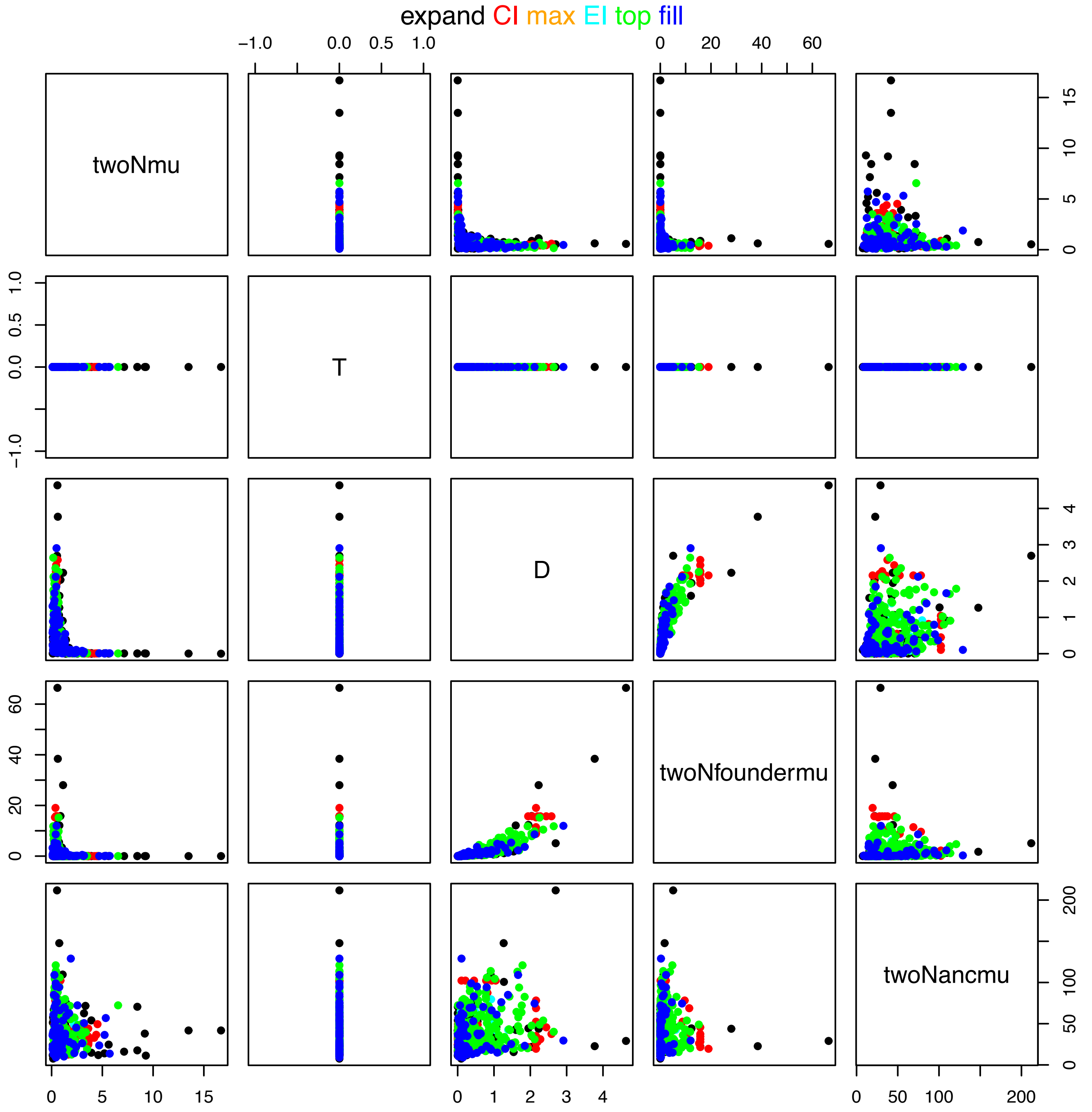
expand CI max EI top fill
twoNmu
T
D
twoNfoundermu
twoNancmu
−1.0
0.0
0.5
1.0
0
20
40
60
15
10
5
0
4
3
2
1
200
100
50
10
