# Supplementary material for: Origin and dispersion pathways of guava in the Galapagos Islands inferred through genetics and historical records
Source: Ecol Evol. 2021 Oct 4;11(21):15111–31. doi: 10.1002/ece3.8193 (PMC8571588; doi:10.1002/ece3.8193)
Supplement: Supplementary file 4 — Supplementary Material [file ECE3-11-15111-s008.pdf]

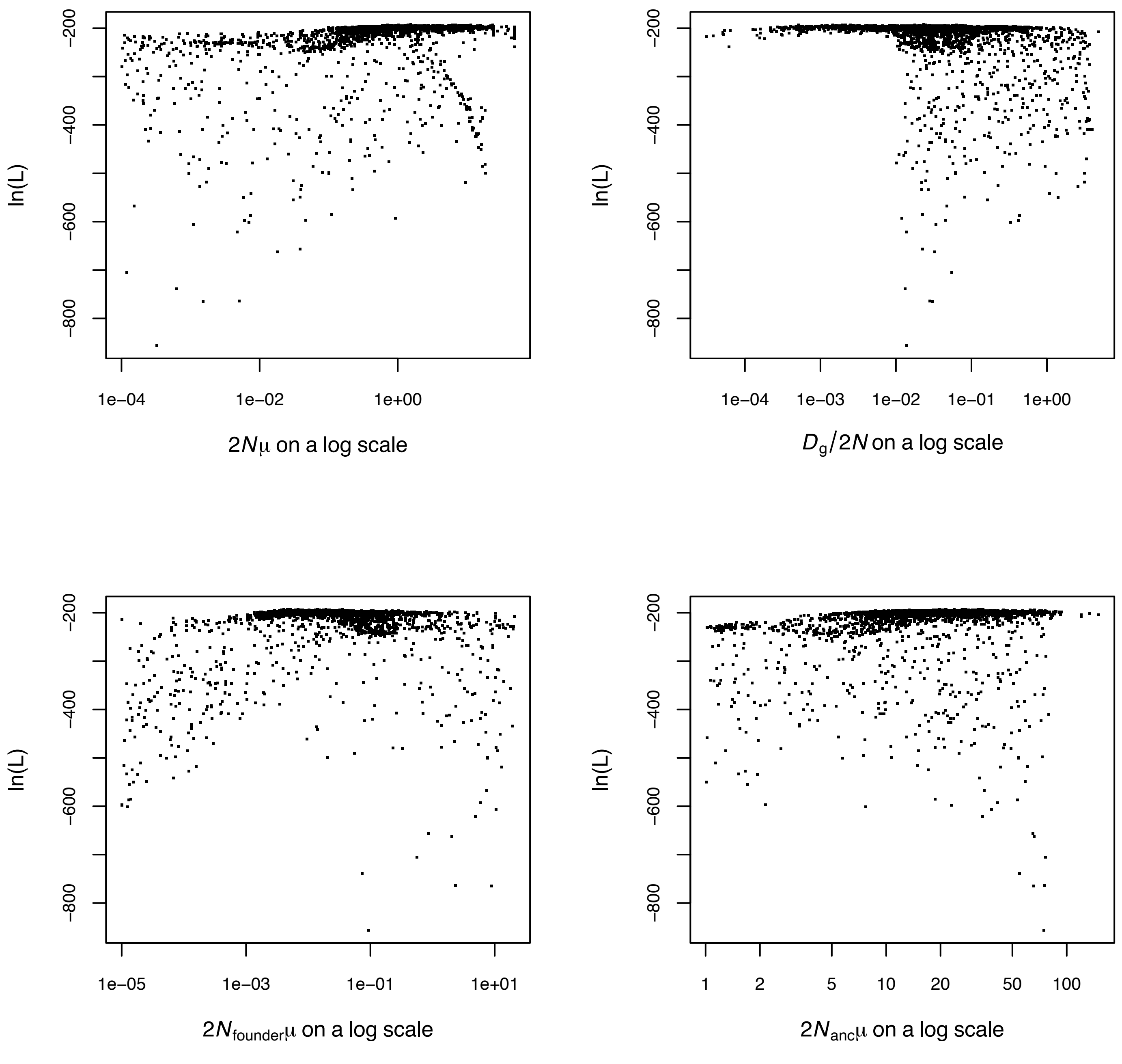
ln(L)
-200
-400
-600
-800
1e-04
1e-02
1e+00
2$N\mu$ on a log scale
1e-03
1e-01
$D_g/2N$ on a log scale
1e-05
1e+01
2$N_{founder}\mu$ on a log scale
1
2
5
10
20
50
100
2$N_{anc}\mu$ on a log scale

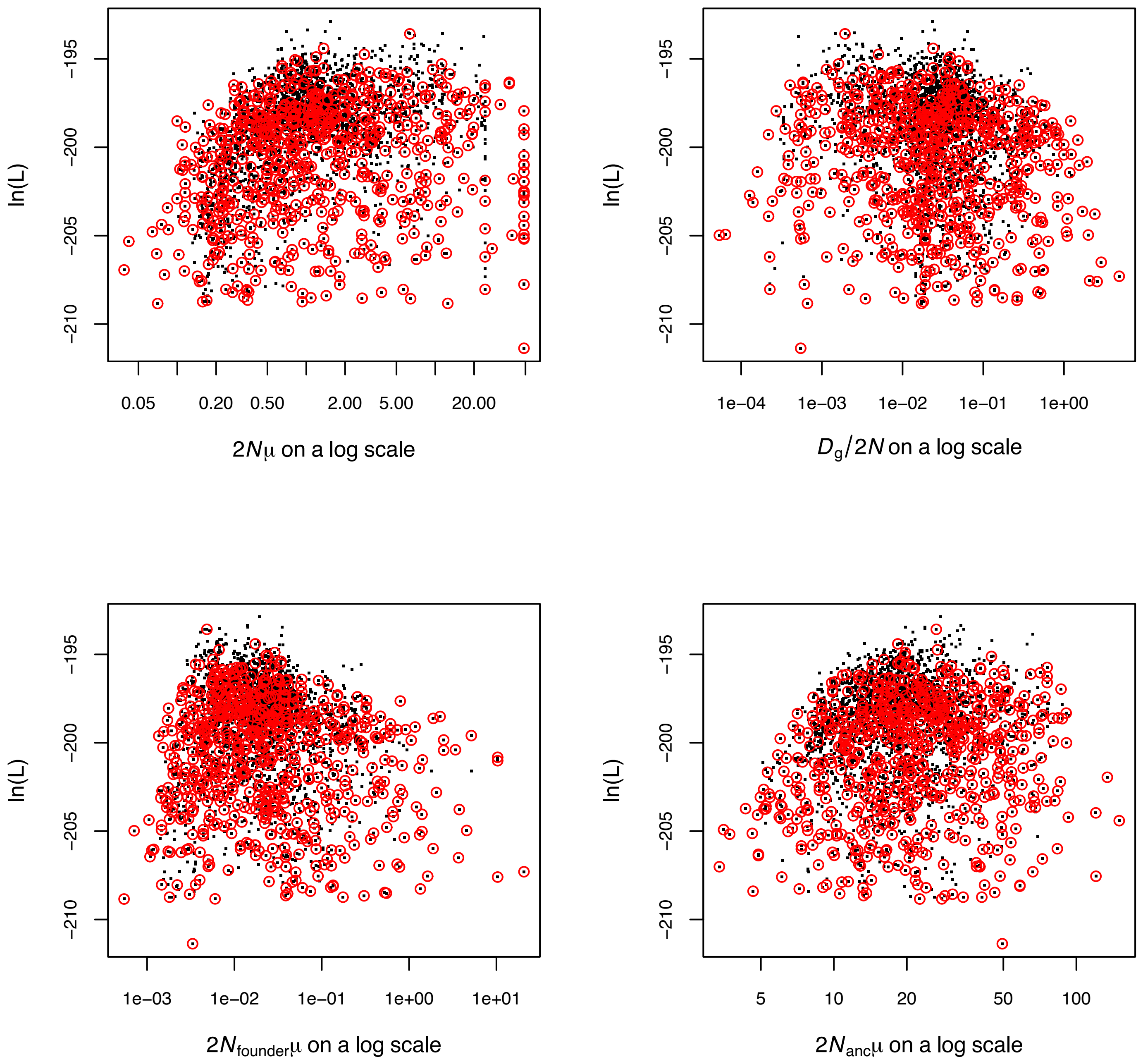

ln(L)
−195
−200
−205
−210
0.05
0.20
0.50
2.00
5.00
20.00
$2N\mu$ on a log scale
1e−04
1e−03
1e−02
1e−01
1e+00
$D_g/2N$ on a log scale
1e−03
1e−02
1e−01
1e+00
1e+01
$2N_{founder}\mu$ on a log scale
5
10
20
50
100
$2N_{anc}\mu$ on a log scale

One–parameter likelihood ratio profiles

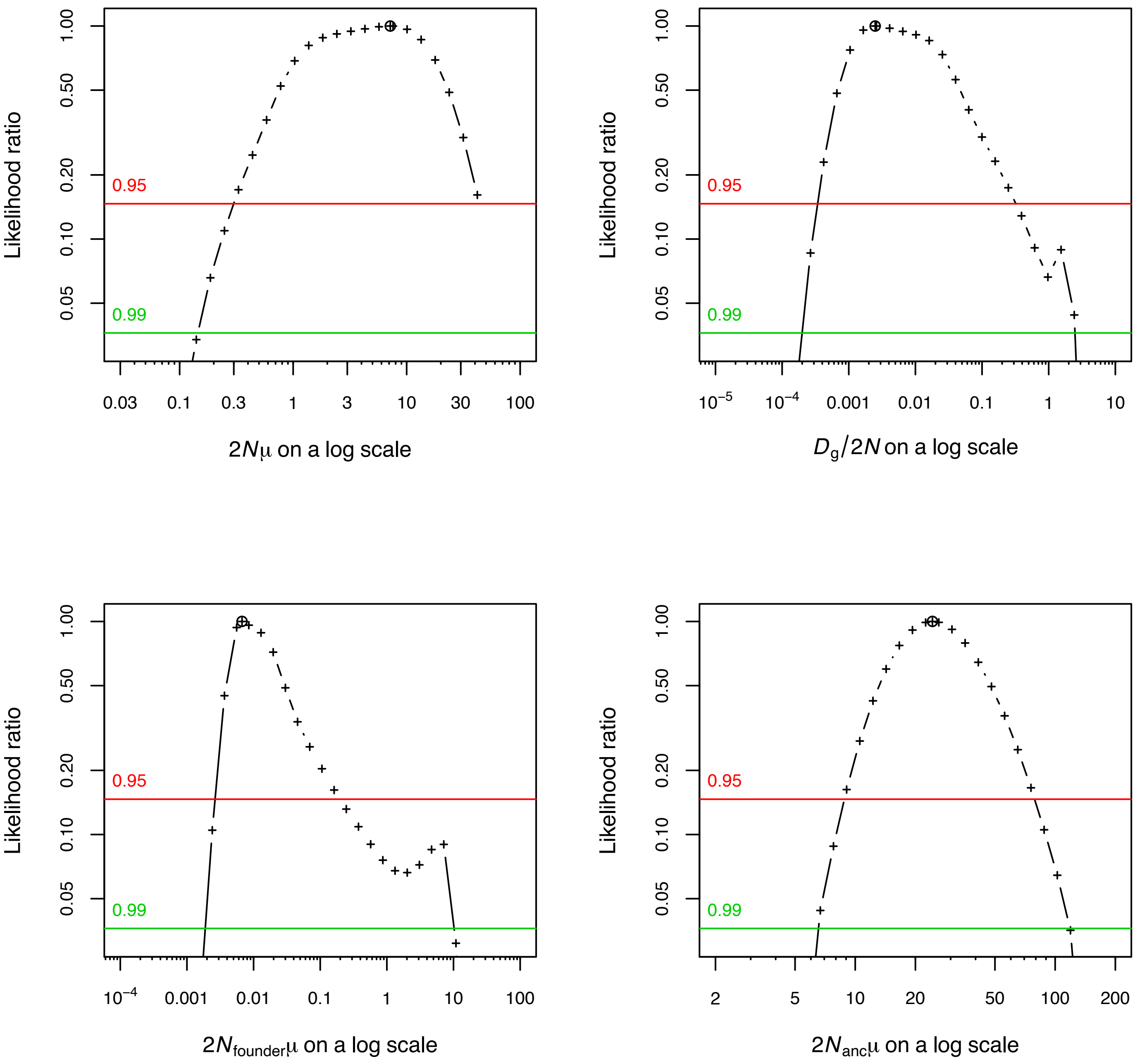

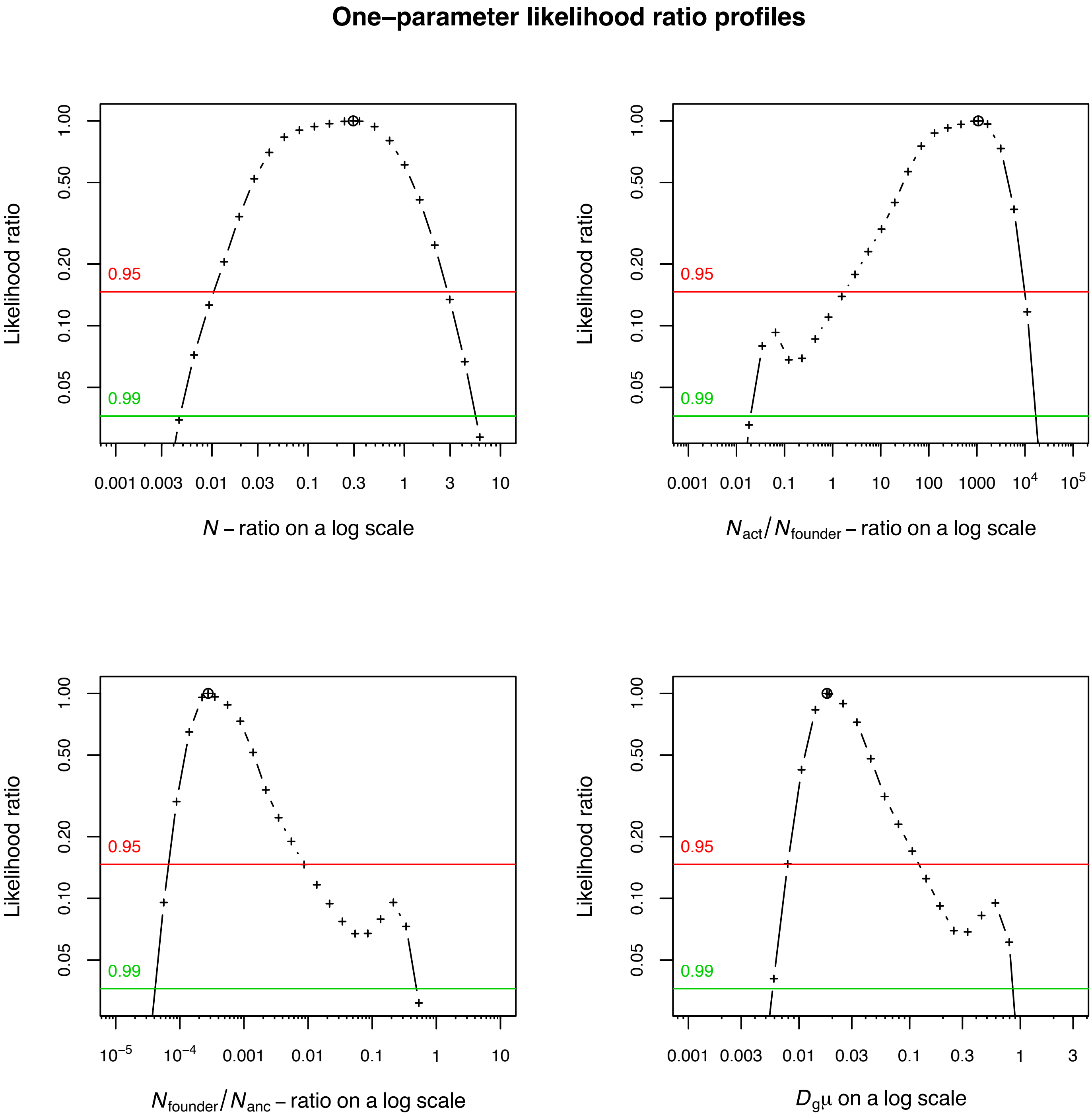

One−parameter likelihood ratio profiles
Likelihood ratio
0.95
0.99
N − ratio on a log scale
Nact / Nfounder − ratio on a log scale
Nfounder / Nanc − ratio on a log scale
Dgμ on a log scale

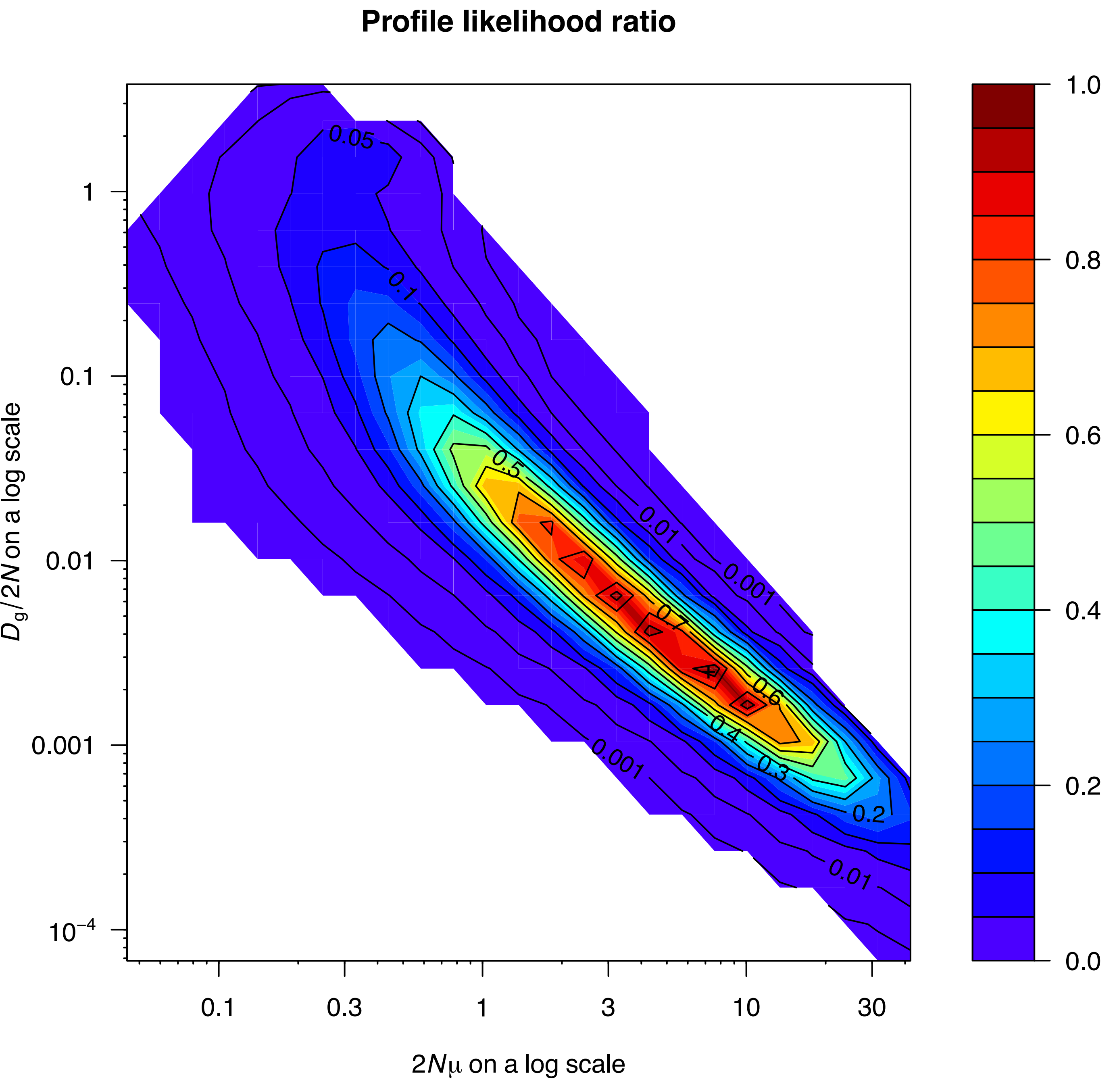
Profile likelihood ratio
0.05
0.1
0.5
0.01
0.001
0.7
0.6
0.4
0.3
0.2
0.001
0.01
1
0.1
0.01
0.001
10^{-4}
0.1
0.3
1
3
10
30
$D_g/2N$ on a log scale
$2N\mu$ on a log scale
1.0
0.8
0.6
0.4
0.2
0.0

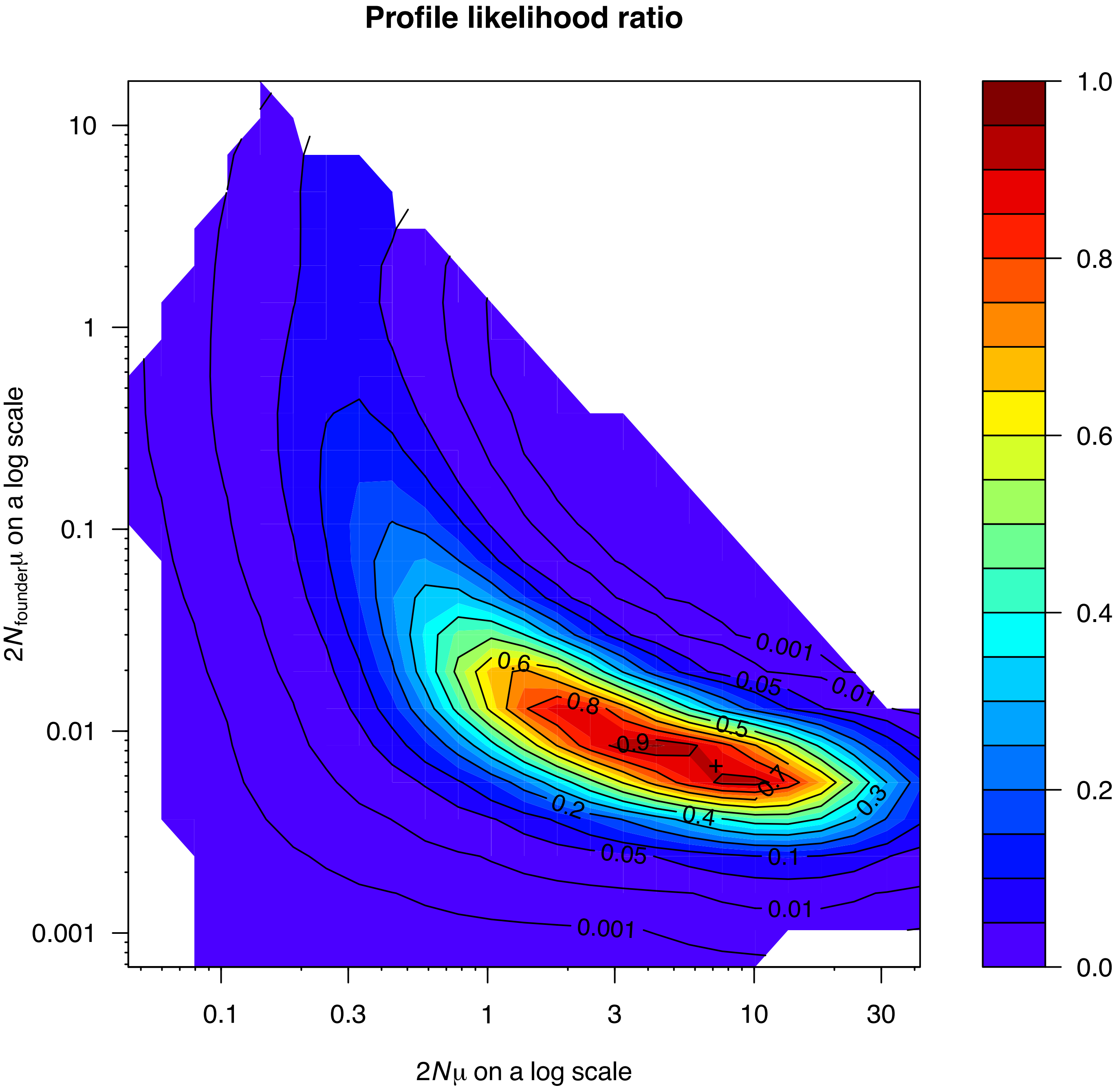

Profile likelihood ratio
$2N_{founder}\mu$ on a log scale
$2N\mu$ on a log scale
10
1
0.1
0.01
0.001
0.1
0.3
1
3
10
30
0.001
0.01
0.05
0.1
0.2
0.3
0.4
0.5
0.6
0.7
0.8
0.9
1.0
0.8
0.6
0.4
0.2
0.0

Profile likelihood ratio

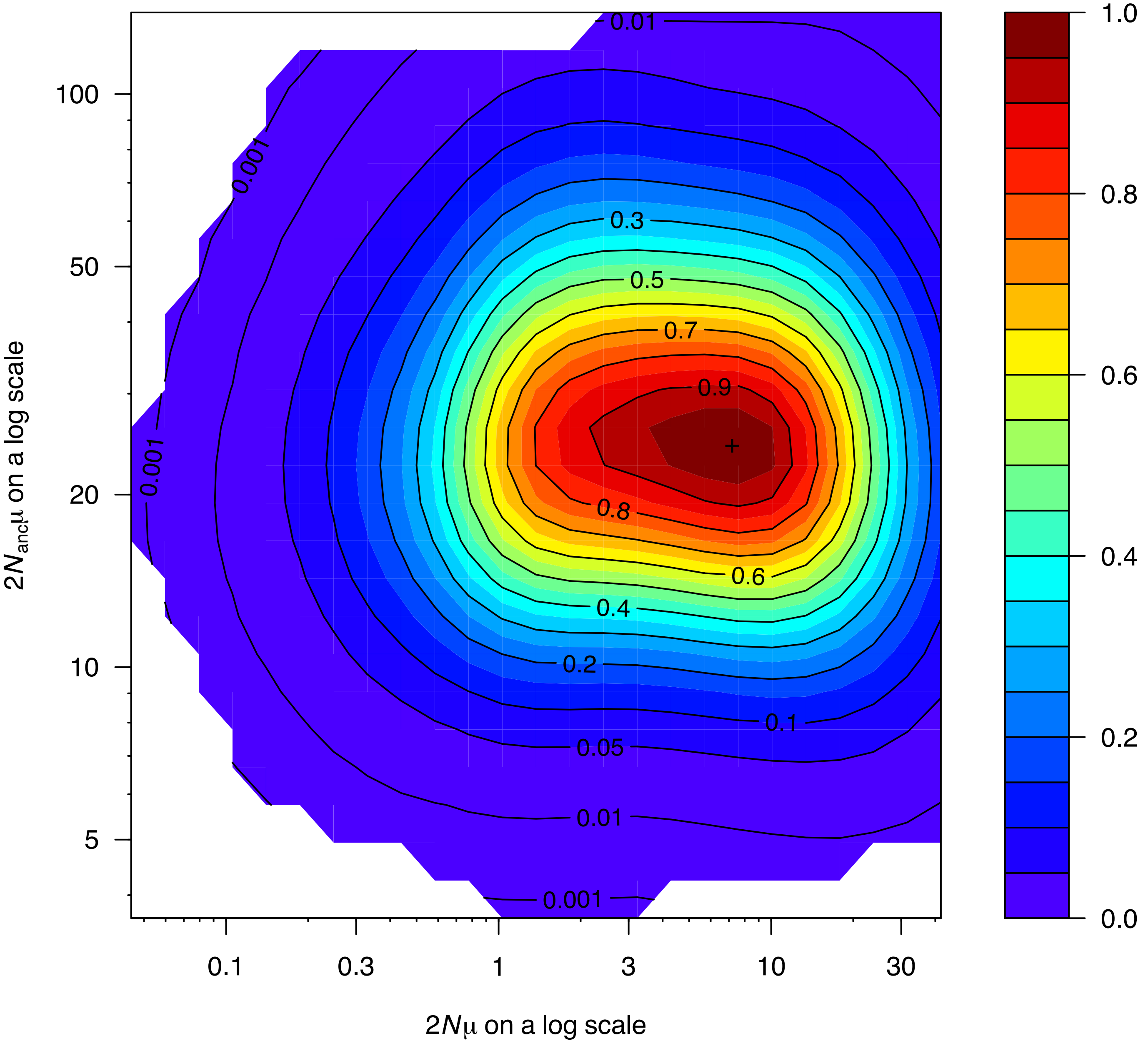

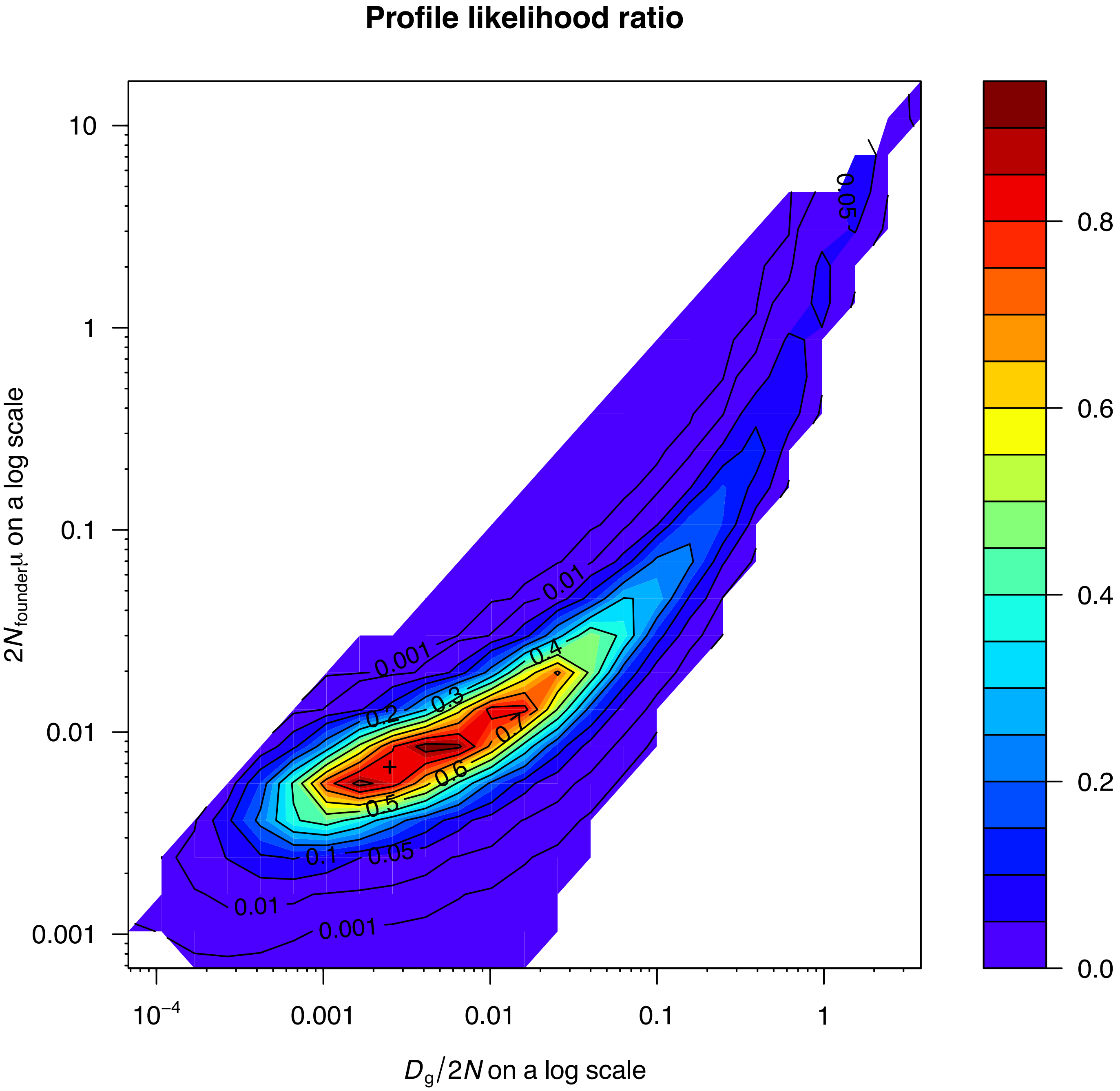
Profile likelihood ratio
$2N_{\text{founder}}\mu$ on a log scale
$D_g/2N$ on a log scale
10
1
0.1
0.01
0.001
10^{-4}
0.001
0.01
0.1
1
0.0
0.2
0.4
0.6
0.8
0.001
0.01
0.05
0.1
0.2
0.3
0.4
0.5
0.6
0.7

Profile likelihood ratio

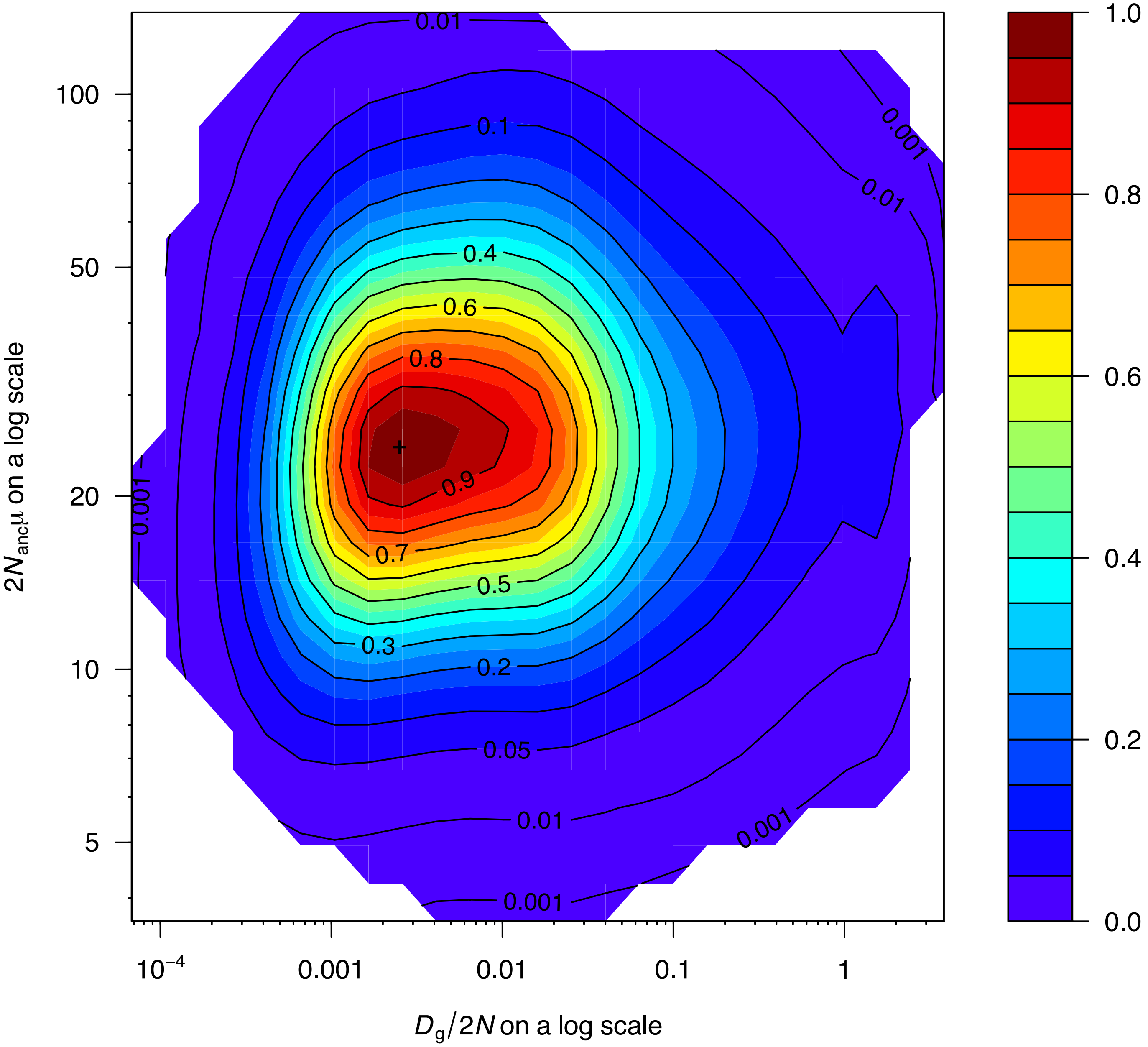

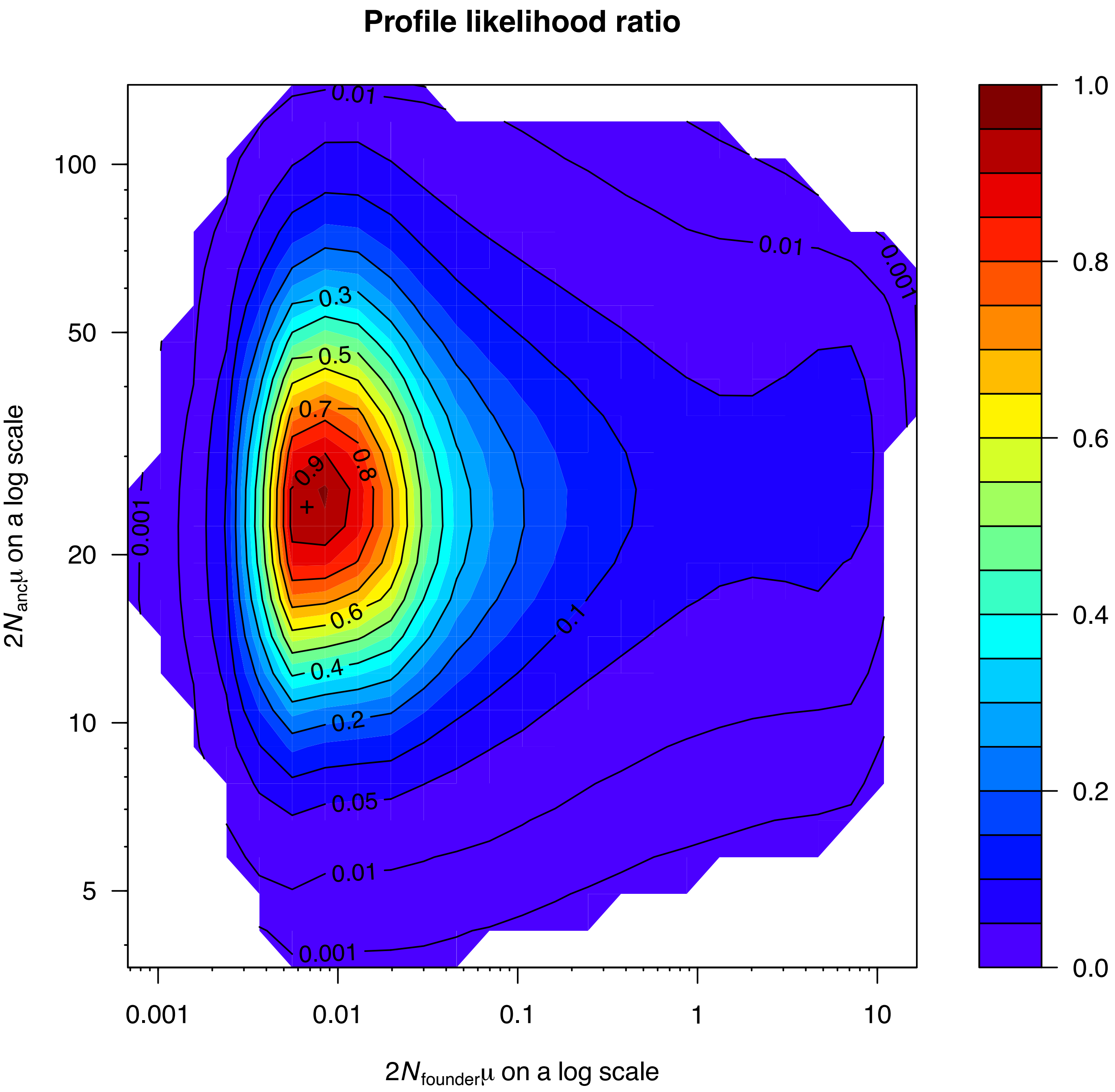

Profile likelihood ratio
0.01
0.01
0.001
0.3
0.5
0.7
0.9
0.8
0.6
0.4
0.2
0.1
0.05
0.01
0.001
0.001
100
50
20
10
5
0.001
0.01
0.1
1
10
$2N_{anc}\mu$ on a log scale
$2N_{founder}\mu$ on a log scale
1.0
0.8
0.6
0.4
0.2
0.0

One–parameter likelihood ratio profiles

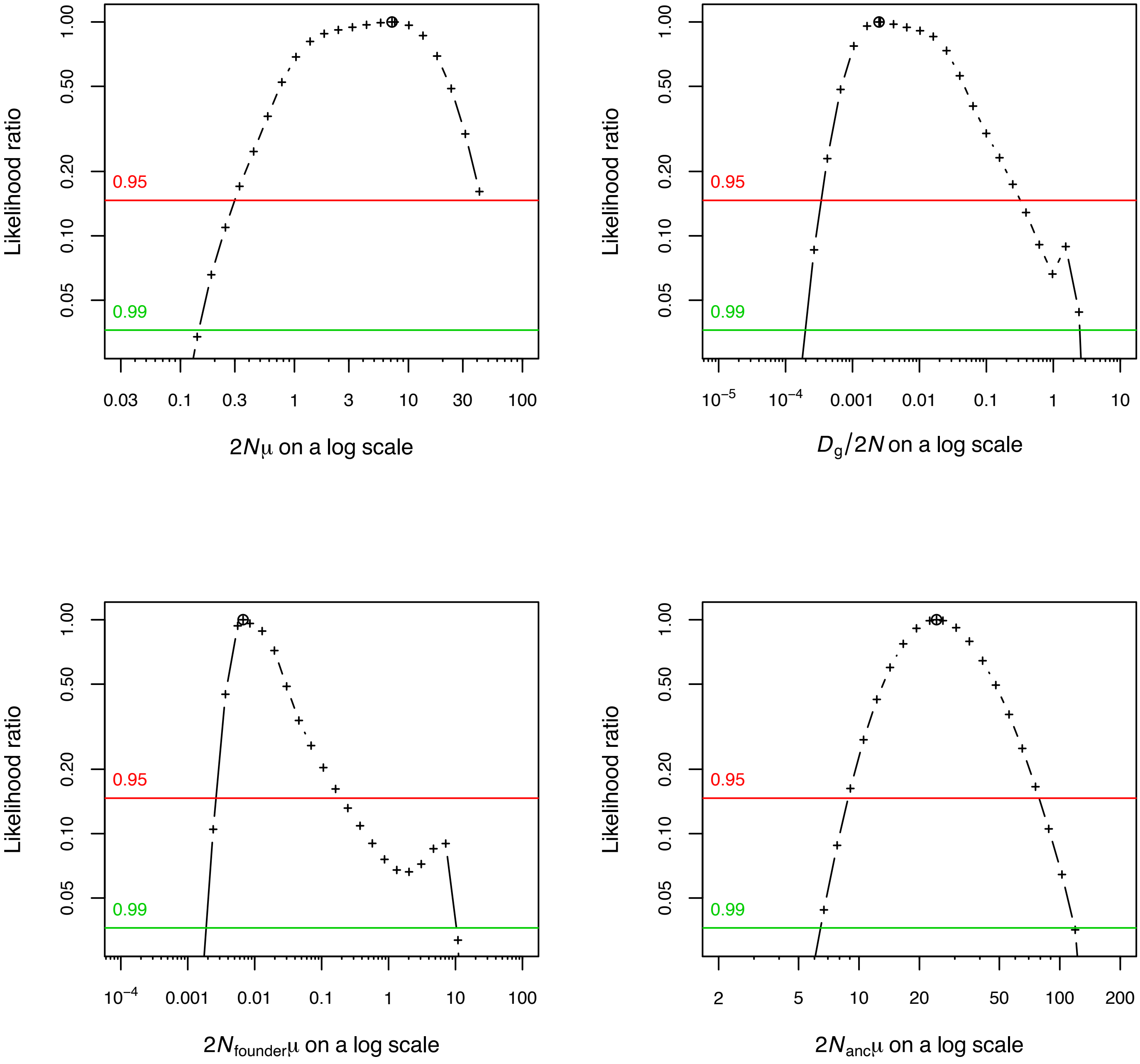

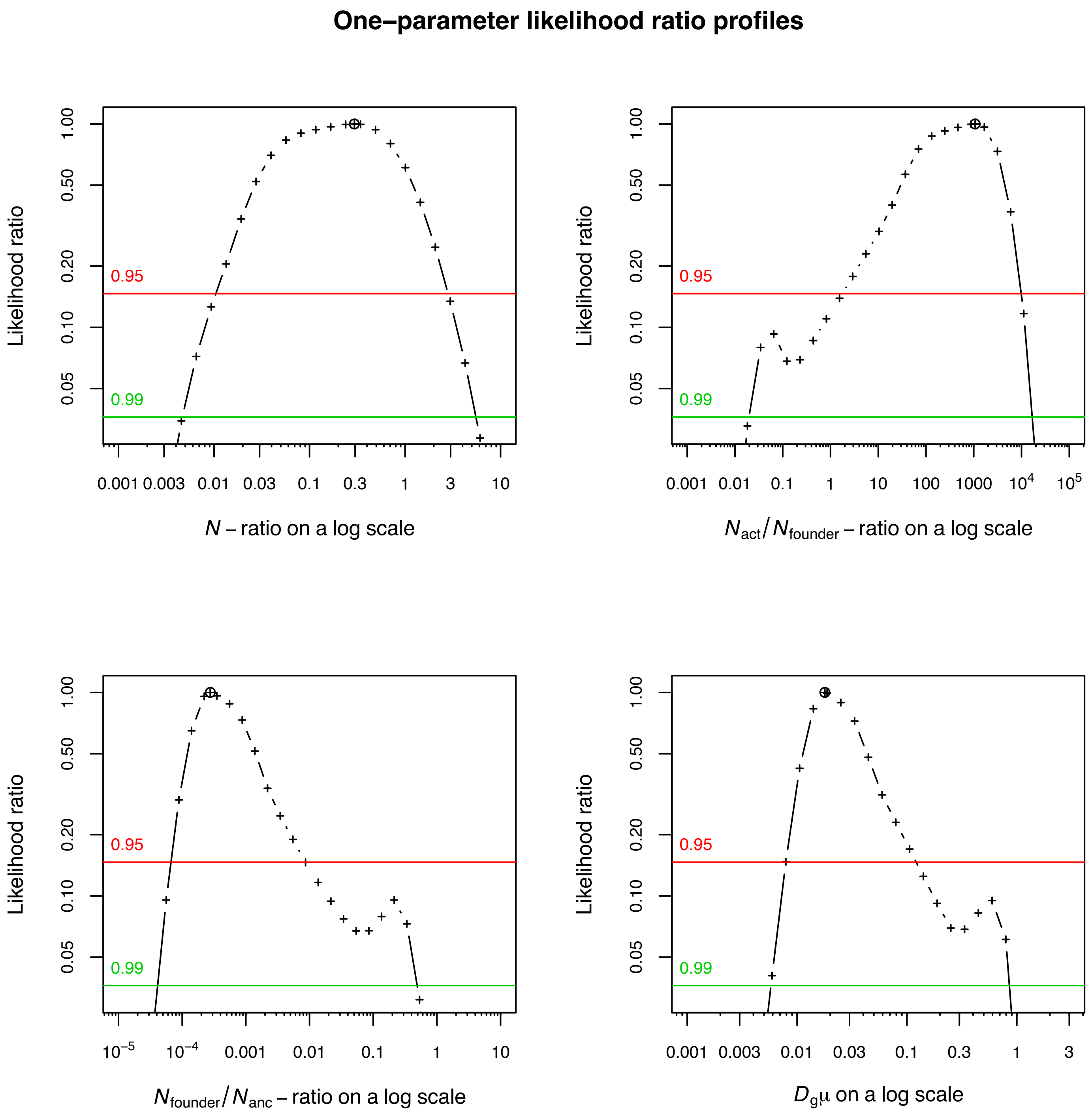
One–parameter likelihood ratio profiles
Likelihood ratio
1.00
0.50
0.20
0.10
0.05
0.95
0.99
0.001 0.003 0.01 0.03 0.1 0.3 1 3 10
N – ratio on a log scale
0.001 0.01 0.1 1 10 100 1000 10^4 10^5
N_act / N_founder – ratio on a log scale
10^−5 10^−4 0.001 0.01 0.1 1 10
N_founder / N_anc – ratio on a log scale
0.001 0.003 0.01 0.03 0.1 0.3 1 3
D_g μ on a log scale

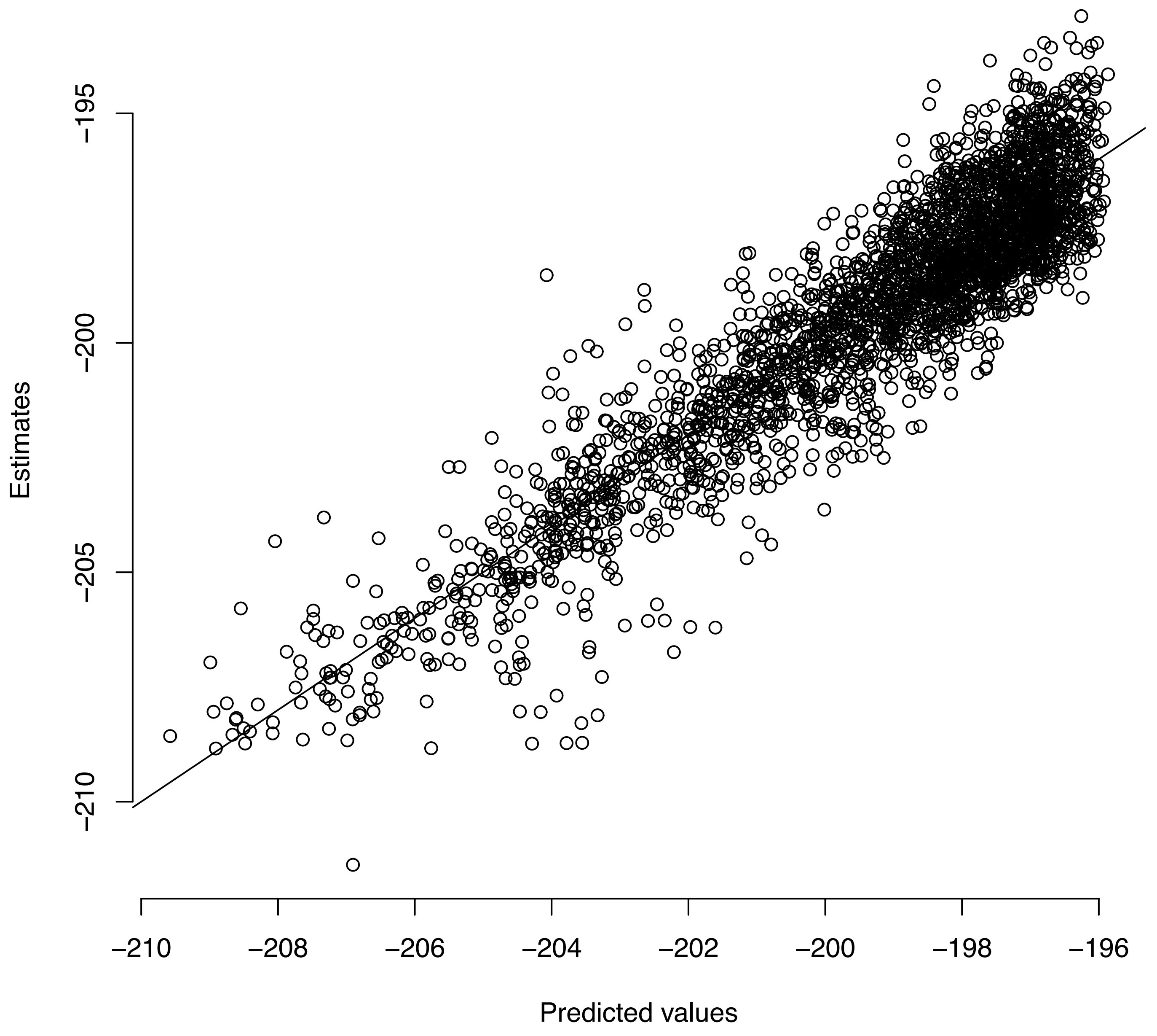

Estimates
−195
−200
−205
−210
−210
−208
−206
−204
−202
−200
−198
−196
Predicted values

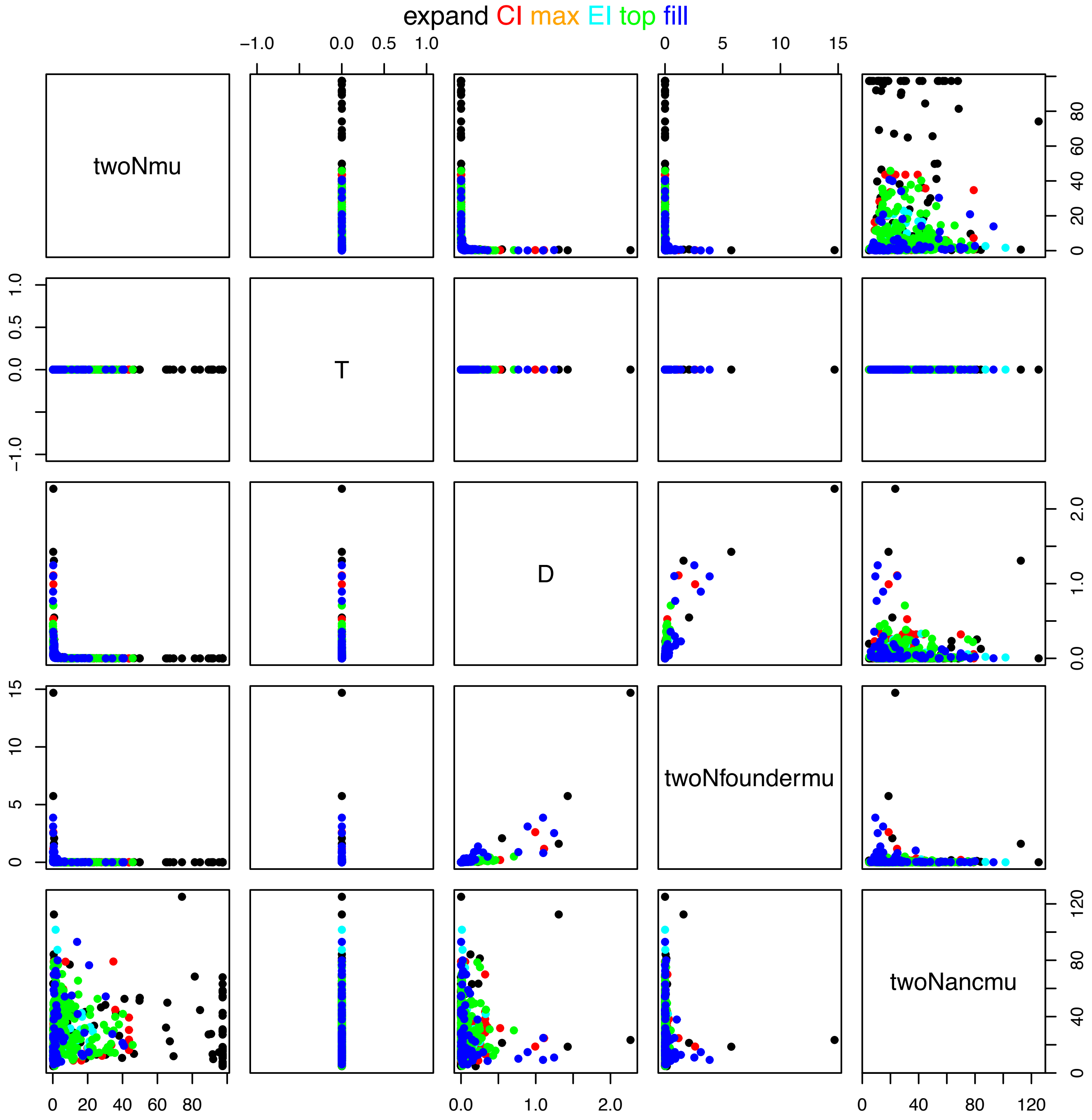

expand CI max EI top fill
twoNmu
T
D
twoNfoundermu
twoNancmu
