# Supplementary material for: Origin and dispersion pathways of guava in the Galapagos Islands inferred through genetics and historical records
Source: Ecol Evol. 2021 Oct 4;11(21):15111–31. doi: 10.1002/ece3.8193 (PMC8571588; doi:10.1002/ece3.8193)
Supplement: Supplementary file 5 — Supplementary Material [file ECE3-11-15111-s002.pdf]

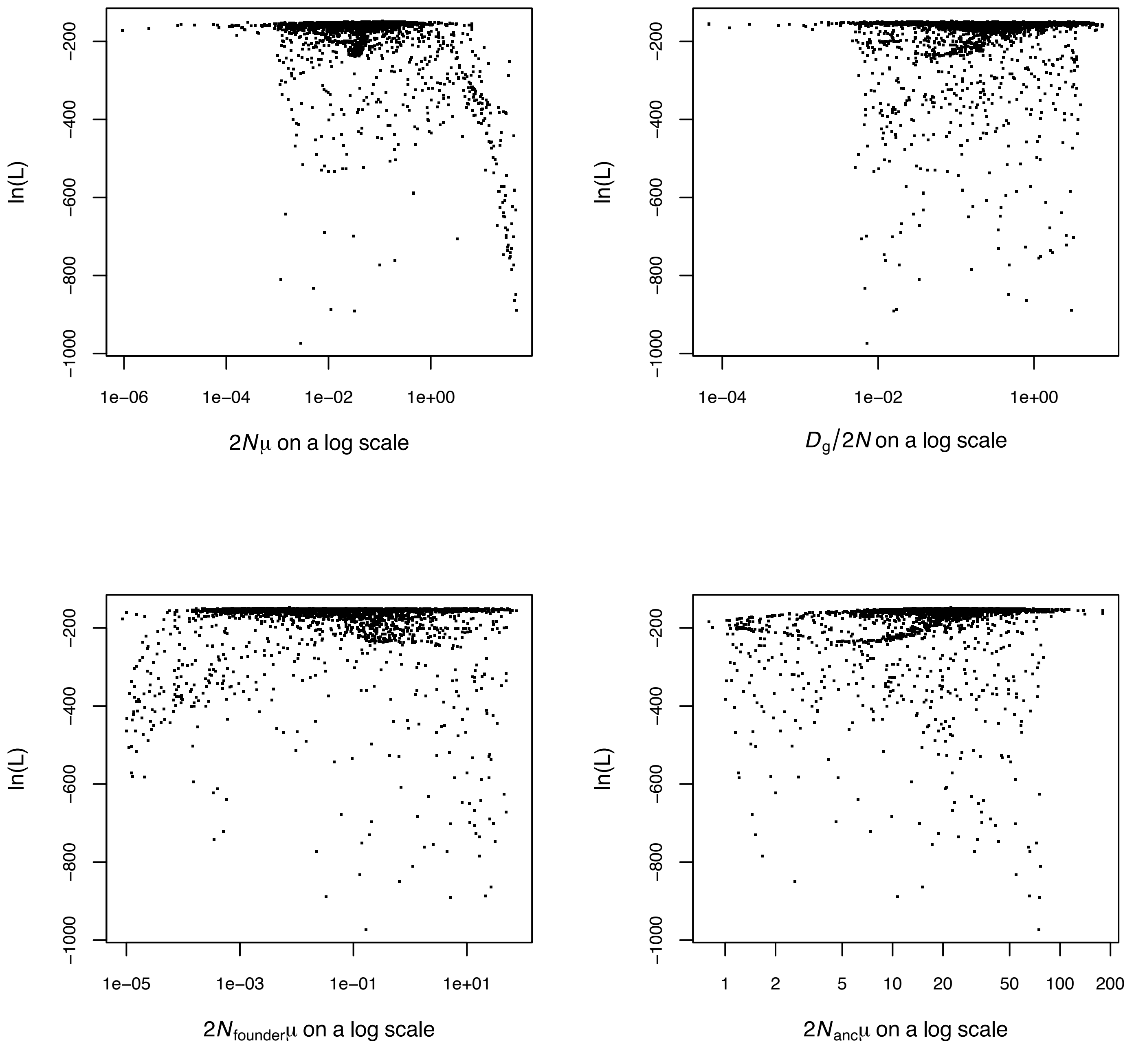
ln(L)
−200
−400
−600
−800
−1000
1e−06
1e−04
1e−02
1e+00
$2N\mu$ on a log scale
$D_g/2N$ on a log scale
1e−05
1e−03
1e−01
1e+01
$2N_{founder}\mu$ on a log scale
1
2
5
10
20
50
100
200
$2N_{anc}\mu$ on a log scale

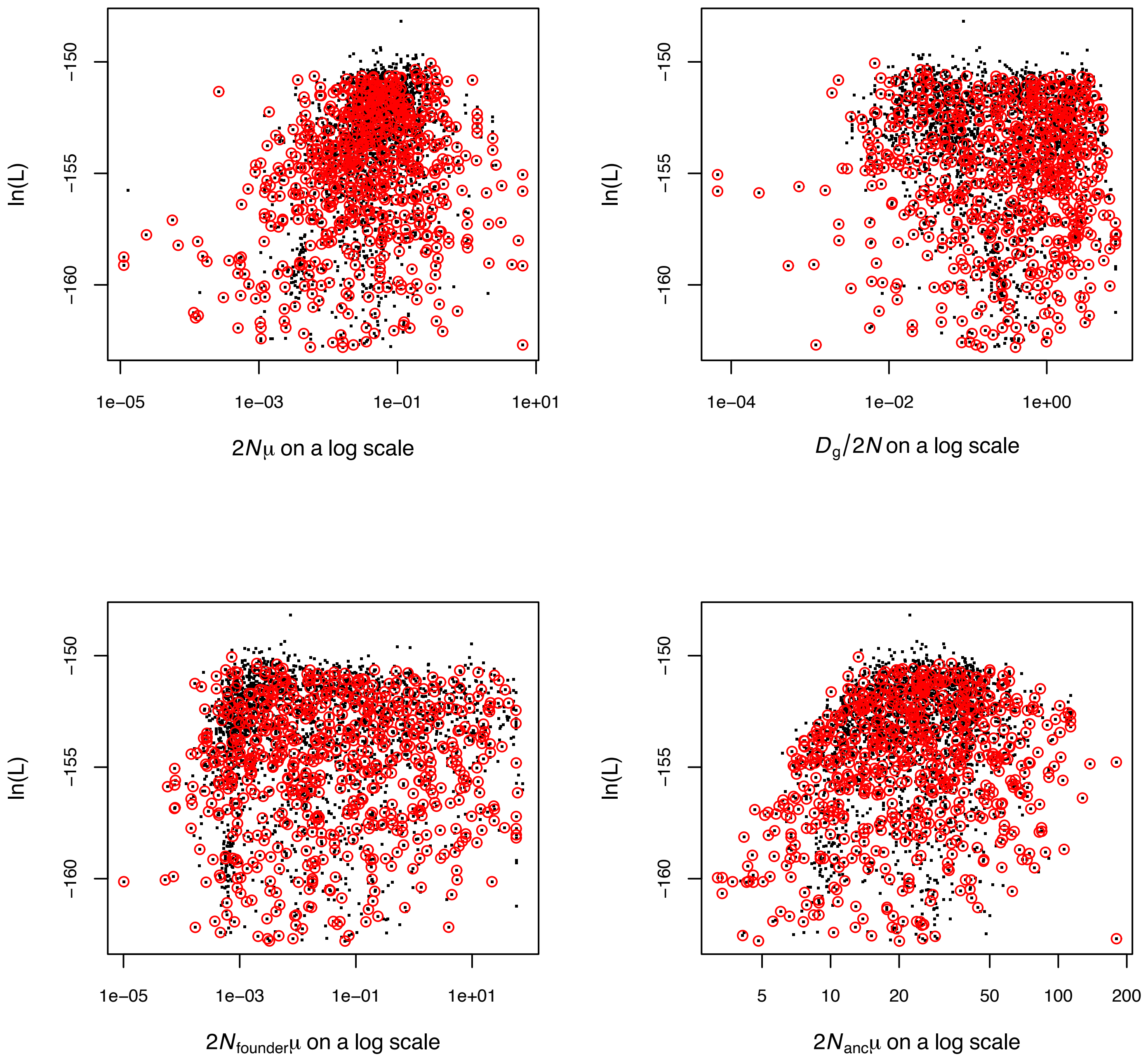

ln(L)
−150
−155
−160
1e−05
1e−03
1e−01
1e+01
2Nμ on a log scale
1e−04
1e−02
1e+00
Dg/2N on a log scale
2Nfounderμ on a log scale
5
10
20
50
100
200
2Nancμ on a log scale

One−parameter likelihood ratio profiles

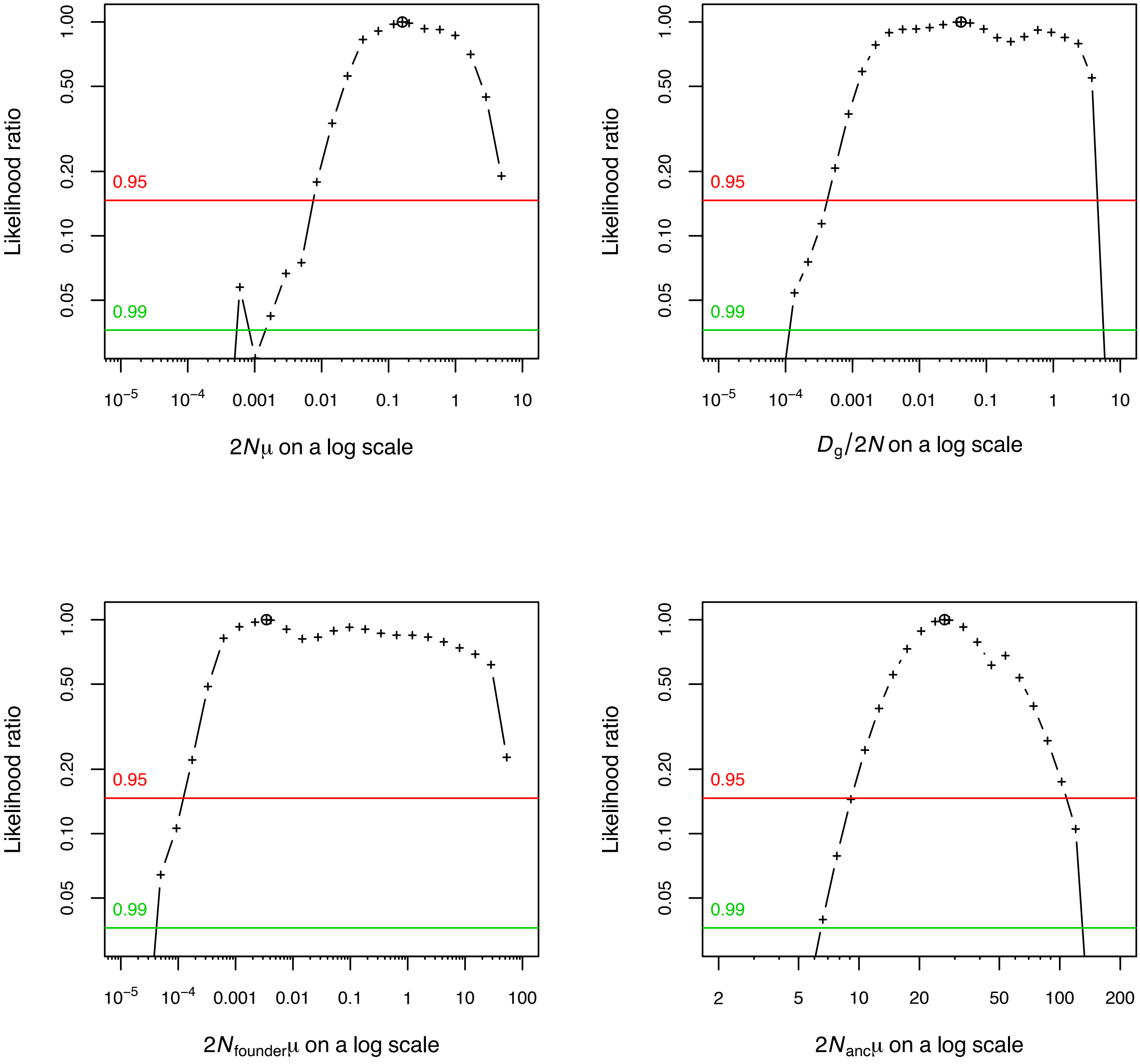

One−parameter likelihood ratio profiles

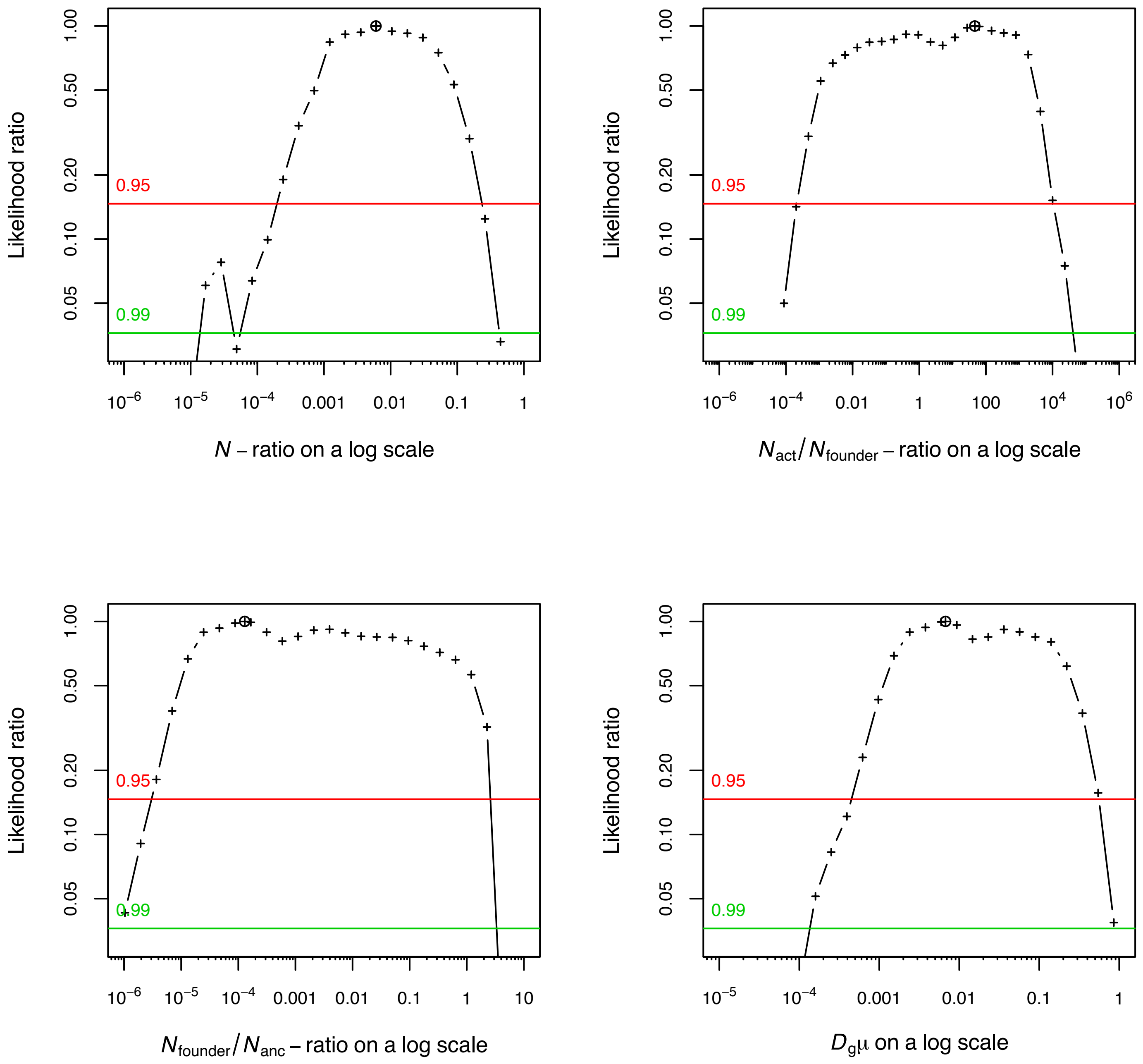

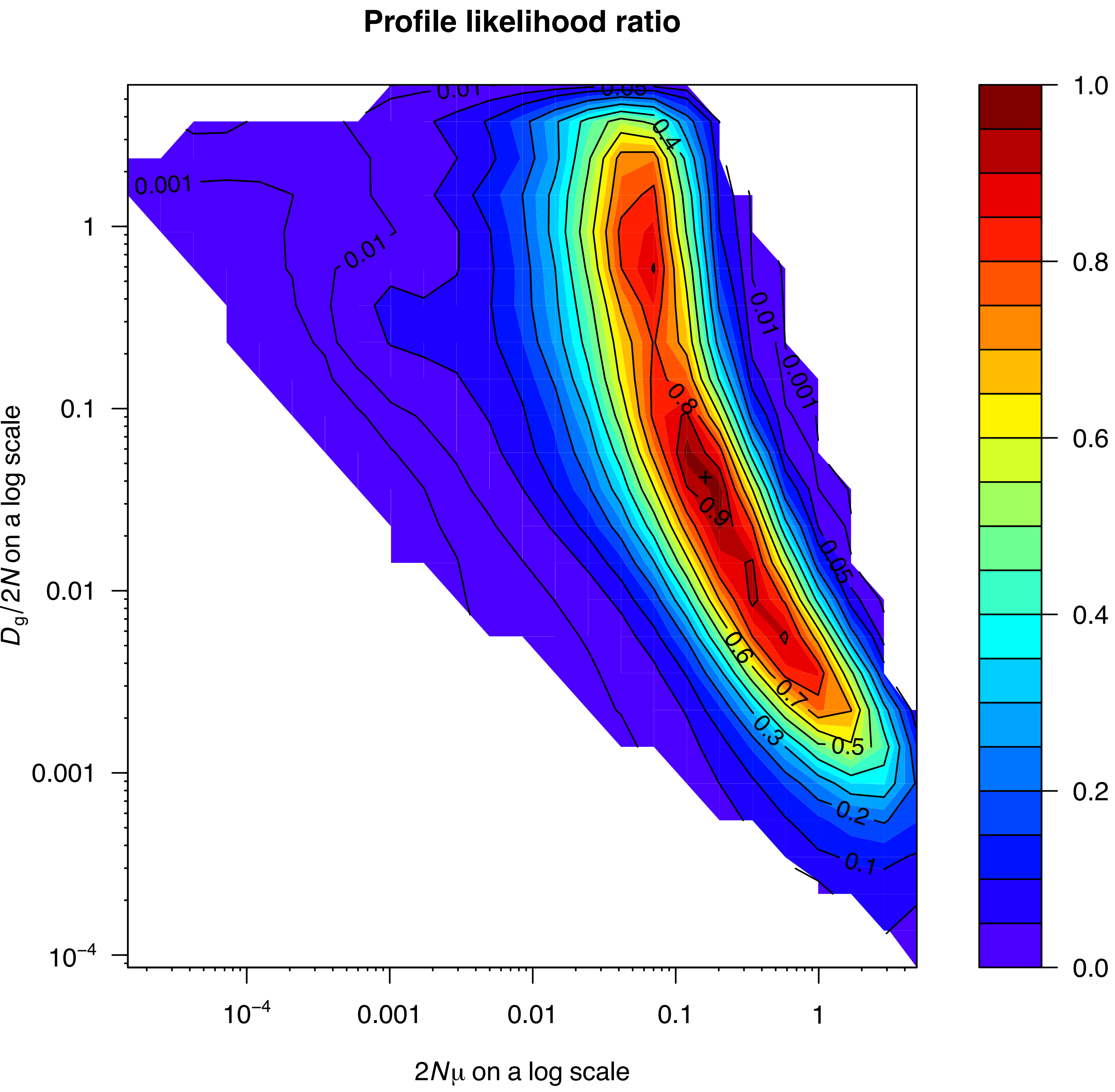

Profile likelihood ratio
$D_g/2N$ on a log scale
$2N\mu$ on a log scale
1
0.1
0.01
0.001
10^{-4}
0.001
0.01
0.1
1
0.001
0.01
0.01
0.05
0.4
0.8
0.9
0.6
0.7
0.3
0.5
0.2
0.1
0.01
0.001
0.05
1.0
0.8
0.6
0.4
0.2
0.0

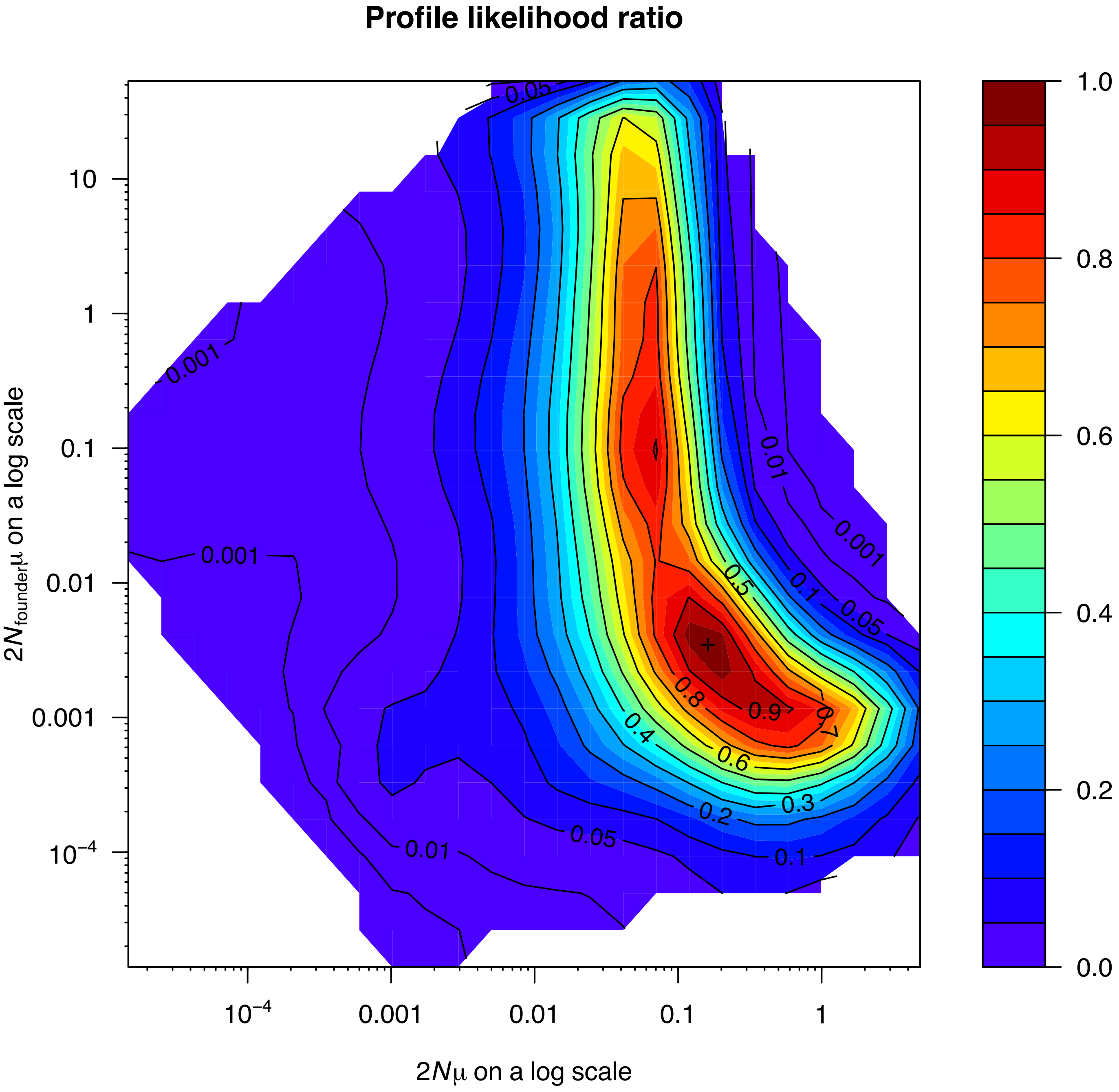

Profile likelihood ratio
2Nμ on a log scale
$2N_{founder}\mu$ on a log scale
10
1
0.1
0.01
0.001
10^{-4}
0.001
0.01
0.1
1
1.0
0.8
0.6
0.4
0.2
0.0
0.05
0.001
0.001
0.01
0.001
0.1
0.05
0.5
0.8
0.9
0.7
0.4
0.6
0.3
0.2
0.05
0.01
0.1

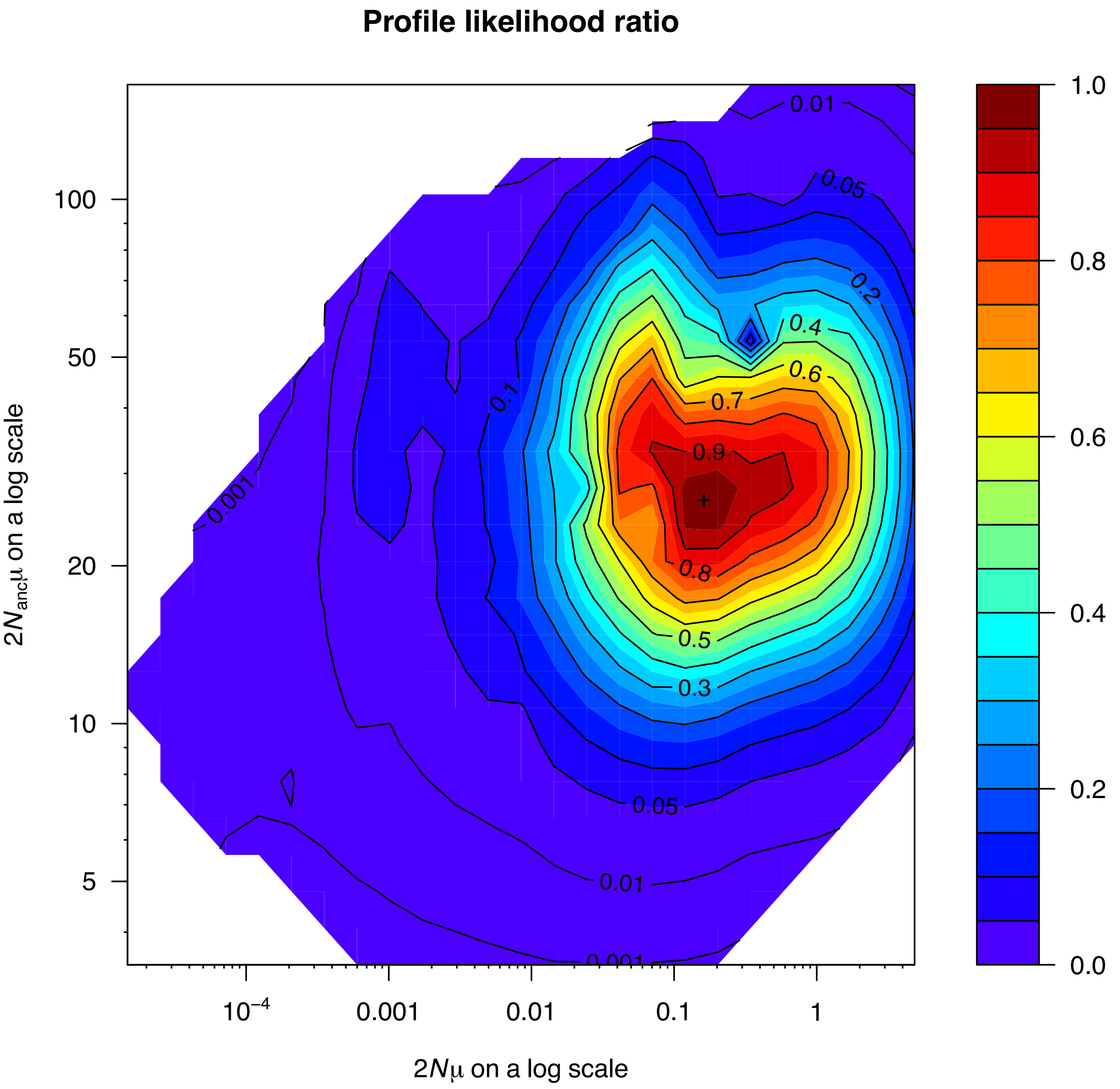

Profile likelihood ratio
$2N_{anc}\mu$ on a log scale
$2N\mu$ on a log scale
100
50
20
10
5
10^{-4}
0.001
0.01
0.1
1
0.01
0.05
0.2
0.4
0.6
0.7
0.9
0.8
0.5
0.3
0.1
0.001
1.0
0.8
0.6
0.4
0.2
0.0

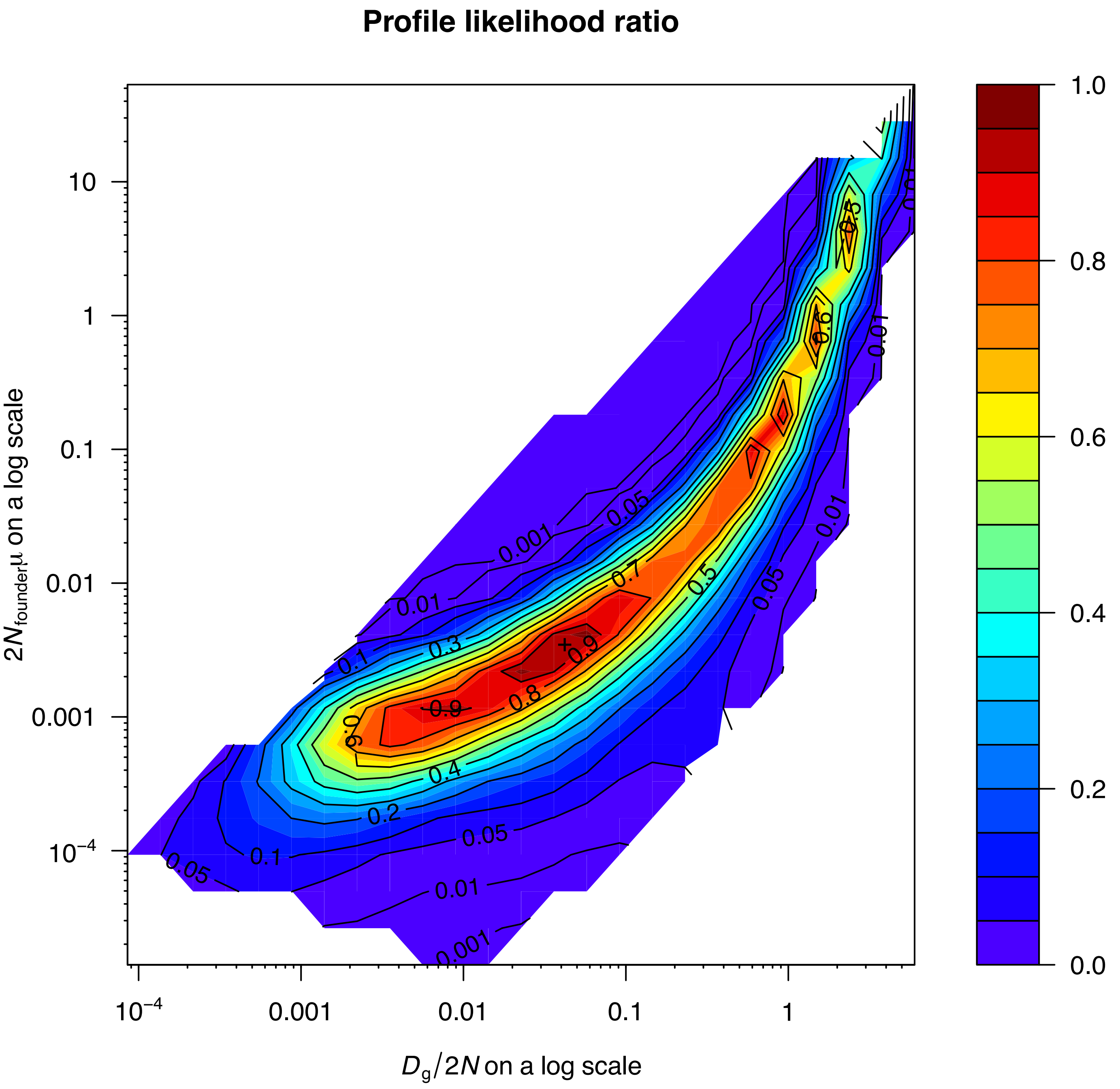

Profile likelihood ratio
$2N_{\text{founder}}\mu$ on a log scale
$D_g/2N$ on a log scale
10
1
0.1
0.01
0.001
10^{-4}
0.001
0.01
0.1
1
1.0
0.8
0.6
0.4
0.2
0.0

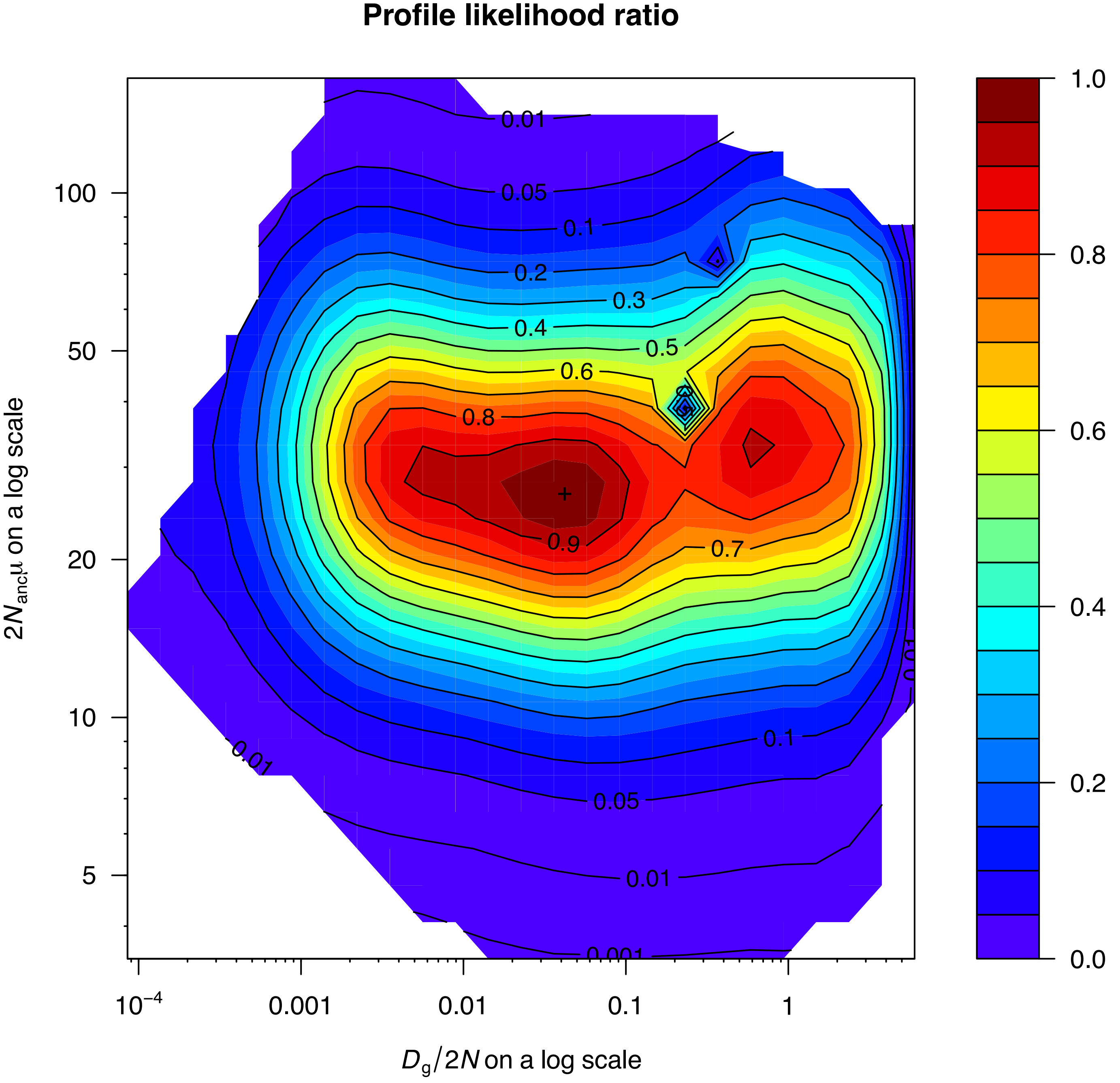
Profile likelihood ratio
0.01
0.05
0.1
0.2
0.3
0.4
0.5
0.6
0.8
0.9
0.7
0.1
0.05
0.01
0.01
0.001
0.01
100
50
20
10
5
$2N_{anc}\mu$ on a log scale
10^{-4}
0.001
0.01
0.1
1
$D_g/2N$ on a log scale
1.0
0.8
0.6
0.4
0.2
0.0

Profile likelihood ratio

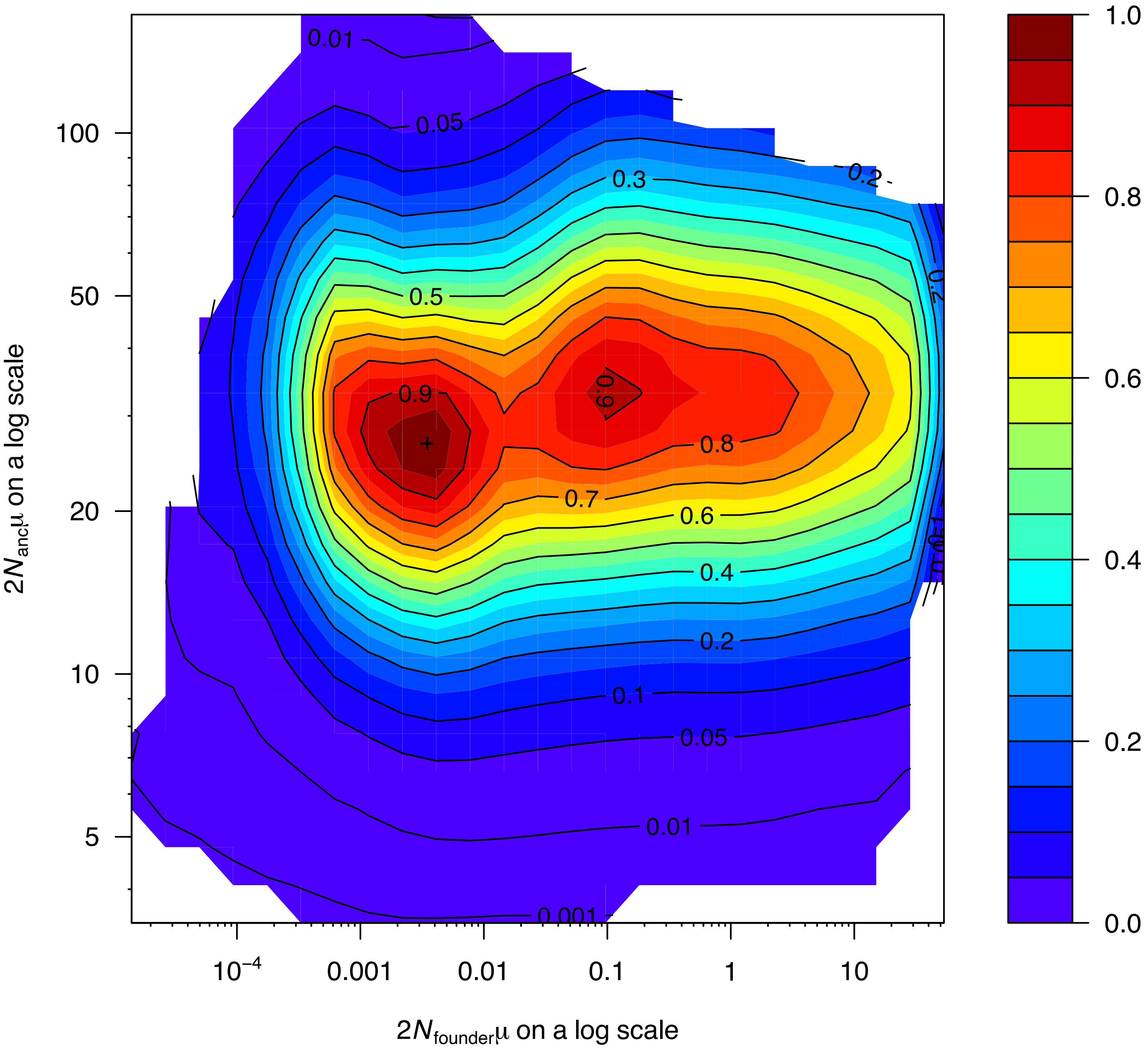

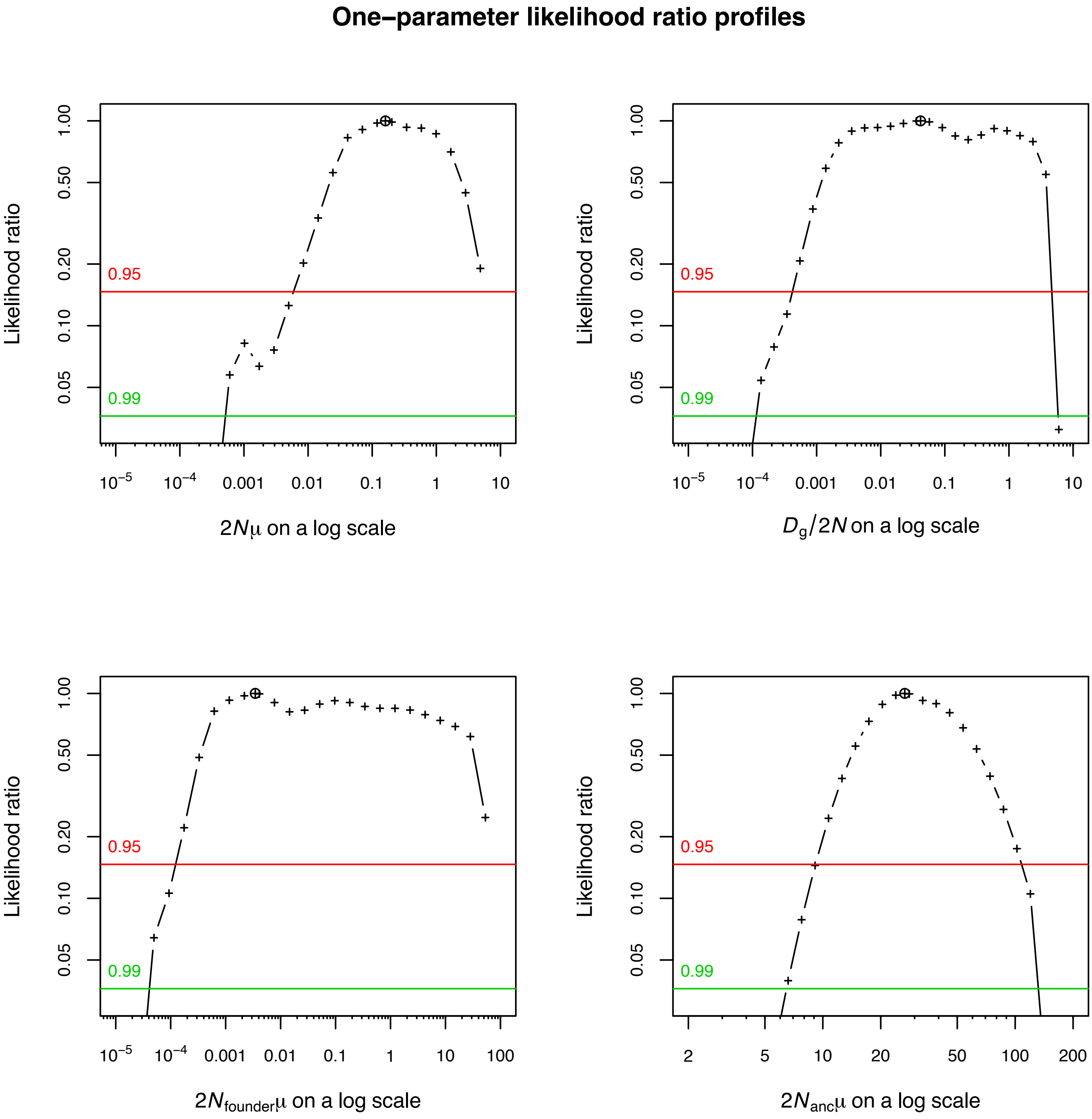

One–parameter likelihood ratio profiles
Likelihood ratio
0.95
0.99
1.00
0.50
0.20
0.10
0.05
10^−5
10^−4
0.001
0.01
0.1
1
10
100
2
5
20
50
200
2Nμ on a log scale
D_g/2N on a log scale
2N_founder μ on a log scale
2N_anc μ on a log scale

One–parameter likelihood ratio profiles

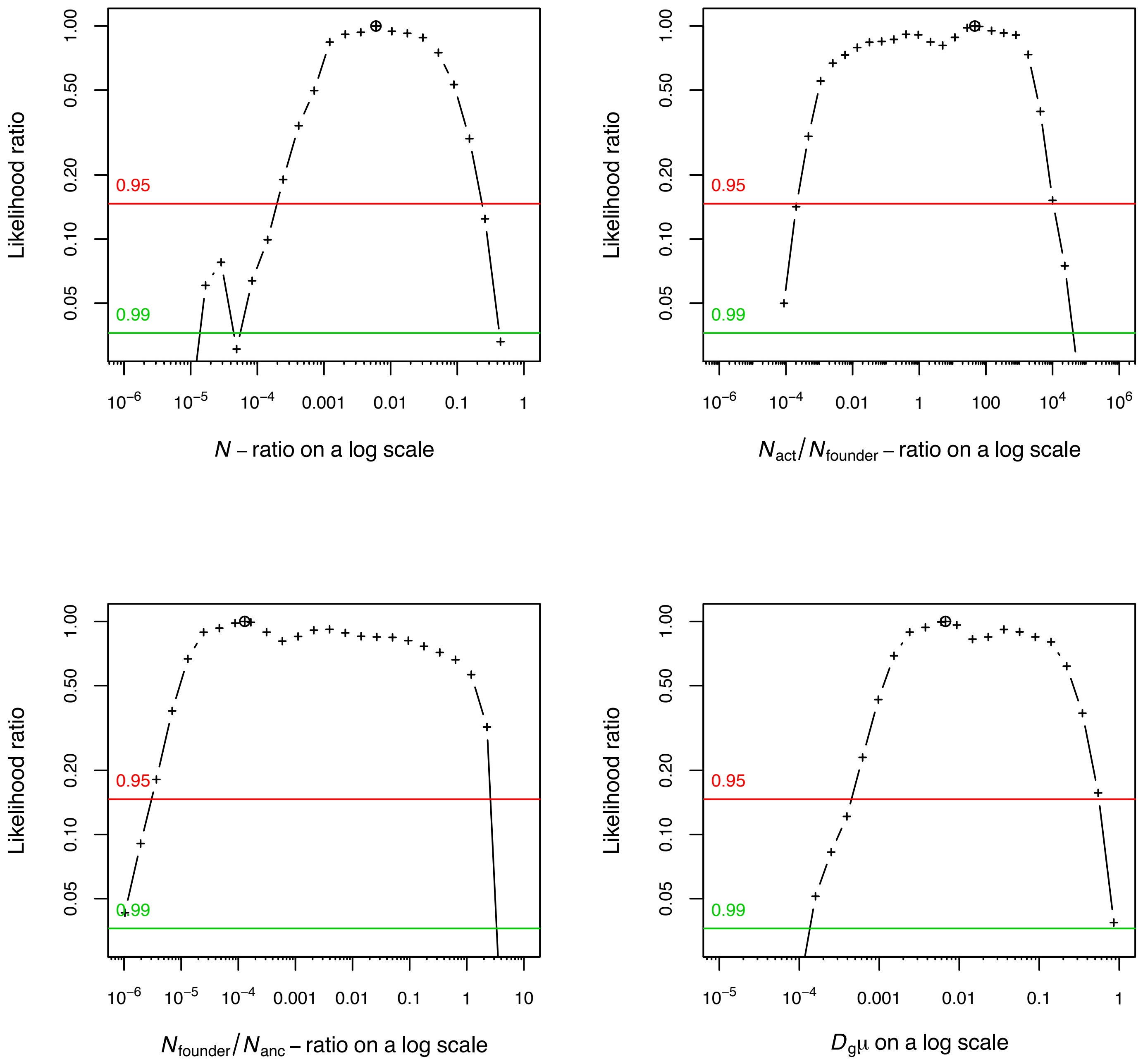

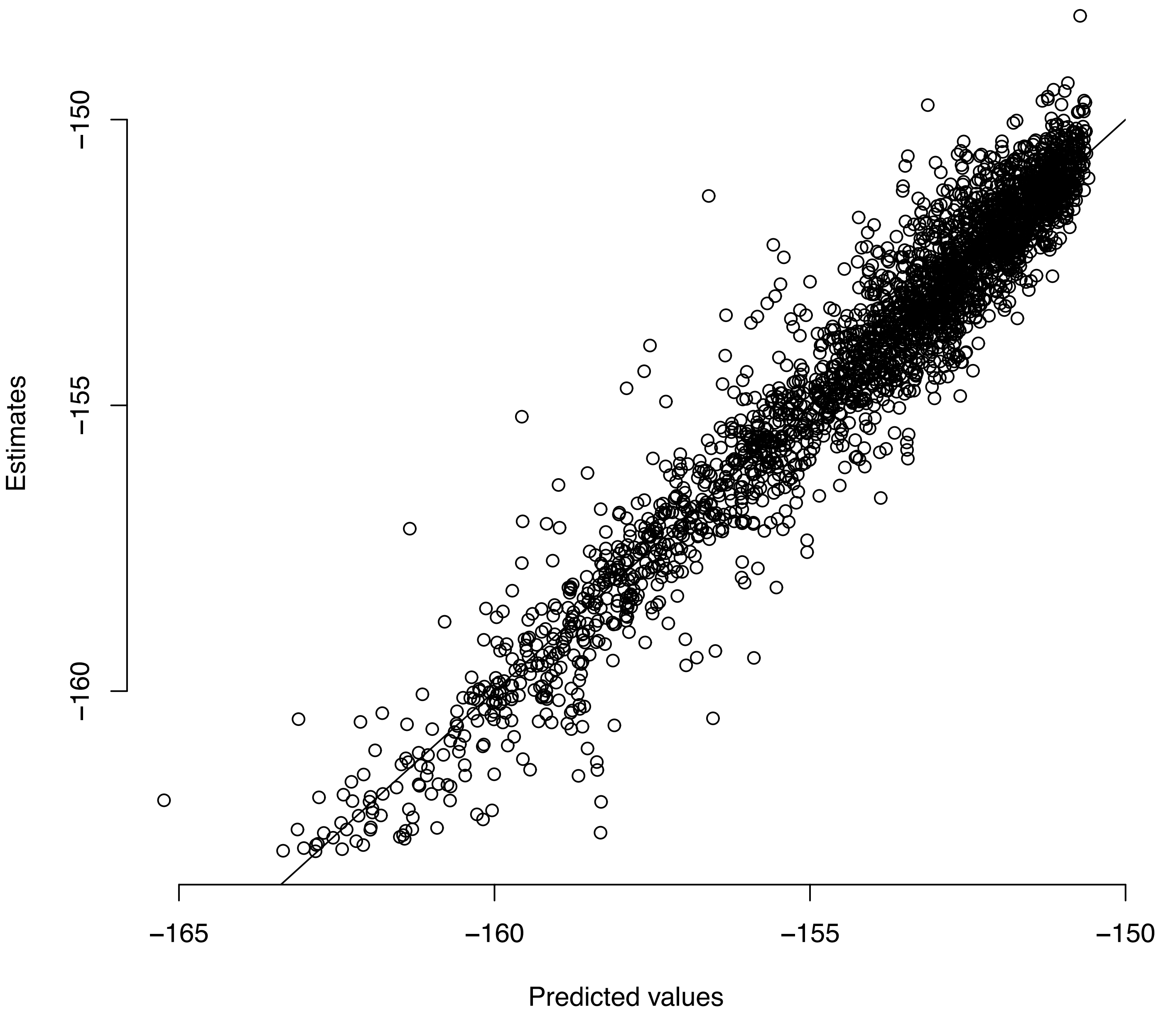
Estimates
−150
−155
−160
−165
−160
−155
−150
Predicted values

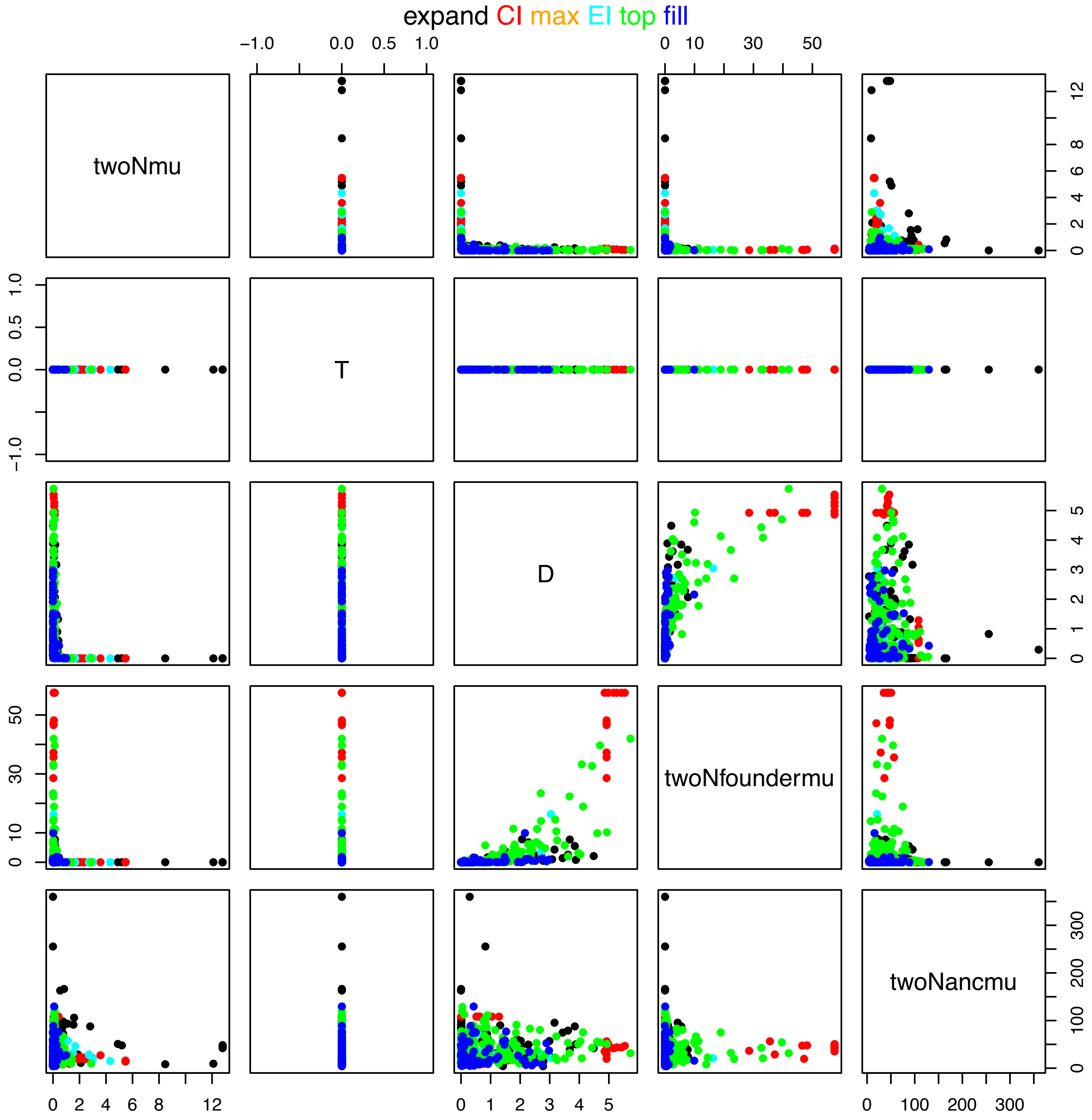

expand CI max EI top fill
twoNmu
T
D
twoNfoundermu
twoNancmu
−1.0 0.0 0.5 1.0
0 10 30 50
0 2 4 6 8 12
0.0 0.5 1.0
0 1 2 3 4 5
0 10 30 50
0 100 200 300
