# Supplementary material for: Origin and dispersion pathways of guava in the Galapagos Islands inferred through genetics and historical records
Source: Ecol Evol. 2021 Oct 4;11(21):15111–31. doi: 10.1002/ece3.8193 (PMC8571588; doi:10.1002/ece3.8193)
Supplement: Supplementary file 6 — Supplementary Material [file ECE3-11-15111-s007.pdf]

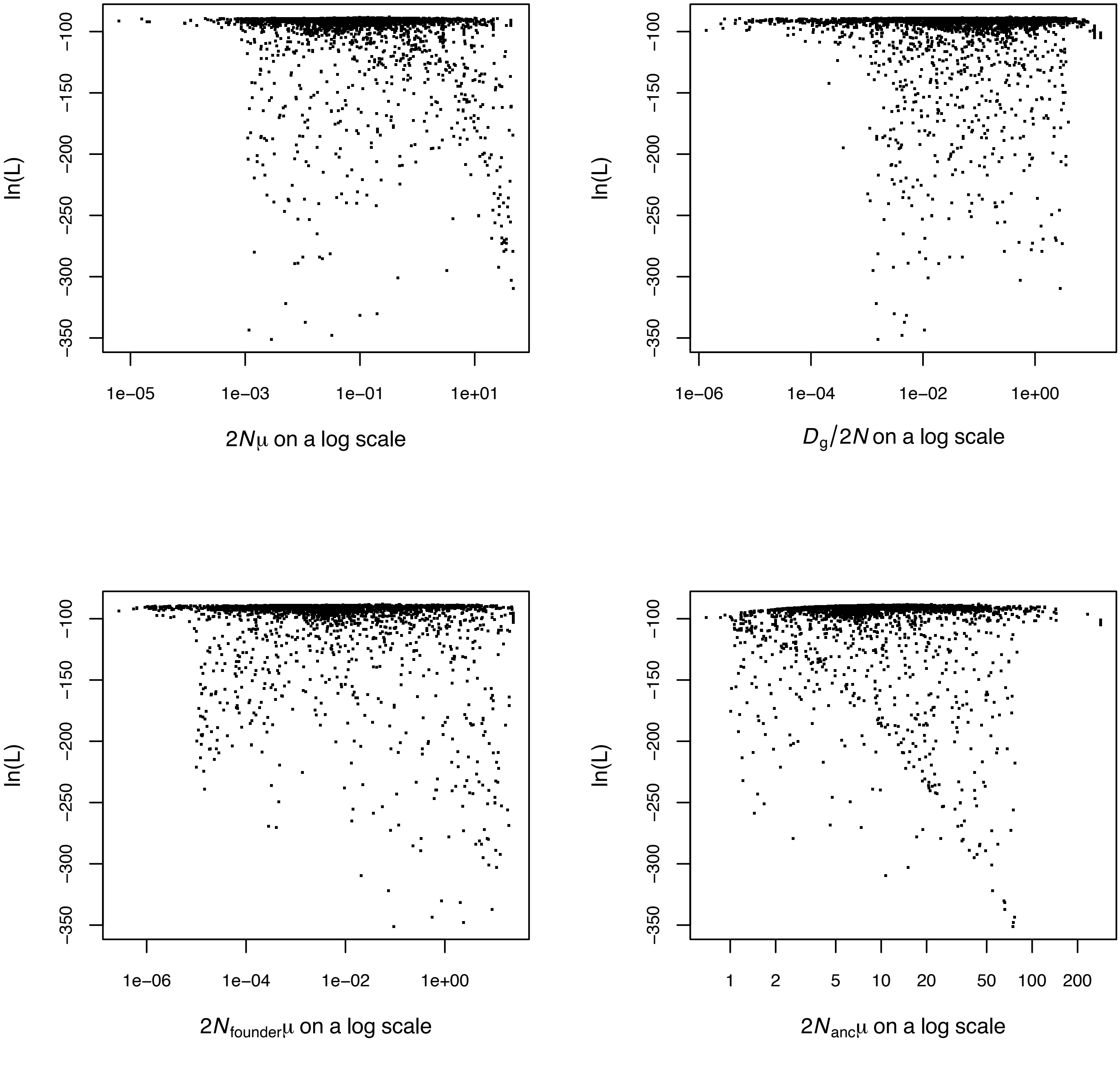
ln(L)
−100
−150
−200
−250
−300
−350
1e−05
1e−03
1e−01
1e+01
2Nμ on a log scale
ln(L)
1e−06
1e−04
1e−02
1e+00
Dg/2N on a log scale
ln(L)
1e−06
1e−04
1e−02
1e+00
2Nfounderμ on a log scale
ln(L)
1
2
5
10
20
50
100
200
2Nancμ on a log scale

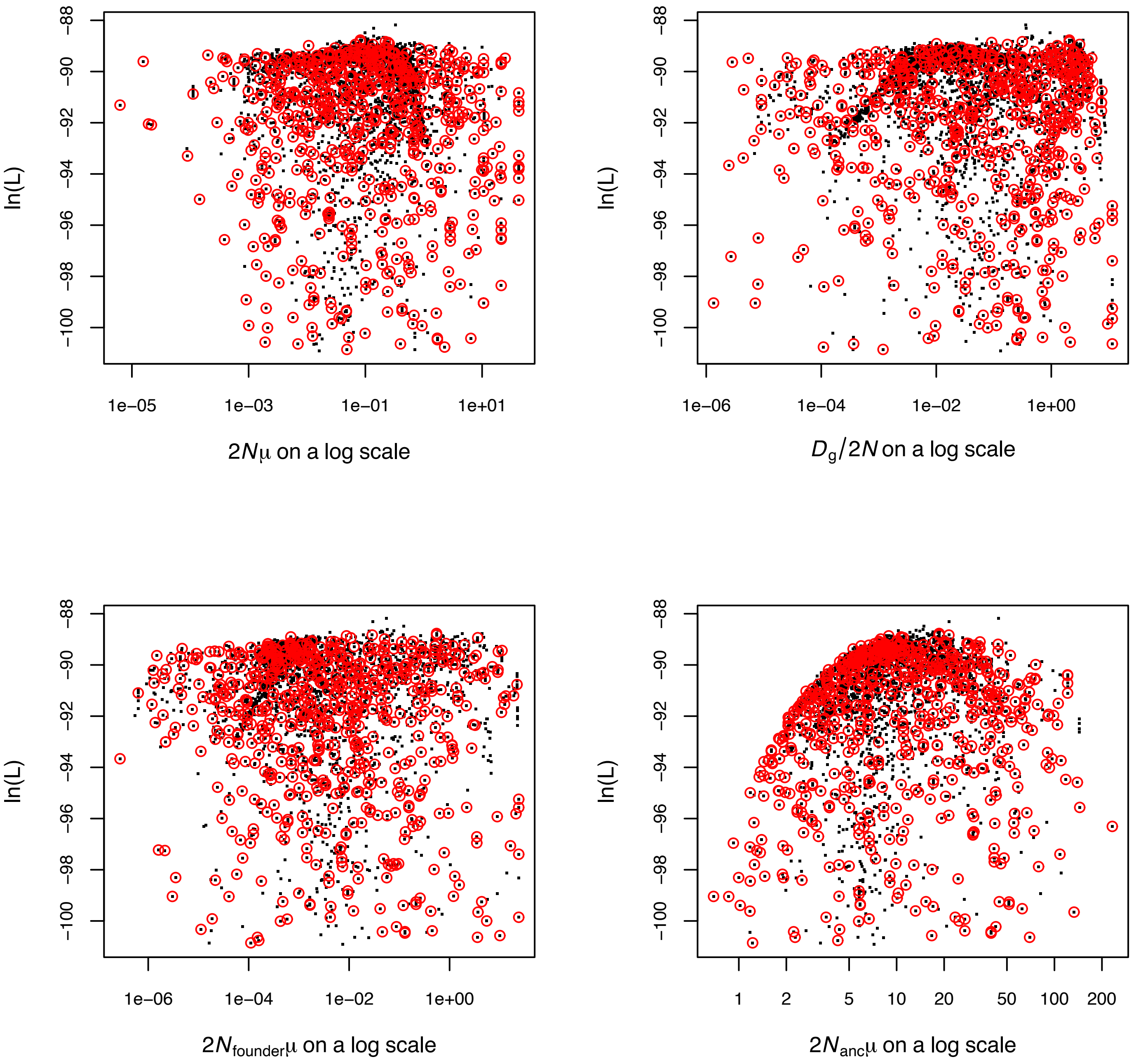

-88
-90
-92
-94
-96
-98
-100
ln(L)
1e-05
1e-03
1e-01
1e+01
2Nμ on a log scale
1e-06
1e-04
1e-02
1e+00
D_g/2N on a log scale
2N_founder μ on a log scale
1
2
5
10
20
50
100
200
2N_anc μ on a log scale

One–parameter likelihood ratio profiles

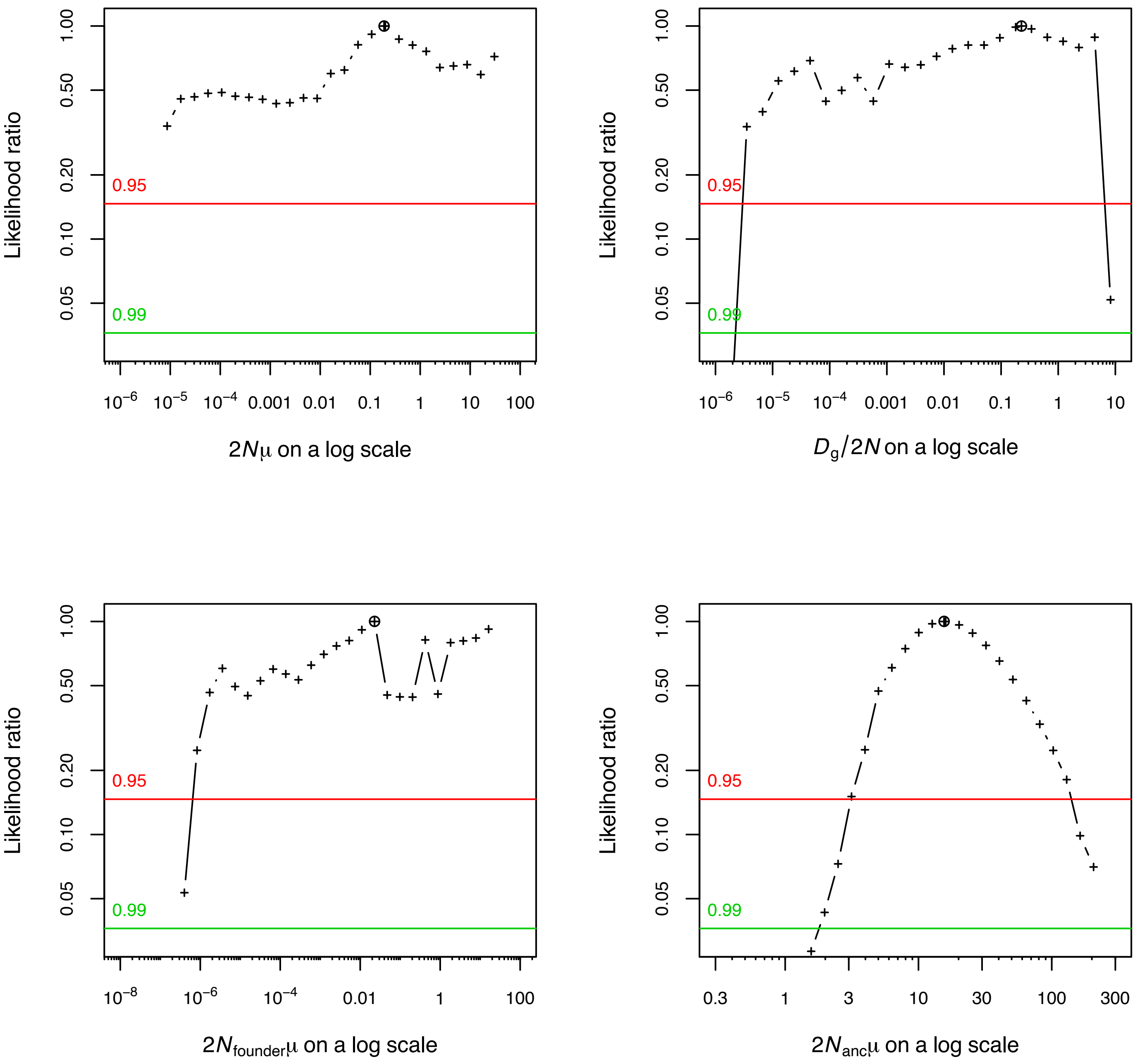

One–parameter likelihood ratio profiles

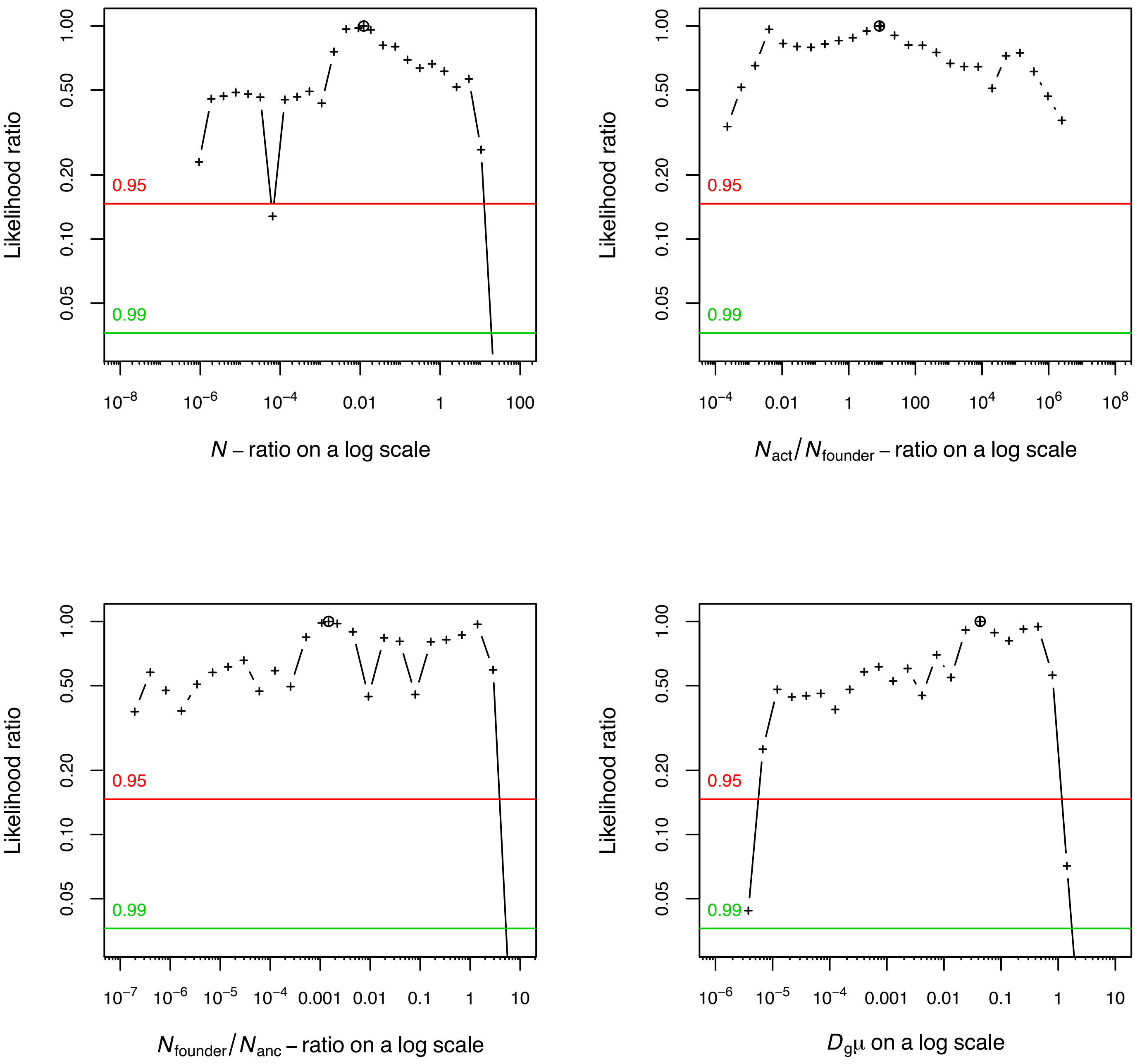

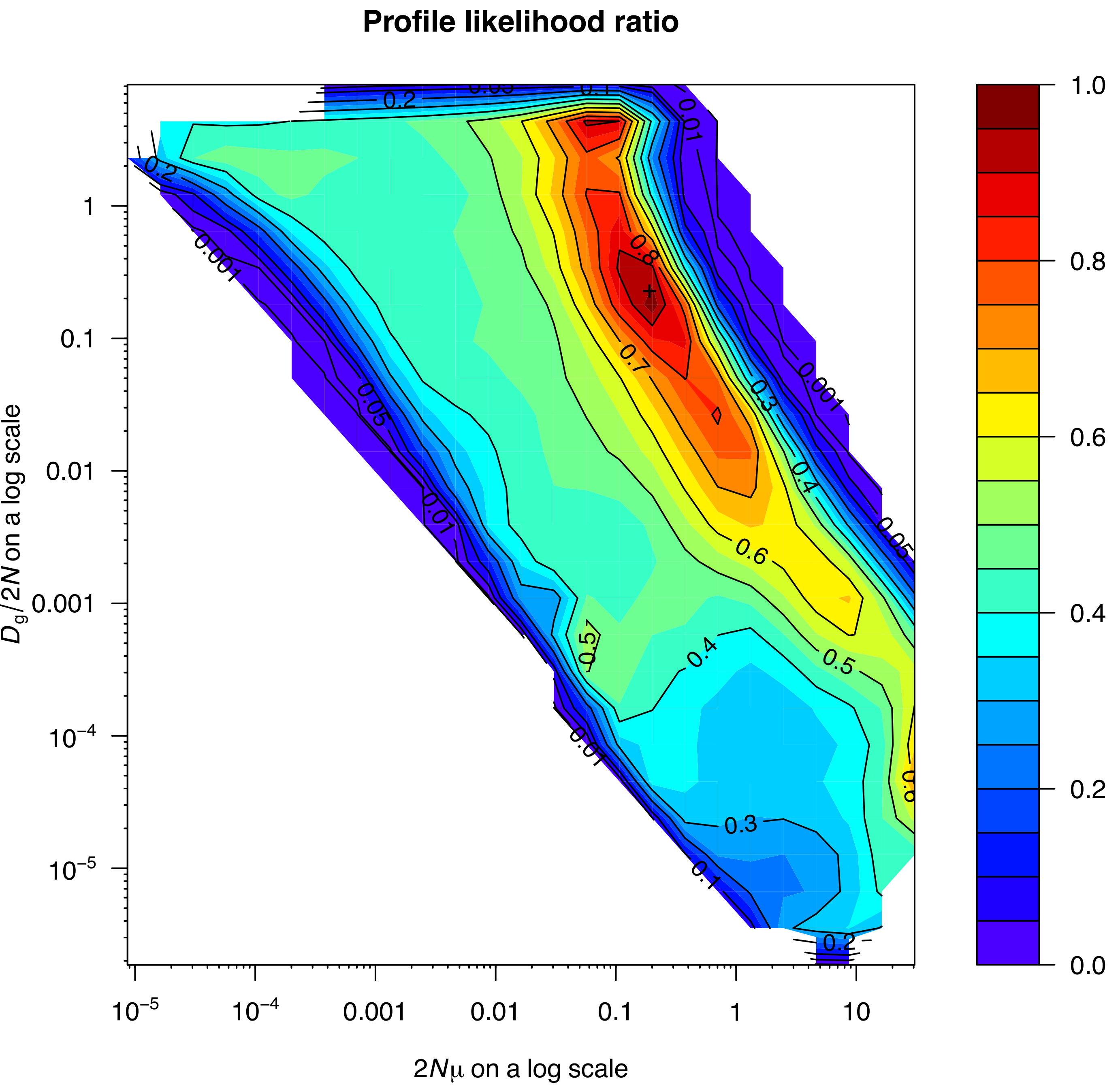

Profile likelihood ratio
$D_g/2N$ on a log scale
$2N\mu$ on a log scale
1
0.1
0.01
0.001
10^{-4}
10^{-5}
0.001
0.01
0.1
1
10
1.0
0.8
0.6
0.4
0.2
0.0
0.2
0.001
0.05
0.01
0.8
0.7
0.6
0.5
0.4
0.3
0.1
0.001
0.05

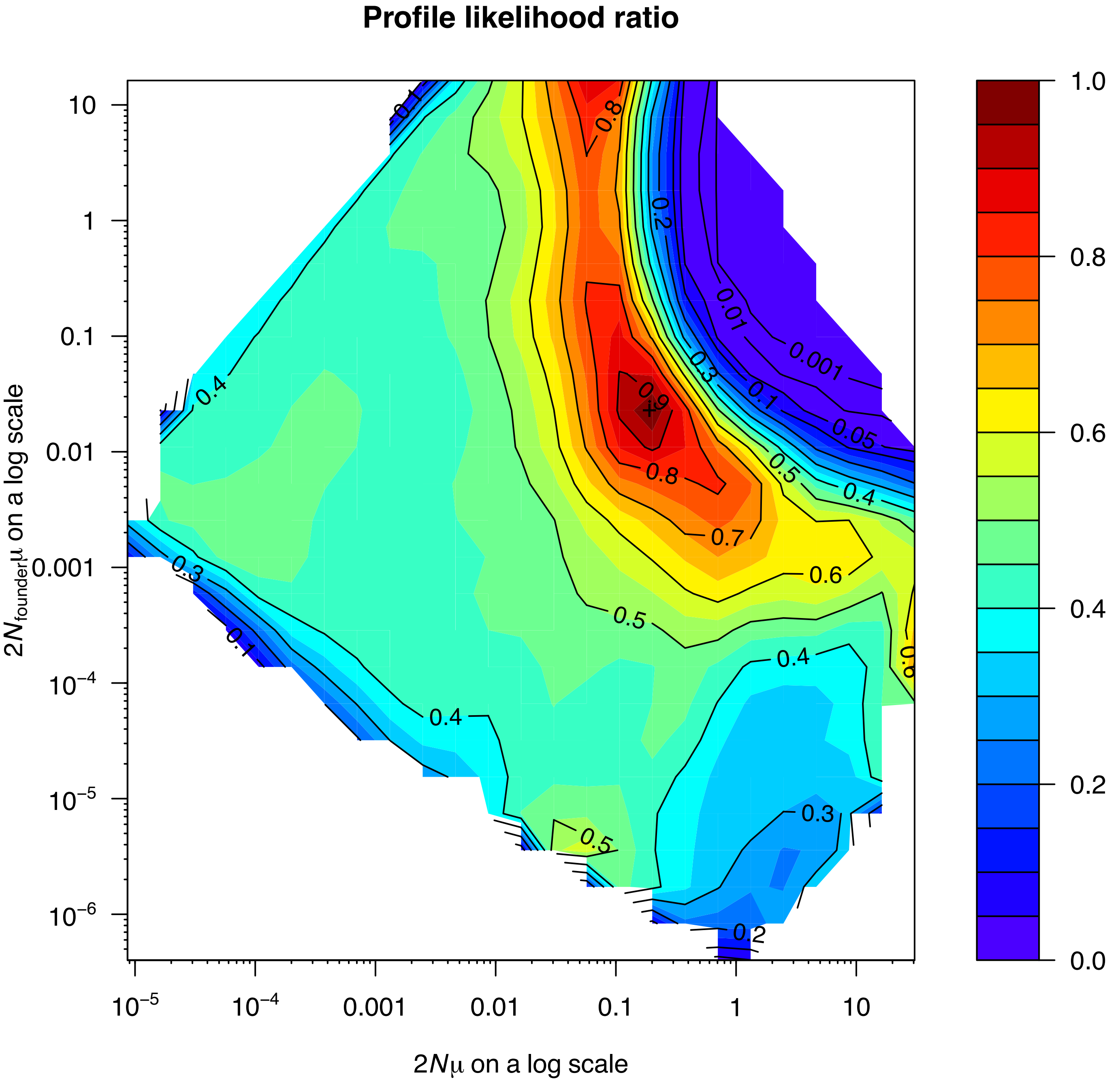

Profile likelihood ratio
$2N_{founder}\mu$ on a log scale
$2N\mu$ on a log scale
10
1
0.1
0.01
0.001
10^{-4}
10^{-5}
10^{-6}
0.001
0.01
0.1
1
10
10^{-5}
10^{-4}
1.0
0.8
0.6
0.4
0.2
0.0
0.8
0.9
0.7
0.6
0.5
0.4
0.3
0.2
0.1
0.05
0.01
0.001

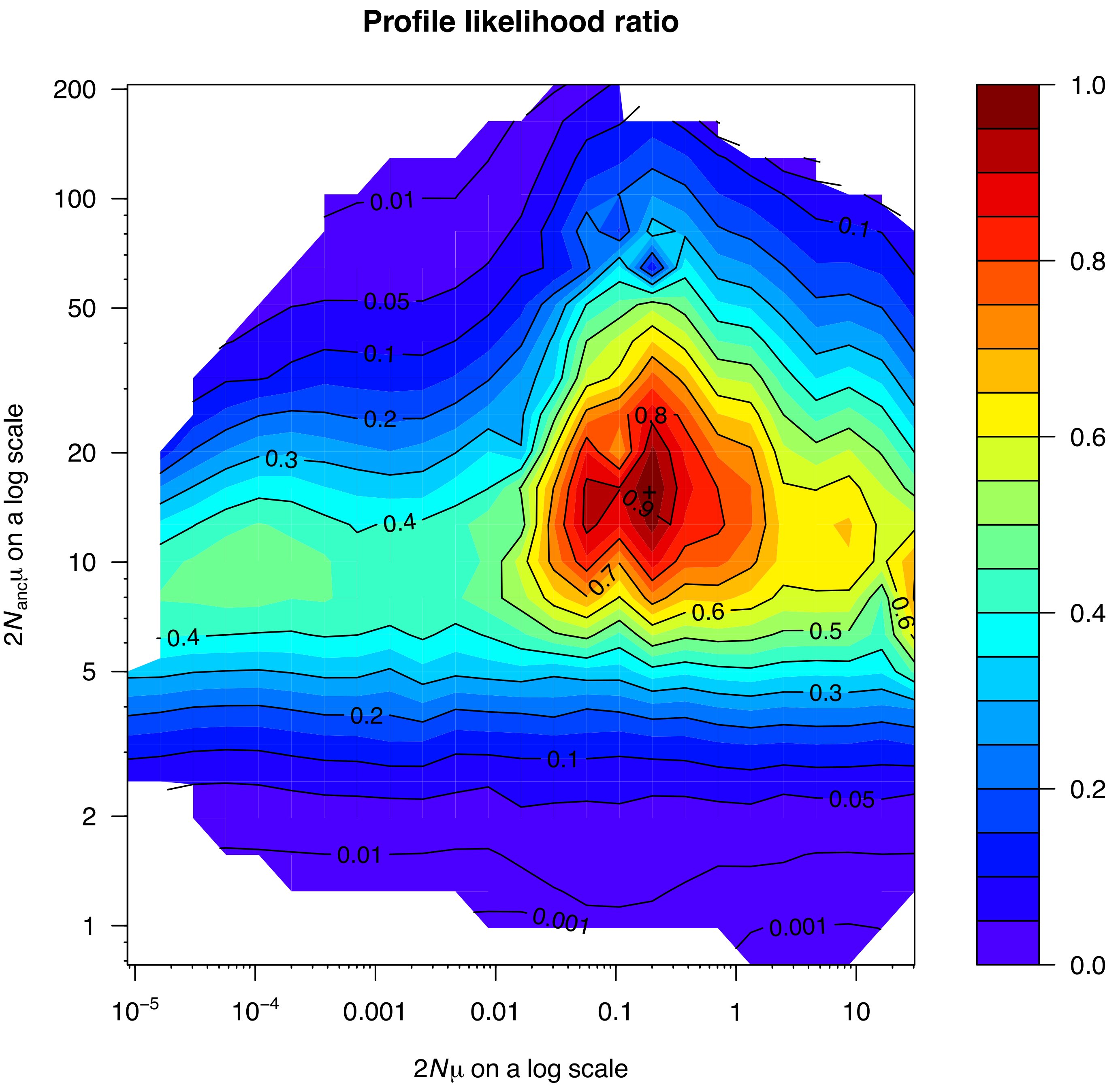
Profile likelihood ratio
200
100
50
20
10
5
2
1
$2N_{anc}\mu$ on a log scale
10^{-5}
10^{-4}
0.001
0.01
0.1
1
10
$2N\mu$ on a log scale
0.01
0.05
0.1
0.2
0.3
0.4
0.5
0.6
0.7
0.8
0.9
0.001
1.0
0.8
0.6
0.4
0.2
0.0

Profile likelihood ratio

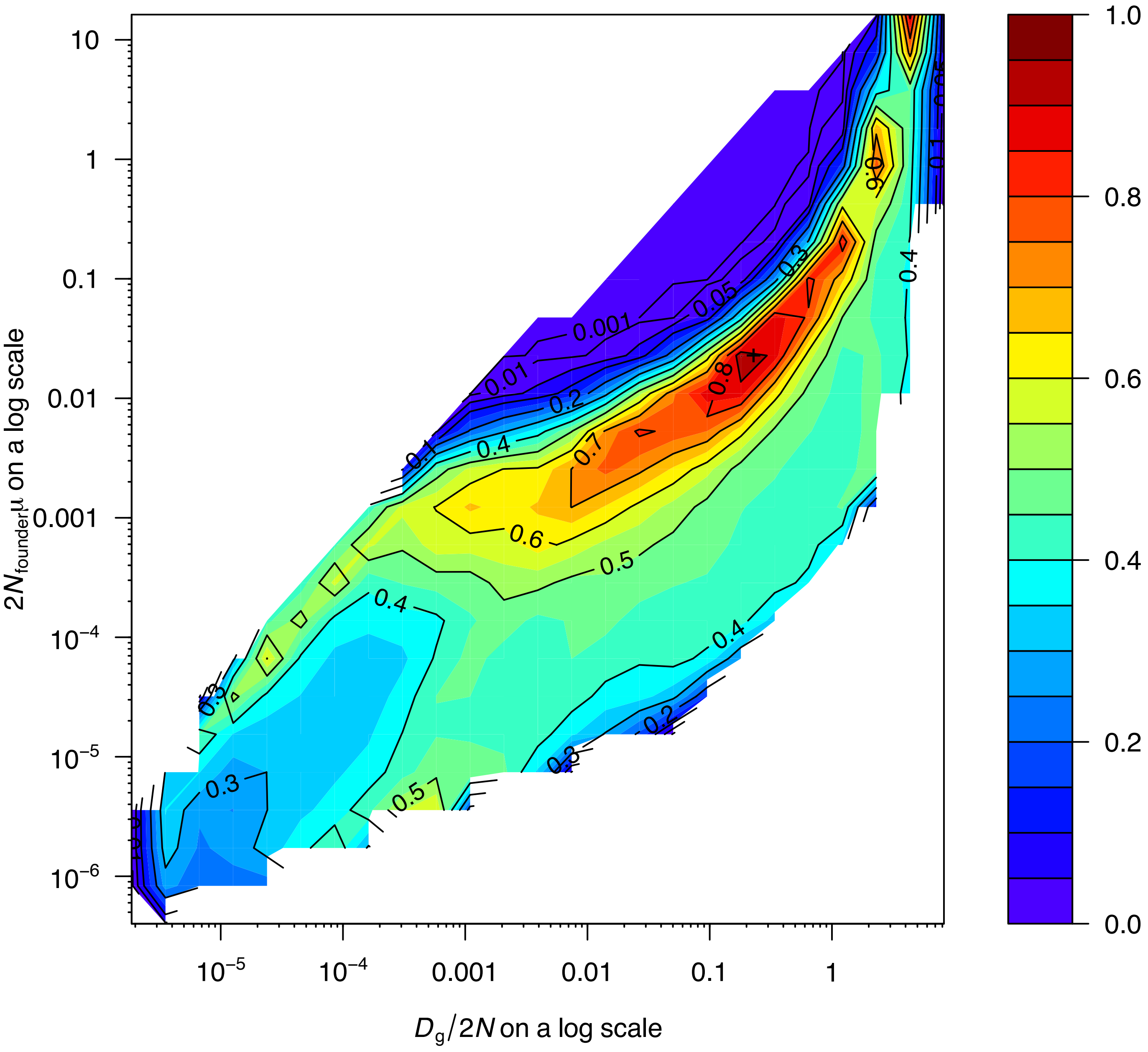

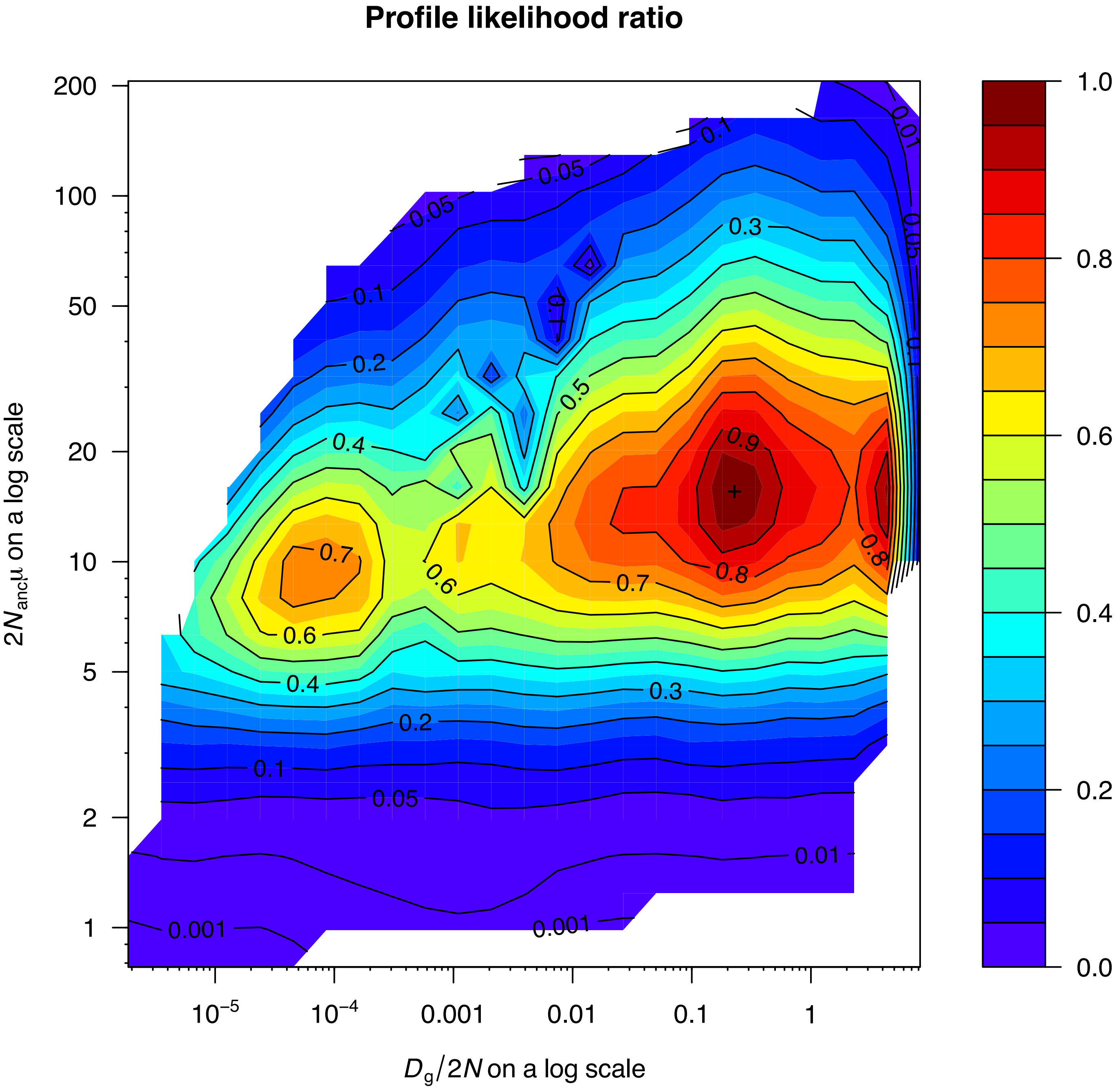

Profile likelihood ratio
200
100
50
20
10
5
2
1
10^{-5}
10^{-4}
0.001
0.01
0.1
1
$2N_{\text{anc}}\mu$ on a log scale
$D_g/2N$ on a log scale
1.0
0.8
0.6
0.4
0.2
0.0
0.001
0.01
0.05
0.1
0.2
0.3
0.4
0.5
0.6
0.7
0.8
0.9

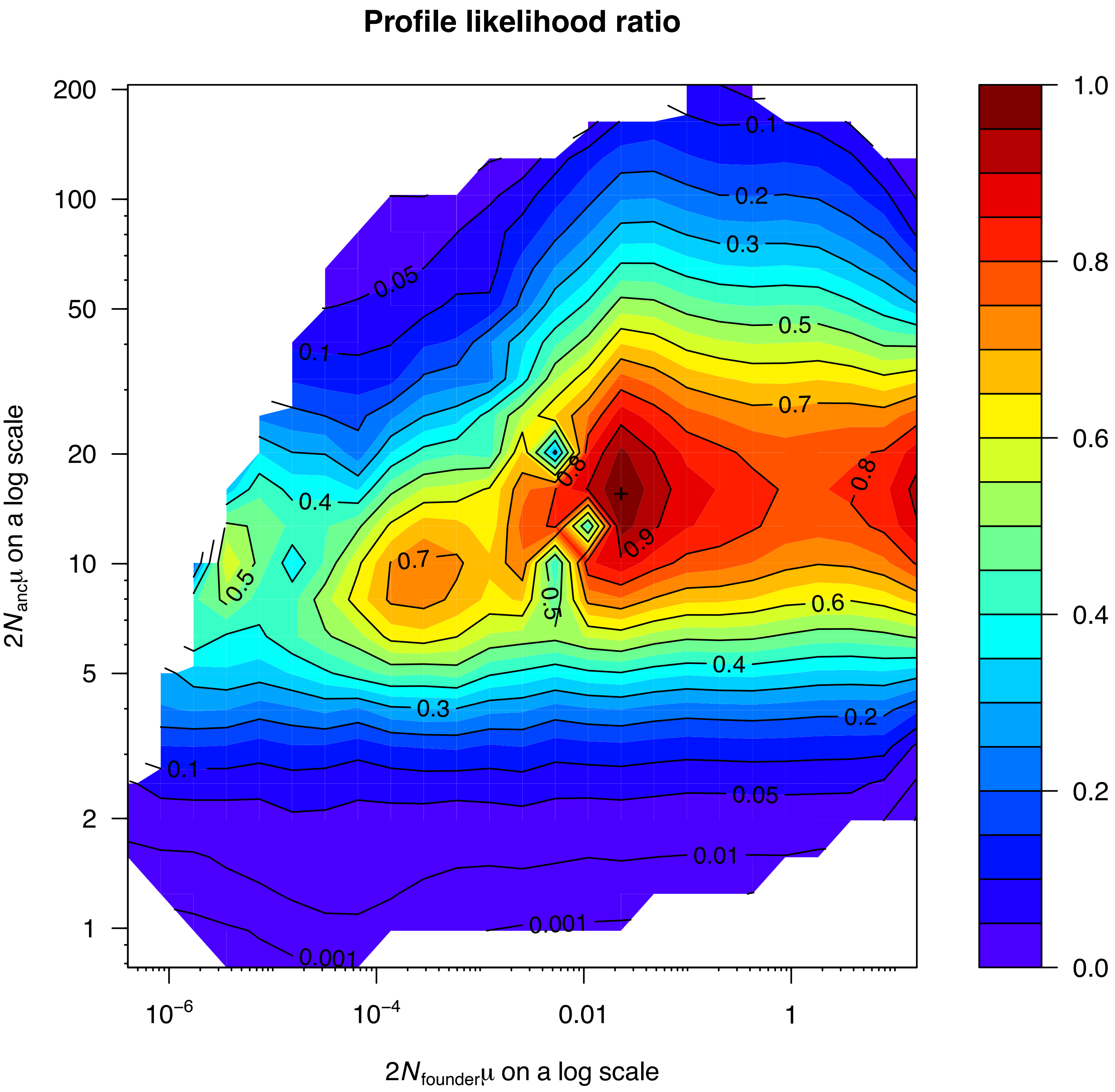

Profile likelihood ratio
200
100
50
20
10
5
2
1
10^−6
10^−4
0.01
1
2N_ancμ on a log scale
2N_founderμ on a log scale
0.1
0.2
0.3
0.5
0.7
0.8
0.9
0.6
0.4
0.05
0.01
0.001
1.0
0.0

One−parameter likelihood ratio profiles

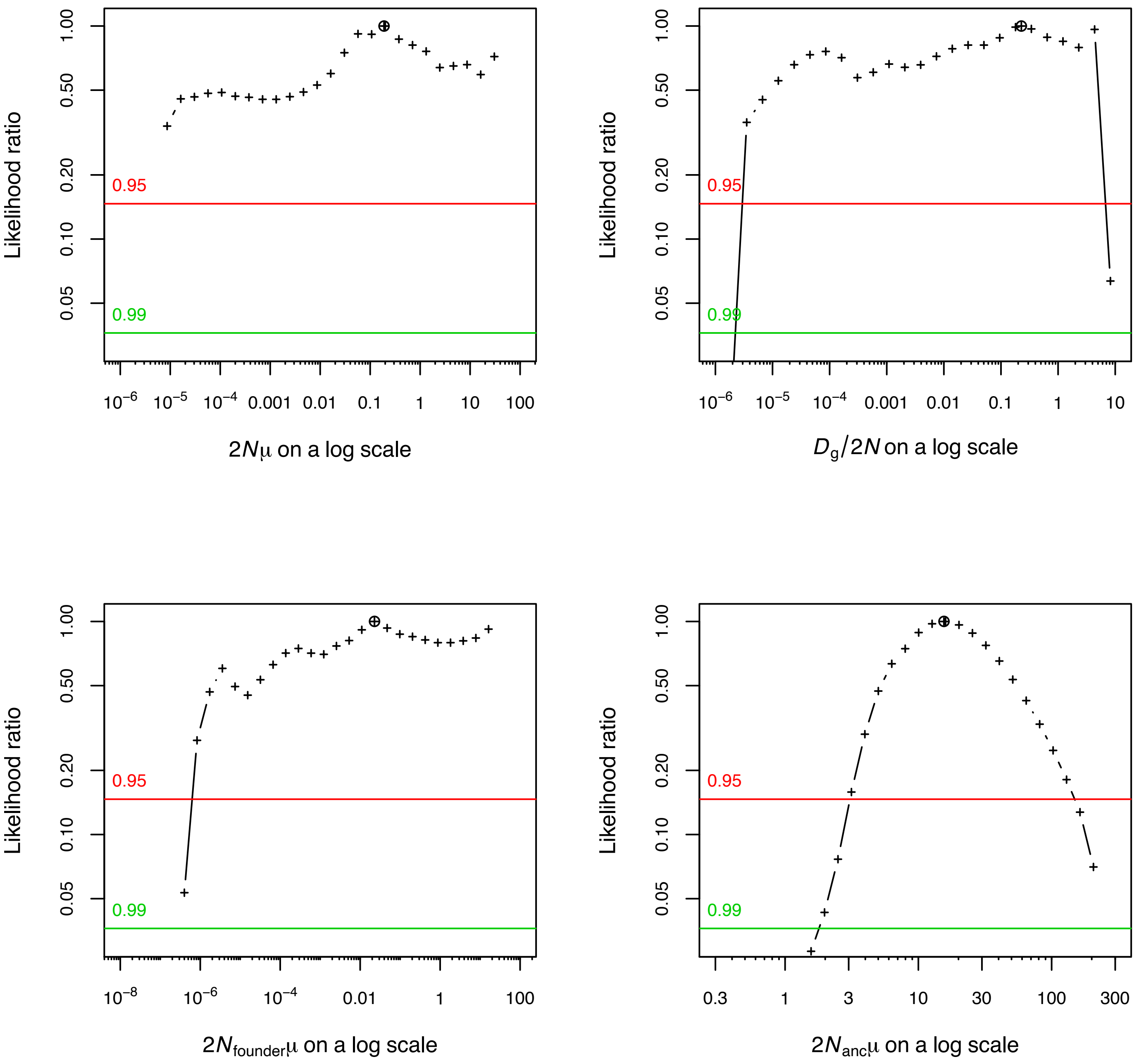

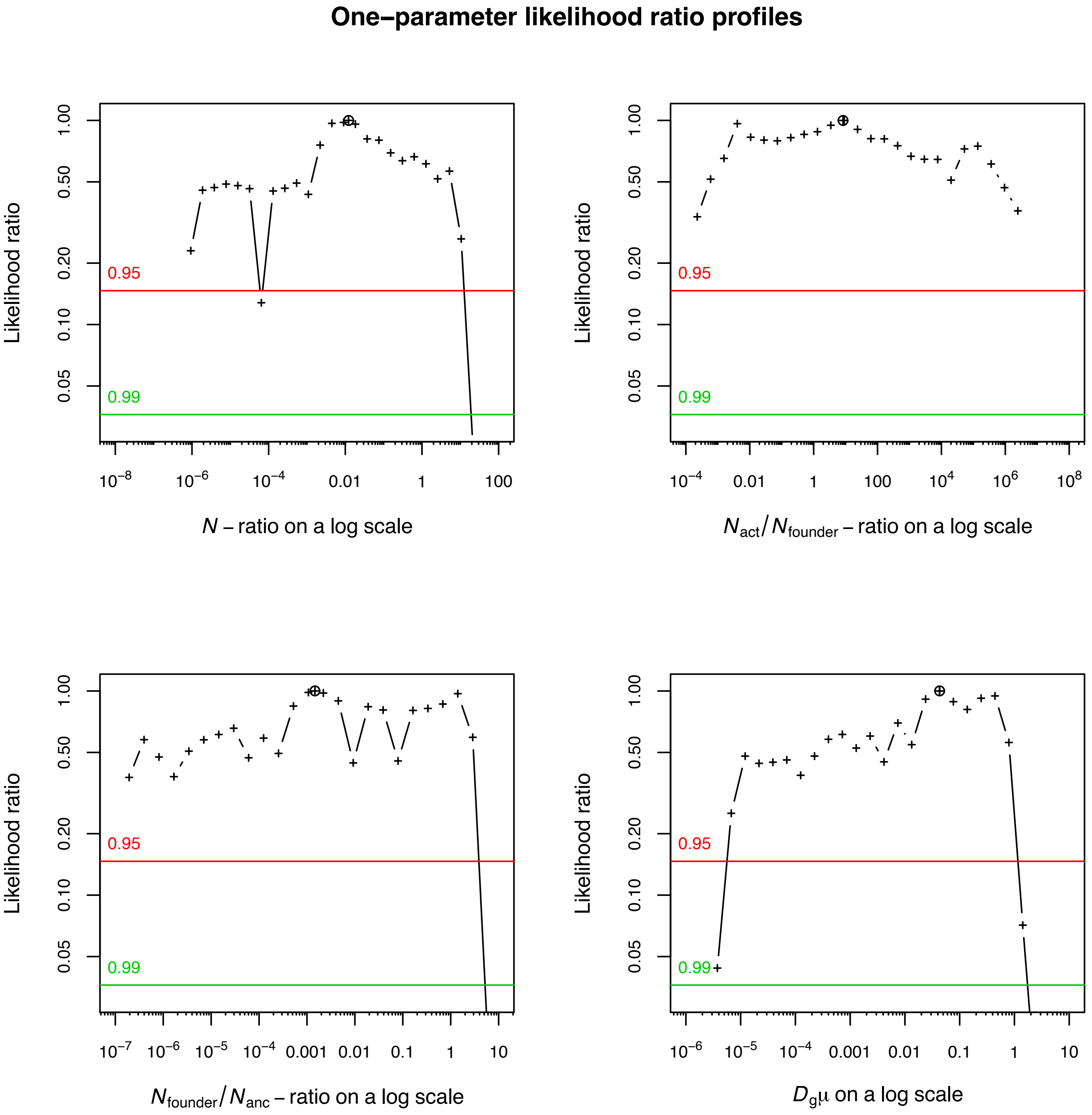

One–parameter likelihood ratio profiles
Likelihood ratio
1.00
0.50
0.20
0.10
0.05
0.95
0.99
10^{-8} 10^{-6} 10^{-4} 0.01 1 100
N – ratio on a log scale
10^{-4} 0.01 1 100 10^{4} 10^{6} 10^{8}
$N_{act}/N_{founder}$ – ratio on a log scale
10^{-7} 10^{-6} 10^{-5} 10^{-4} 0.001 0.01 0.1 1 10
$N_{founder}/N_{anc}$ – ratio on a log scale
10^{-6} 10^{-5} 10^{-4} 0.001 0.01 0.1 1 10
$D_g\mu$ on a log scale

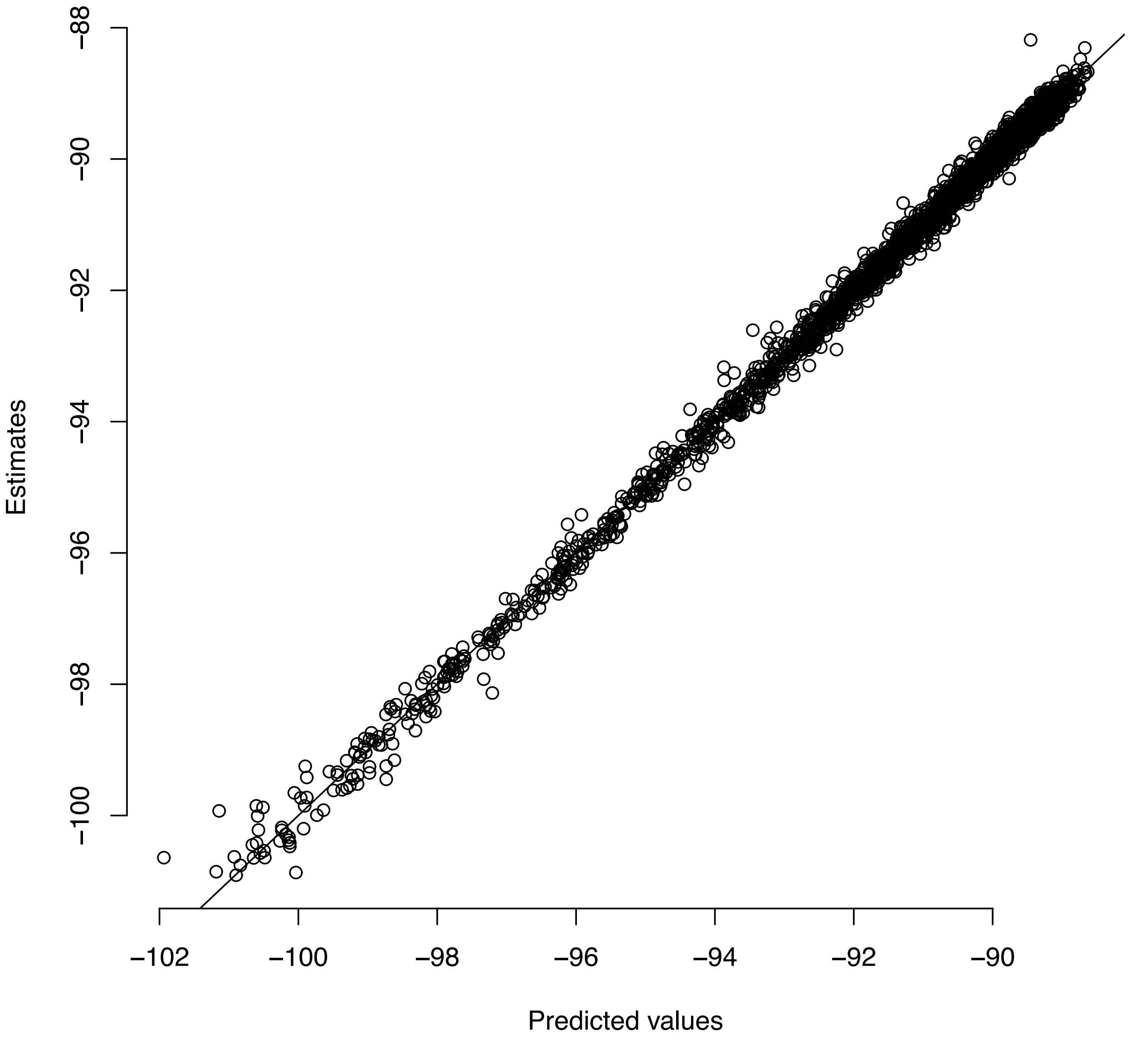
Estimates
−88
−90
−92
−94
−96
−98
−100
−102
−100
−98
−96
−94
−92
−90
Predicted values

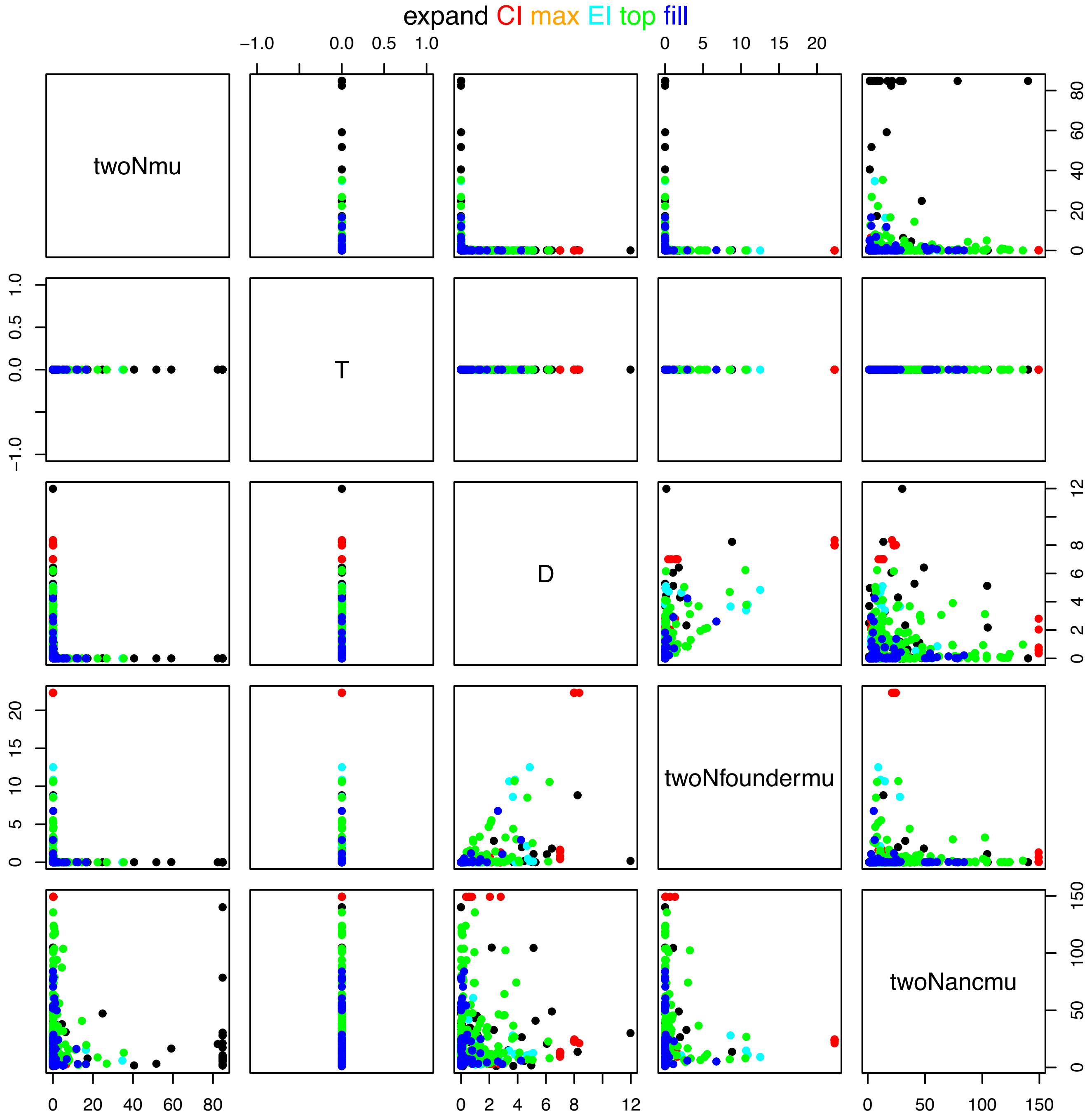

expand CI max EI top fill
twoNmu
T
D
twoNfoundermu
twoNancmu
−1.0
0.0
0.5
1.0
0
5
10
15
20
0
20
40
60
80
0
2
4
6
8
12
0
50
100
150
