# Supplementary material for: Origin and dispersion pathways of guava in the Galapagos Islands inferred through genetics and historical records
Source: Ecol Evol. 2021 Oct 4;11(21):15111–31. doi: 10.1002/ece3.8193 (PMC8571588; doi:10.1002/ece3.8193)
Supplement: Supplementary file 9 — Supplementary Material [file ECE3-11-15111-s009.docx]

**APPENDIX S1 - Origin and dispersion pathways of guava in the Galapagos Islands inferred through genetics and historical records**

**Tables**

### S1 TABLE. Priors employed in the ABC runs for determining the origin of the Isabela/Floreana and San Cristobal guava lineages in the mainland lineages. N1: Northern/Costal mainland lineage effective size, N2: Amazonian mainland lineage effective size, N3: Central mainland lineage effective size, N4: Southern mainland lineage effective size, N5: Dispersed mainland lineage effective size, N6: Current Isabela/Floreana or San Cristobal lineage effective size, t1: Divergence time of the Galapagos lineage from the mainland (in generations), db: population change duration (in generations), N6b: Isabela/Floreana or San Cristobal first guava colonizers (effective size), t2: Divergence time of all mainland lineages (in generations), NA: Mainland ancestral effective size, ra: Admixture rate (when applicable). For these ABC runs, the following considerations were taken: t2>=t1, db<=t1.

|  | **ABC for Isabela/Floreana lineage** | | **ABC for San Cristobal lineage** | |
| --- | --- | --- | --- | --- |
| **Parameter** | **Distribution** | **Values (min-max)** | **Distribution** | **Values (min-max)** |
| N1 | Uniform | 10.0-10,000.0 | Uniform | 10.0-10,000.0 |
| N2 | Uniform | 10.0-10,000.0 | Uniform | 10.0-10,000.0 |
| N3 | Uniform | 10.0-10,000.0 | Uniform | 10.0-10,000.0 |
| N4 | Uniform | 10.0-10,000.0 | Uniform | 10.0-10,000.0 |
| N5 | Uniform | 10.0-10,000.0 | Uniform | 10.0-10,000.0 |
| N6 | Uniform | 1.0-8,000.0 | Uniform | 0.5-8,000.0 |
| t1 | Uniform | 1.0-8,000.0 | Uniform | 0.01-8,000.0 |
| db | Uniform | 1.0-6,000.0 | Uniform | 0.005-6000.0 |
| N6b | Uniform | 1.0-10,000.0 | Uniform | 1.0-8,000.0 |
| t2 | Uniform | 1.0-10,000.0 | Uniform | 1.0-10,000.0 |
| NA | Uniform | 10.0-20,000.0 | Uniform | 10.0-20,000.0 |
| ra | Uniform | 0.001-0.999 | Uniform | 0.001-0.999 |

**S2 TABLE.** Priors employed in the 3 stages of ABC for retrieving the colonization pathway of guava once arrived in the Galapagos Islands. For the meaning of parameters’ abbreviation for each scenario in each stage see S1 File (for first stage scenarios), S2 File (for second stage scenarios) and Fig. 6 (for final model), as well as their legends.

| **Parameters** | **Distribution** | **Values (min-max)** |
| --- | --- | --- |
| **First Stage** | | |
| N1 | Uniform | 4,000.0-50,000.0 |
| N2 | Uniform | 1.0-8,000.0 |
| N3 | Uniform | 1.0-10,000.0 |
| N4 | Uniform | 1.0-8,000.0 |
| t1 | Uniform | 1.0-1,000.0 |
| db | Uniform | 1.0-10,000.0 |
| N3b | Uniform | 10.0-10,000.0 |
| t2 | Uniform | 1.0-2,000.0 |
| N4b | Uniform | 1.0-10,000.0 |
| t3 | Uniform | 1.0-10,000.0 |
| N2b | Uniform | 1.0-10,000.0 |
| ra | Uniform | 0.001-0.999 |
| **Second Stage** | | |
| N1 | Uniform | 4,000.0-50,000.0 |
| N2 | Uniform | 1.0-8,000.0 |
| N3 | Uniform | 1.0-8,000.0 |
| N4 | Uniform | 1.0-8,000.0 |
| t1 | Uniform | 1.0-2,000.0 |
| db | Uniform | 1.0-10,000.0 |
| N3b | Uniform | 1.0-8,000.0 |
| t2 | Uniform | 1.0-2,000.0 |
| N2b | Uniform | 1.0-8,000.0 |
| t3 | Uniform | 1.0-10,000.0 |
| N4b | Uniform | 1.0-8,000.0 |
| ra | Uniform | 0.001-0.999 |
| **Final Model** | | |
| N1 (Main.) | Uniform | 4,000.0-50,000.0 |
| N2 (ISA-Pres.) | Uniform | 1.0-8,000.0 |
| N3 (FLO-Pres.) | Uniform | 1.0-8,000.0 |
| N4 (SCZ-Pres.) | Uniform | 1.0-10,000.0 |
| N5 (SCY-Pres.) | Uniform | 1.0-8,000.0 |
| t1 | Uniform | 1.0-2,000.0 |
| db | Uniform | 1.0-2,000.0 |
| N4b (SCZ-Past) | Uniform | 1.0-10,000.0 |
| t2 | Uniform | 1.0-2,000.0 |
| N2b (ISA-Past) | Uniform | 1.0-8,000.0 |
| t3 | Uniform | 1.0-2,000.0 |
| N3b (FLO-Past) | Uniform | 1.0-8,000.0 |
| t4 | Uniform | 1.0-2,000.0 |
| N5b (SCY-Past) | Uniform | 1.0-8,000.0 |

### S3 TABLE. Results of the Hardy-Weinberg Equilibrium (HWE) test for each loci within the five populations (mainland, Isabela, Santa Cruz, San Cristobal and Floreana). Results shown correspond to those after B-Y correction.

|  | Mainland | Isabela | Santa Cruz | San Cristobal | Floreana | **Overall** |
| --- | --- | --- | --- | --- | --- | --- |
| mPgCIR10 | *** | *** | *** | >0.05 | *Monomorphic* | >0.05 |
| mPgCIR07 | *** | *** | *** | >0.05 | >0.05 | >0.05 |
| mPgCIR05 | *** | *** | *** | *** | >0.05 | >0.05 |
| mPgCIR17 | *** | *** | *** | *** | >0.05 | >0.05 |
| mPgCIR08 | *** | *** | * | * | >0.05 | >0.05 |
| mPgCIR11 | *** | *** | *** | *Monomorphic* | >0.05 | >0.05 |
| mPgCIR18 | *** | *** | *** | >0.05 | >0.05 | >0.05 |
| mPgCIR21 | *** | *** | *** | ** | >0.05 | >0.05 |
| mPgCIR09 | *** | *** | *** | >0.05 | >0.05 | >0.05 |
| mPgCIR22 | *** | >0.05 | *Monomorphic* | *Monomorphic* | *Monomorphic* | >0.05 |
| mPgCIR25 | *** | *** | *Monomorphic* | *Monomorphic* | *Monomorphic* | >0.05 |

Shows significance after BY correction of: < 0.05*, < 0.01** and < 0.001***

**S4 TABLE.**Estimate of null allele frequencies for each analyzed guava population (mainland Ecuador, Isabela, Santa Cruz, San Cristobal and Floreana) and SSR locus. The mean frequencies over the five populations are shown as well.

| **Locus** | **Mainland** | **Isabela** | **Santa Cruz** | **San Cristobal** | **Floreana** | **Mean** |
| --- | --- | --- | --- | --- | --- | --- |
| **mPgCIR10** | 0.322 | 0.127 | 0.131 | 0.041 | 0.001 | 0.124 |
| **mPgCIR07** | 0.278 | 0.233 | 0.188 | 0.082 | 0.195 | 0.195 |
| **mPgCIR05** | 0.273 | 0.147 | 0.150 | 0.198 | 0.260 | 0.206 |
| **mPgCIR17** | 0.250 | 0.239 | 0.179 | 0.178 | 0.000 | 0.169 |
| **mPgCIR08** | 0.263 | 0.138 | 0.113 | 0.069 | 0.154 | 0.147 |
| **mPgCIR11** | 0.173 | 0.255 | 0.218 | 0.001 | 0.000 | 0.129 |
| **mPgCIR18** | 0.258 | 0.160 | 0.179 | 0.098 | 0.260 | 0.191 |
| **mPgCIR21** | 0.327 | 0.153 | 0.209 | 0.193 | 0.213 | 0.219 |
| **mPgCIR09** | 0.254 | 0.159 | 0.202 | 0.035 | 0.195 | 0.169 |
| **mPgCIR22** | 0.283 | 0.028 | 0.001 | 0.001 | 0.001 | 0.063 |
| **mPgCIR25** | 0.297 | 0.190 | 0.000 | 0.001 | 0.001 | 0.098 |
| **Mean** | 0.271 | 0.166 | 0.143 | 0.082 | 0.116 | 0.155 |

**S5 TABLE.** Genetic diversity basic statistics of the guavas from the 9 mainland Ecuador regions sampled: Number of individuals genotyped from each island (N), number of alleles found (A), mean allelic richness (AR), number of private alleles (PA), observed heterozygosity (H_O_), expected heterozygosity/gene diversity (H_E_) and inbreeding coefficient (F_IS_).

| **Population** | **N** | **A** | **AR^s^** | **PA*** | **H_O_** | **H_E_ ^NA^** | **F_IS_ ^NA^** |
| --- | --- | --- | --- | --- | --- | --- | --- |
| North Coast (NC) | 8 | 38 | 3.45 | 3 (3) | 0.119 | 0.601  (0.484) | 0.802  (0.822) |
| North Highlands (NH) | 13 | 53 | 4.39 | 3 (2) | 0.205 | 0.710  (0.631) | 0.711  (0.702) |
| North Amazon (NA) | 12 | 62 | 5.17 | 18 (7) | 0.428 | 0.738  (0.698) | 0.420  (0.406) |
| Central Coast (CC) | 11 | 56 | 4.73 | 8 (5) | 0.190 | 0.697  (0.627) | 0.727  (0.740) |
| Central Highlands (CH) | 11 | 61 | 5.04 | 8 (5) | 0.339 | 0.727  (0.657) | 0.534  (0.536) |
| Central Amazon (CA) | 8 | 59 | 5.36 | 8 (8) | 0.482 | 0.723  (0.683) | 0.333  (0.330) |
| South Coast (SC) | 11 | 61 | 5.23 | 5 (3) | 0.278 | 0.772  (0.725) | 0.640  (0.648) |
| South Highlands (SH) | 12 | 67 | 5.31 | 15 (6) | 0.317 | 0.757  (0.705) | 0.581  (0.580) |
| South Amazon (SA) | 10 | 60 | 5.15 | 14 (10) | 0.266 | 0.749  (0.683) | 0.645  (0.645) |

^s^ standardized through rarefaction for N=16 genes

* Values between brackets are the number of private alleles following rarefaction for N=16 genes

^NA^ The values outside the brackets correspond to H_E_ or F_IS_ under null allele correction. The values between brackets are values without null allele correction.

**S6 TABLE.** Pairwise F_ST_ values between all pairs of mainland Ecuador regions and Galapagos populations of guava. The F_ST_ values shown here were null allele-corrected using the ENA algorithm. Mainland Ecuador regions are: North Coast (NC), North Highlands (NH), North Amazon (NA), Central Coast (CC), Central Highlands (CH), Central Amazon (CA), South Coast (SC), South Highlands (SH), and South Amazon (SA). Galapagos populations are: Isabela (ISA), Santa Cruz (SCZ), San Cristobal (SCY), and Floreana (FLO).

|  | **NC** | **NH** | **NA** | **CC** | **CH** | **CA** | **SC** | **SH** | **SA** | **ISA** | **SCZ** | **SCY** |
| --- | --- | --- | --- | --- | --- | --- | --- | --- | --- | --- | --- | --- |
| **NH** | 0.054 |  |  |  |  |  |  |  |  |  |  |  |
| **NA** | 0.067 | 0.023 |  |  |  |  |  |  |  |  |  |  |
| **CC** | 0.036 | 0.049 | 0.050 |  |  |  |  |  |  |  |  |  |
| **CH** | 0.122 | 0.080 | 0.091 | 0.012 |  |  |  |  |  |  |  |  |
| **CA** | 0.088 | 0.069 | 0.027 | 0.059 | 0.075 |  |  |  |  |  |  |  |
| **SC** | 0.098 | 0.068 | 0.073 | 0.067 | 0.065 | 0.073 |  |  |  |  |  |  |
| **SH** | 0.129 | 0.089 | 0.086 | 0.061 | 0.036 | 0.087 | 0.032 |  |  |  |  |  |
| **SA** | 0.183 | 0.115 | 0.110 | 0.107 | 0.077 | 0.112 | 0.054 | 0.036 |  |  |  |  |
| **ISA** | 0.504 | 0.468 | 0.476 | 0.434 | 0.404 | 0.467 | 0.413 | 0.415 | 0.447 |  |  |  |
| **SCZ** | 0.427 | 0.395 | 0.420 | 0.364 | 0.356 | 0.403 | 0.368 | 0.378 | 0.392 | 0.096 |  |  |
| **SCY** | 0.454 | 0.432 | 0.465 | 0.393 | 0.392 | 0.450 | 0.426 | 0.440 | 0.462 | 0.183 | 0.076 |  |
| **FLO** | 0.602 | 0.522 | 0.503 | 0.503 | 0.451 | 0.510 | 0.458 | 0.436 | 0.488 | 0.091 | 0.185 | 0.329 |

**Figures**

**
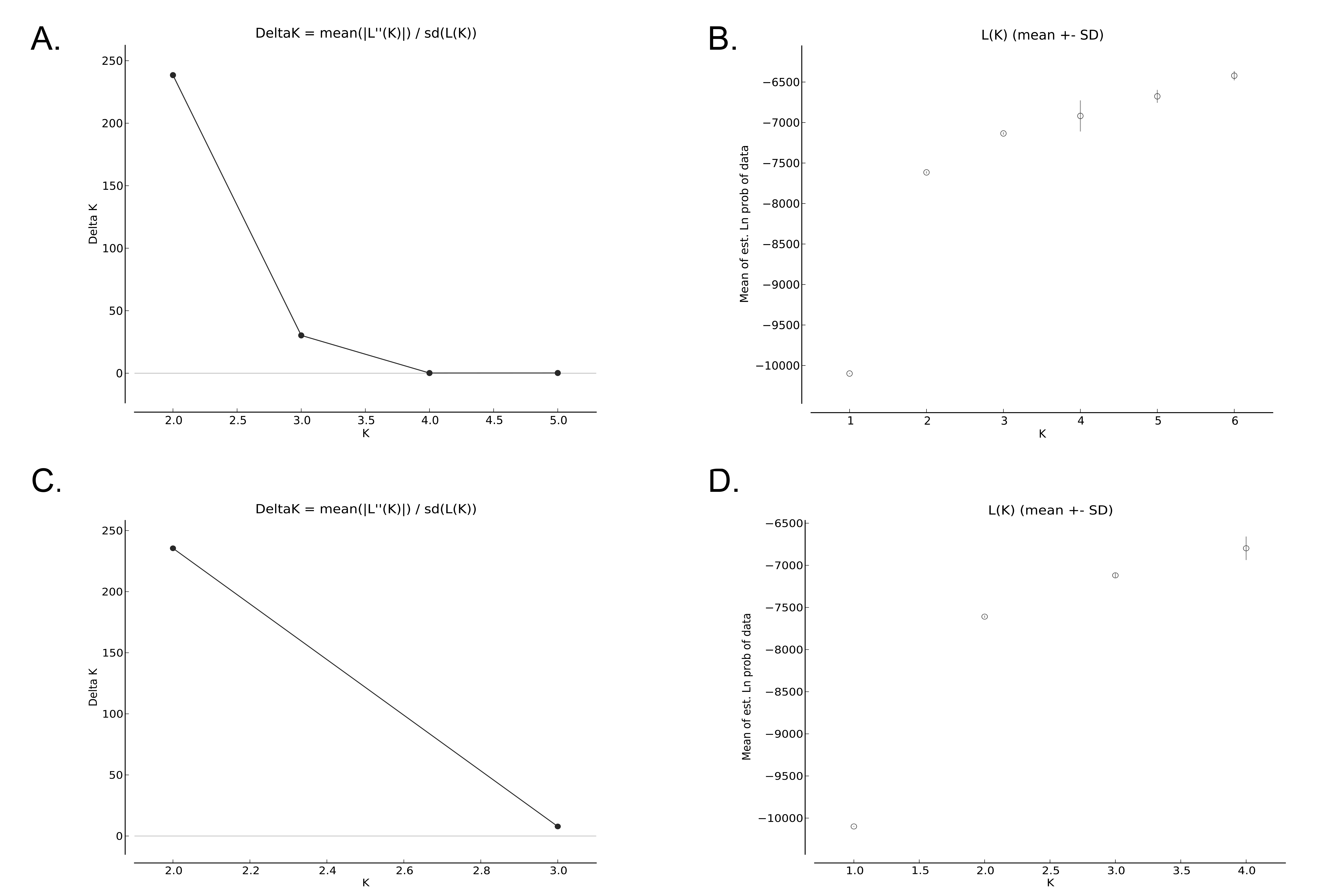
**

**S1 Figure.** A. Number of lineages (K) vs. Delta K (ΔK) plot for selecting the best K value in the preliminary STRUCTURE analysis for the full data set (Galapagos + mainland populations); the plot shows a peak corresponding to K=2, which would be the selected best K value according the method used (Evanno method implemented in Structure Harvester). B. Plot showing the mean estimated Ln. probabilities for each K value at the preliminary analysis. C. Number of lineages (K) vs. Delta K (ΔK) plot for selecting the best K value in the final STRUCTURE analysis for the full data set (Galapagos + mainland populations); the plot shows a peak corresponding to K=2, which confirms the selected best K value. D. Plot showing the mean estimated Ln. probabilities for each K value at the final analysis.

**
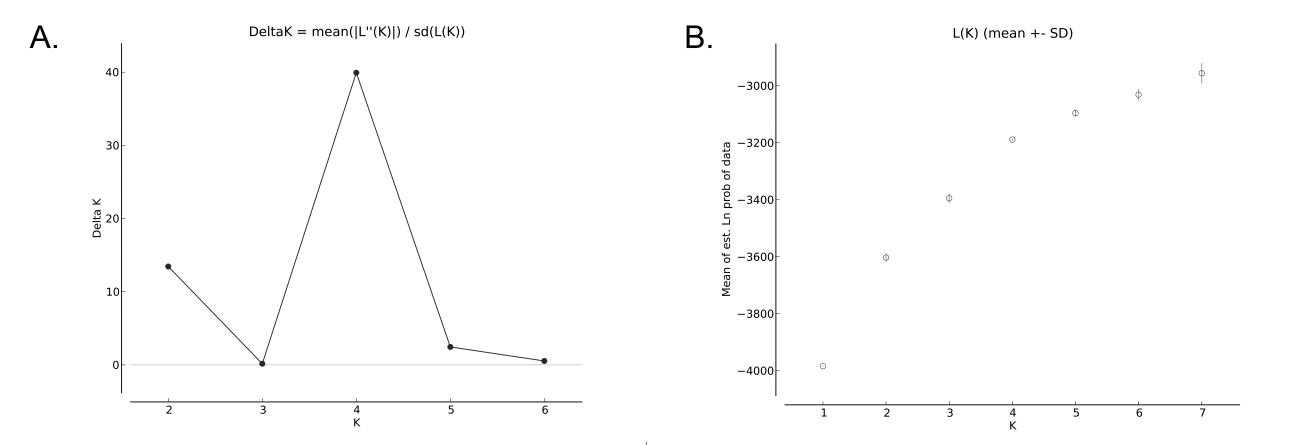
**

**S2 Figure.** A. Number of lineages (K) vs. Delta K (ΔK) plot for selecting the best K value in the STRUCTURE analysis for the mainland Ecuador population; the plot shows a peak corresponding to K=4, which would be the selected best K value according the method used (Evanno method implemented in Structure Harvester). B. Plot showing the mean estimated Ln. probabilities for each K value at the same analysis.

**S3 Figure.** PCA model checking of the best supported scenario (Admixture: Central + Southern lineages) for the ABC analysis addressing the origin of the Isabela/Floreana lineage in the 5 mainland lineages defined by the Bayesian analysis performed in the STRUCTURE software.

**S4 Figure.** PCA model checking of the final scenario proposed for the colonization history of guava in the Galapagos Islands following ABC analysis.

**Supplementary Files Legends**

**S1 FILE.** Diagrams of the initial 16 scenarios tested through ABC analysis, in the first stage of the analysis to infer the history of colonization of guava in the Galapagos Islands. Time is not shown to scale and is measured as number of generations, considering t3> = t2, t3> = t1, t2> = t1 and db<t3. Pop1/N1: Current mainland guava population, Pop2/N2: Current Isabela/Floreana population, Pop3/N3: Current Santa Cruz population, Pop4/N4: Current San Cristobal population; N2b: Isabela/Floreana first guava colonizers, N3b: Santa Cruz first colonizers, N4b: San Cristobal first colonizers.

**S2 FILE.** Diagrams of the 9 scenarios tested in the second stage of our ABC analysis to infer the history of colonization of guava in the Galapagos Islands. Time is not shown to scale and is measured as number of generations, considering t3> = t2, t3> = t1, t2> = t1 and db<t3. Pop1/N1: Current mainland guava population, Pop2/N2: Current Isabela population, Pop3/N3: Current Floreana population, Pop4/N4: Current San Cristobal population; N2b: Isabela first guava colonizers, N3b: Floreana first colonizers, N4b: San Cristobal first colonizers.

Note: DIYABC was unable to generate the diagrams for the Scenarios 5 and 6, so the DIYABC script for these scenarios is shown instead.

**S3 FILE.** *Migraine* graphical outputs corresponding the Isabela guava population analysis under the OnePopFounderFlush demographic model (SMM). File includes the Pairwise likelihood-ratio profiles and diagnostic plots for validating our results and inferences.

**S4 FILE.** *Migraine* graphical outputs corresponding the Santa Cruz guava population analysis under the OnePopFounderFlush demographic model (SMM). File includes the Pairwise likelihood-ratio profiles and diagnostic plots for validating our results and inferences.

**S5 FILE.** *Migraine* graphical outputs corresponding the San Cristobal guava population analysis under the OnePopFounderFlush demographic model (SMM). File includes the Pairwise likelihood-ratio profiles and diagnostic plots for validating our results and inferences.

**S6 FILE.** *Migraine* graphical outputs corresponding the Floreana guava population analysis under the OnePopFounderFlush demographic model (SMM). File includes the Pairwise likelihood-ratio profiles and diagnostic plots for validating our results and inferences.

**S7 FILE.** Posterior distributions of the parameters of the best supported scenario (Admixture: Central + Southern lineages) for the ABC analysis addressing the origin of the Isabela/Floreana lineage in the 5 mainland lineages defined by the Bayesian analysis performed in STRUCTURE.

**S8 FILE.** Posterior distributions of the parameters of the final model proposed for the introduction and colonization history of guava in the Galapagos Islands inferred from the ABC analysis.

**…**
